# Methods to capture proteomic and metabolomic signatures from cerebrospinal fluid and serum of healthy individuals

**DOI:** 10.1038/s41598-022-16598-1

**Published:** 2022-08-03

**Authors:** Laura M. Lilley, Steven Sanche, Shepard C. Moore, Michelle R. Salemi, Dung Vu, Srinivas Iyer, Nicolas W. Hengartner, Harshini Mukundan

**Affiliations:** 1 Los Alamos National Laboratory, P.O. Box 1663, Los Alamos, NM 87545 USA; 2 Genome Center, Proteomics Core Facility, University of California, Davis, CA 95616 USA

**Keywords:** Biochemistry, Neuroscience, Medical research

## Abstract

Discovery of reliable signatures for the empirical diagnosis of neurological diseases—both infectious and non-infectious—remains unrealized. One of the primary challenges encountered in such studies is the lack of a comprehensive database representative of a signature background that exists in healthy individuals, and against which an aberrant event can be assessed. For neurological insults and injuries, it is important to understand the normal profile in the neuronal (cerebrospinal fluid) and systemic fluids (e.g., blood). Here, we present the first comparative multi-omic human database of signatures derived from a population of 30 individuals (15 males, 15 females, 23–74 years) of serum and cerebrospinal fluid. In addition to empirical signatures, we also assigned common pathways between serum and CSF. Together, our findings provide a cohort against which aberrant signature profiles in individuals with neurological injuries/disease can be assessed—providing a pathway for comprehensive diagnostics and therapeutics discovery.

## Introduction

Insult or injury to the body—as mediated by a variety of non-infectious conditions such as neurodegenerative diseases, stroke, and blunt force head trauma– modifies normal physiological and biochemical function^1–4^. Identification of specific signature patterns reflective of the insult or injury can facilitate the development of empirical diagnostics and targeted therapeutics^5^. For instance, current diagnostics for mild traumatic brain injury (TBI) rely on neuropsychological questionnaires and imaging strategies for qualitative identification^6,7^. The effectiveness of these diagnostics is limited by varied presentation of disease state, delayed onset of symptoms, comorbidities, clinical history, and differential long-term presentation, a limitation that can be overcome by the availability of empirical diagnostics.

**Figure 1:**
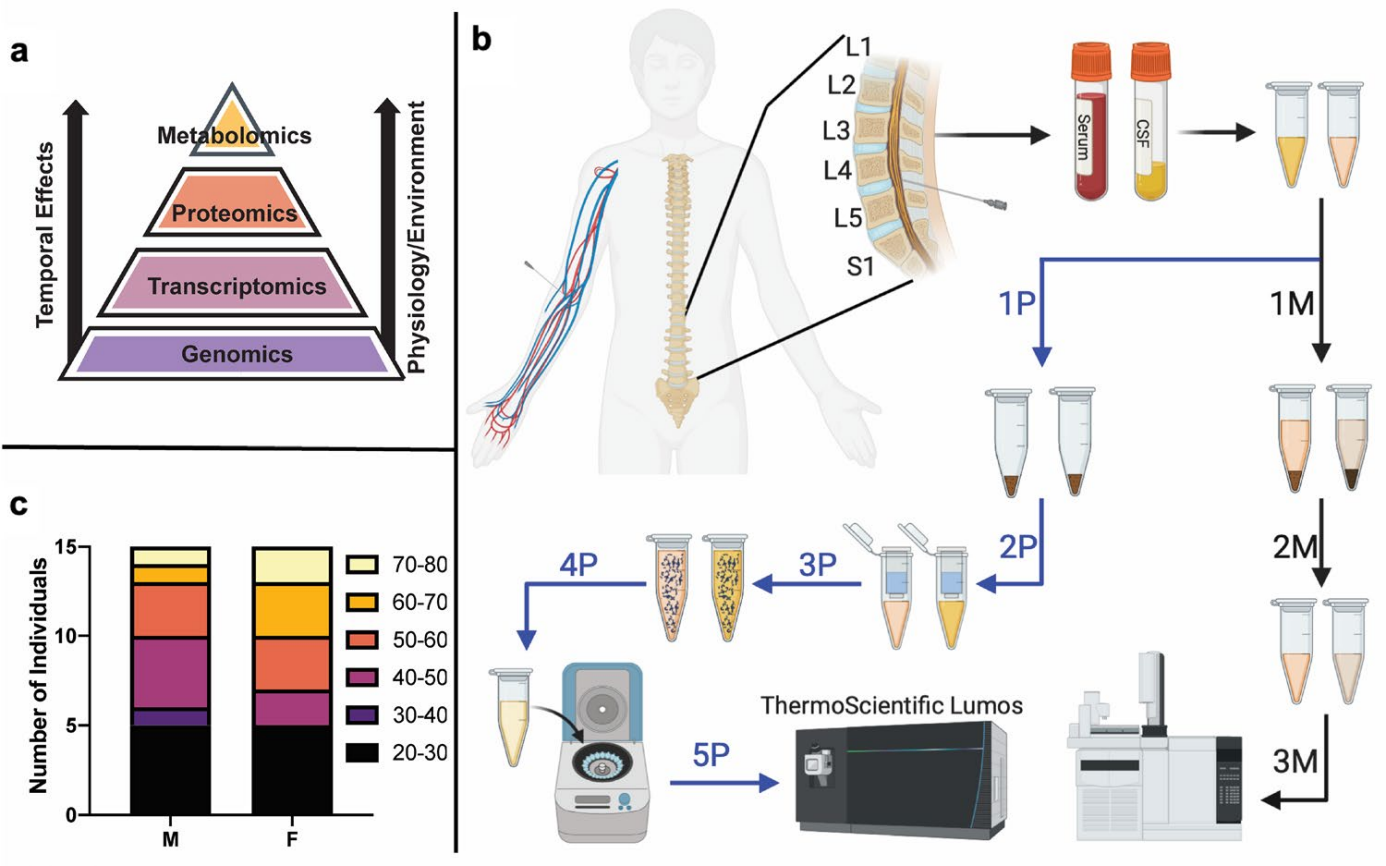
(**a**) Hierarchy of ‘omics where the metabolome and proteome are temporally more sensitive to environmental influence such as disease and injury. (**b**) Sample acquisition and processing scheme for Proteomics (left) and metabolomics (right) CSF samples (blue icons) were drawn by lumbar puncture (S1–L5) and serum samples (red icons) were drawn by venipuncture. Proteomics processing follows the left workflow with lyophilization (1P), resuspension and solubilization (2P), proteins were adhered to the S-Trap column (3P), the fixed proteins were washed (4P), proteins were digested overnight with trypsin (5P), the peptides were eluted (6P), dried and suspended for LC–MS/MS analysis (7P). Metabolomics processing follows the right workflow by organic solvent extraction and separation (1 M), followed by concentration and resuspension (2 M) for GC–MS analysis (3 M). C) Age/sex breakdown of the 30 CSF and 30 serum samples.

Derivation of empirical diagnostic signatures for a given disease state requires a systems level understanding of the processes involved. The ‘omics revolution has enabled faster, cheaper, and higher-throughput analyses of genes, proteins, and metabolites facilitating identification of new targets for a variety of diseases^8,9^. Where the genome is relatively resilient to external environmental influences, the human proteome and metabolome are more susceptible to environment and injury, making them ideal signatures for diagnostics development (Fig. 1a). Multi-omic studies have led to the identification of several biomarkers associated with a variety of diseases such as TBI^10–13^. However, widespread clinical diagnostic development from such studies has been limited owing to intrinsic variability in observed biomarker profiles. One of the primary limitations hampering clinical translation of multi-omic observables is the biomarker patterns associated with a disease/injury are not cases of simple presence/absence, they must be coupled with a threshold concentration in the sample of interest (e.g., blood, cerebrospinal fluid, or urine). That threshold is challenging to determine without a reliable baseline under healthy conditions. Such a baseline should account for the variability in a given biomarker among individuals in a population (e.g., age/sex). Further, even within an individual- a biomarker profile measured is a “snapshot” of the current biochemical state and will vary with time in response to external influences^14^. The availability of systematic, reliable, baseline signature profiles of healthy individuals that accounts for inter- and intra- individual variability is essential for assessing and characterizing disease-specific biomarker expression^15^. The work presented herein aims to advance us a step further in that direction.

The brain is the most lipid-rich organ and consumes about 20% of the body’s total energy^16,17^. Thus, insults and injuries to the brain (e.g. TBI), and the associated disruption of blood supply can generate a metabolic crisis that, if unresolved, can increase brain atrophy and worsen outcomes^18^. When disrupted, CSF leaks into the blood thus, biomarkers *normally* exclusive to the brain but found in blood can yield information about the biochemical status of injury and disease. However, there are limited comprehensive (the only report currently published by Dayon et al.) studies simultaneously comparing the proteomic and metabolomic profiles of matched CSF and serum samples within individual patients^19^. Such comparisons are complicated by the fact that the native comparative multi-omic signature profile of CSF and serum in health individuals are not well defined. Further, CSF is a highly dynamic fluid and sample acquisition can give varied results depending whether CSF is obtained from the spinal fluid or directly via shunt the ventricular system^20^.

Herein, we present a comparative proteomic and metabolomic study of matched CSF/serum from 30 individuals with no previously documented adverse neurological conditions or ailments, to alleviate some of the above challenges associated with biomarker discovery for neurological insults. Figure 1b details the sample collection and processing of serum samples separated from blood collected by venipuncture and CSF samples were collected by lumbar puncture (L1-S1 vertebra). Aliquots of these matched CSF/serum were processed for proteomic and lipidomic profiling. Our population consisted of 15 females and 15 males ranging in age from 23 to 74 years (Fig. 1c).

### Identifying proteins

In biomarker discovery, depletion of high-abundance proteins such as immunoglobulins and albumin (dg/L) to facilitate examination of lower abundance proteins (ng/L)^21–25^. However, in our initial scoping experiments, we found these depletion procedures contributed to high variance in the detected proteome, both in repeat measurements of a given sample and among similar samples. Therefore, we chose to sacrifice sensitivity to reliably detect proteins in very low concentration for reduced variability in the measured proteomic profiles. Proteins were cleaned up and digested on an S-Trap column then analyzed by LC–MS/MS on a Thermo Scientific Fusion Lumos platform running in Data Independent Acquisition (DIA) mode (Fig. 1b). Chromatogram library samples were individually searched against Prosit predicted databases and converted for ScaffoldDIA using a reference spectral library created in EncyclopeDIA v.0.9.2 (details in the Methods). Proteins were identified at a 10% false discovery rate (FDR) and minimum of one peptide.

**Figure 2:**
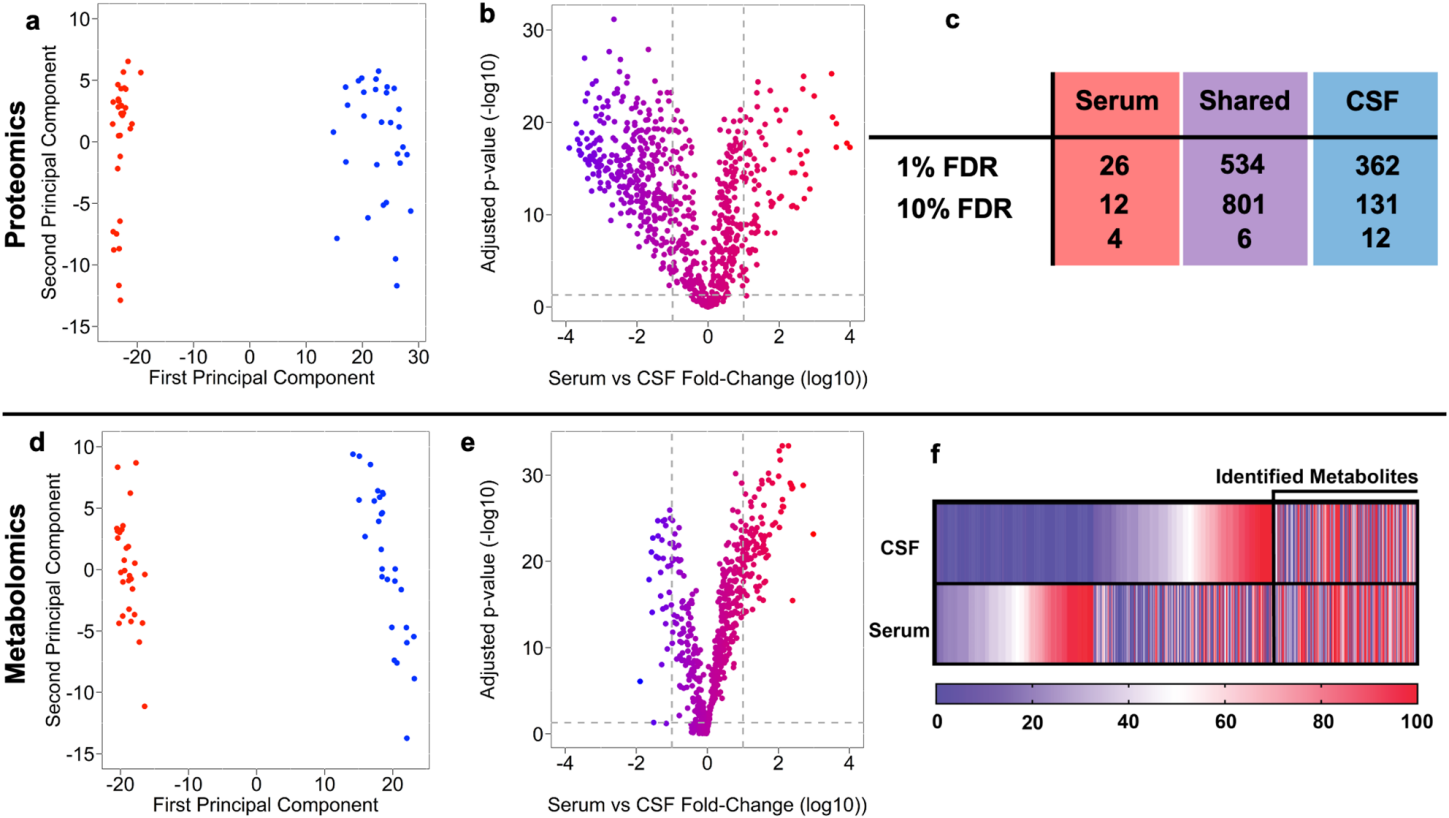
(**a**) Principal component analysis of proteomics data serum proteins (red) and CSF proteins (blue) where each point is a sample. (**b**) Volcano plot of proteomics data where the log_10_ of each protein’s intensity versus − log_10_ corrected p-value the vertical dashed grey lines represent a +/− tenfold change and the horizontal dashed line is *p* = 0.05. (**c**) Table describing how the proteins included in the analysis vary by FDR rate. (**d**) Principal component analysis of metabolomics data. (**e**) Volcano plot of metabolomics data. (**f**) Heatmap of metabolites covered in the analysis, metabolites right of the black line represents the metabolites positively identified. rThe 0–100 scale on the heatmap represents normalized percentage of being detected in either CSF or serum.

Under these conditions, we identified 813 proteins in serum and 932 in CSF. Further, 801 proteins were shared between both samples, 12 proteins were unique to serum and 131 in CSF. The intensity of fragment ions was used to measure relative abundance between CSF and serum. The total variance in intensity across proteins of the pooled CSF and serum samples was decomposed using Principal Component Analysis (PCA). That analysis revealed that the largest contributions to the variance of the pooled sample is the sample label, CSF vs. serum, which explains 56% of the total variance (Fig. 2a). In contrast, the second principal component only explained a small fraction of the total variance (2%). Figure 2b illustrates the relative differences in mean protein abundance between CSF and serum (x-axis) as a function of its associated Benjamini–Hochberg adjusted p-values (y-axis). Each point on the figure represents one of the 801 proteins identified in CSF/Serum. 317 proteins were significantly more abundant in CSF, with a tenfold or greater difference in intensity. In comparison, 83 proteins were significantly more abundant in serum with a tenfold or more difference in intensity. In this study, we explicitly demonstrate how changing the FDR changes unique protein coverage between serum and CSF (Fig. 2c, complete list in S1–S2). The number of proteins unique to each sample type decreases substantially with increasing FDR. We chose an FDR of 10% for this study as it balanced sensitivity and predictive accuracy.

### Identifying metabolites

Understanding the basic metabolomic profiles under healthy, uninjured conditions can help underpin the normal relationship of metabolic signatures between serum and CSF within a given individual. Here, we detail the first matched comparative human CSF/serum metabolome. Metabolites were extracted with methyltertbuyl ether and methanol separated from the proteins, and derivatized for GC-TOF MS analysis using an Agilent 6890 GC and a Pegasus III TOF MS (full details in the Methods)^26^. Metabolites were identified and quantified using BinBase v 4.0^27–29^. A total of 613 metabolites were identified across all samples. BinBase does analyze data as a function of FDR therefore, we compared CSF/serum in terms of relative MS abundance. For this, we applied similar statistical procedures as was described for identified proteins (PCA and t-tests). Figure 2d illustrates the first two principal components coordinates of the samples. Similarly, to the above results, the first principal component explained a large part of the total variance (58%). This variance also appeared to be largely due to differences between sample types (CSF vs. serum). The second principal explained only 6% of the total variance. 29 metabolites were significantly (> 10X) more abundant in CSF, while 110 metabolites were significantly (> 10X) more abundant in serum (Fig. 2e). Metabolomics databases are immature thus, the number of metabolites that can be positively assigned represents a small fraction of the total number of detected compounds. Figure 2f illustrates the small fraction (182) of detected metabolites that could be assigned compound identity. A complete annotated list of identified and BinBase metabolites can be found in S5.

### Demographic analysis

**Figure 3:**
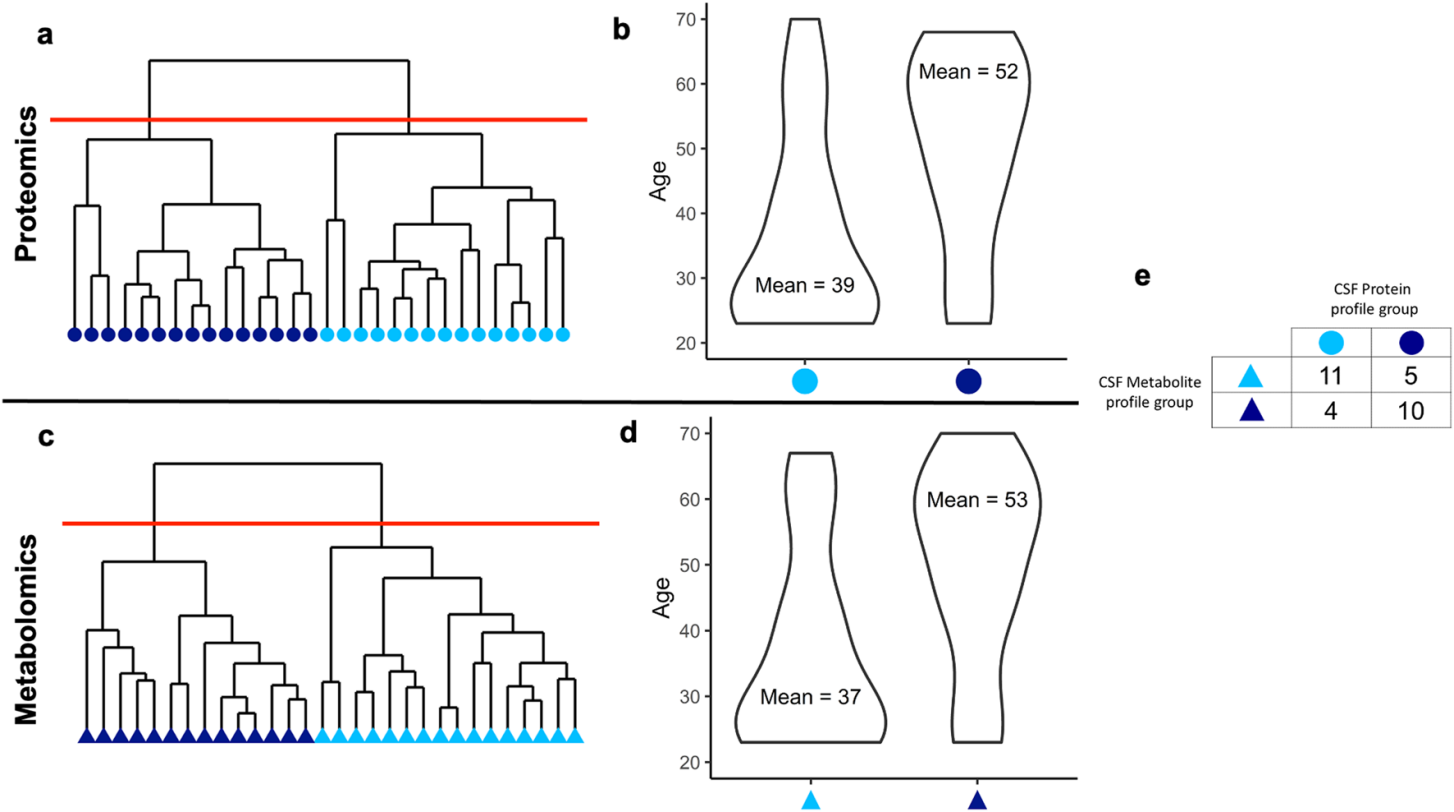
(**a**) Clustering diagram of CSF proteomics , each point represents an individual clustered on the principal components with Ward hierarchical clustering. The red line indicates where the tree was cut to form clusters. Longer branches represent larger separations between groups of individuals. (**b**) Violin plot of age in terms of CSF proteomics clusters. (**c**) Clustering diagram of CSF metabolome, each point represents an individual clustered on the principal components with Ward hierarchical clustering. The red line indicates where the tree was cut to form clusters. (**d**) Violin plot of age in terms of CSF metabolomics clusters (**e**) Cross-tabulation of CSF proteomics and metabolomics membership.

The impact of age and gender on variations in the proteomic and metabolomic profiles were assessed. For this, a principal component analysis (on the first 10 components) of the proteome and metabolome, assessing differences between CSF *vs* serum mapped onto each individual was performed. Ward hierarchical clustering of individuals revealed two subgroups within the CSF proteome and metabolome among healthy individuals. Examination of the demographics of individuals in these subgroups show that they differed on the basis of age (Fig. 3a, c). For the CSF proteome, the two groups averaged 39 years and 52 years in age, respectively (*p* = 0.04), while for the metabolome the groups had an average of 37 versus 53 years of age (*p* = 0.005). While membership to subgroup in proteome and metabolome are strongly positively related, there are individuals whose subgroup assignment are discordant (Fig. 3b, d). Notable neuronal proteins that differ based on age include apolipoprotein E, neuronal pentraxin-1, and reticulon-4. A table comprising the individual proteins (S3) and metabolites (S4) that differ between groups can be found in the SI.

### Comparisons of neurological proteins of interest

**Figure 4:**
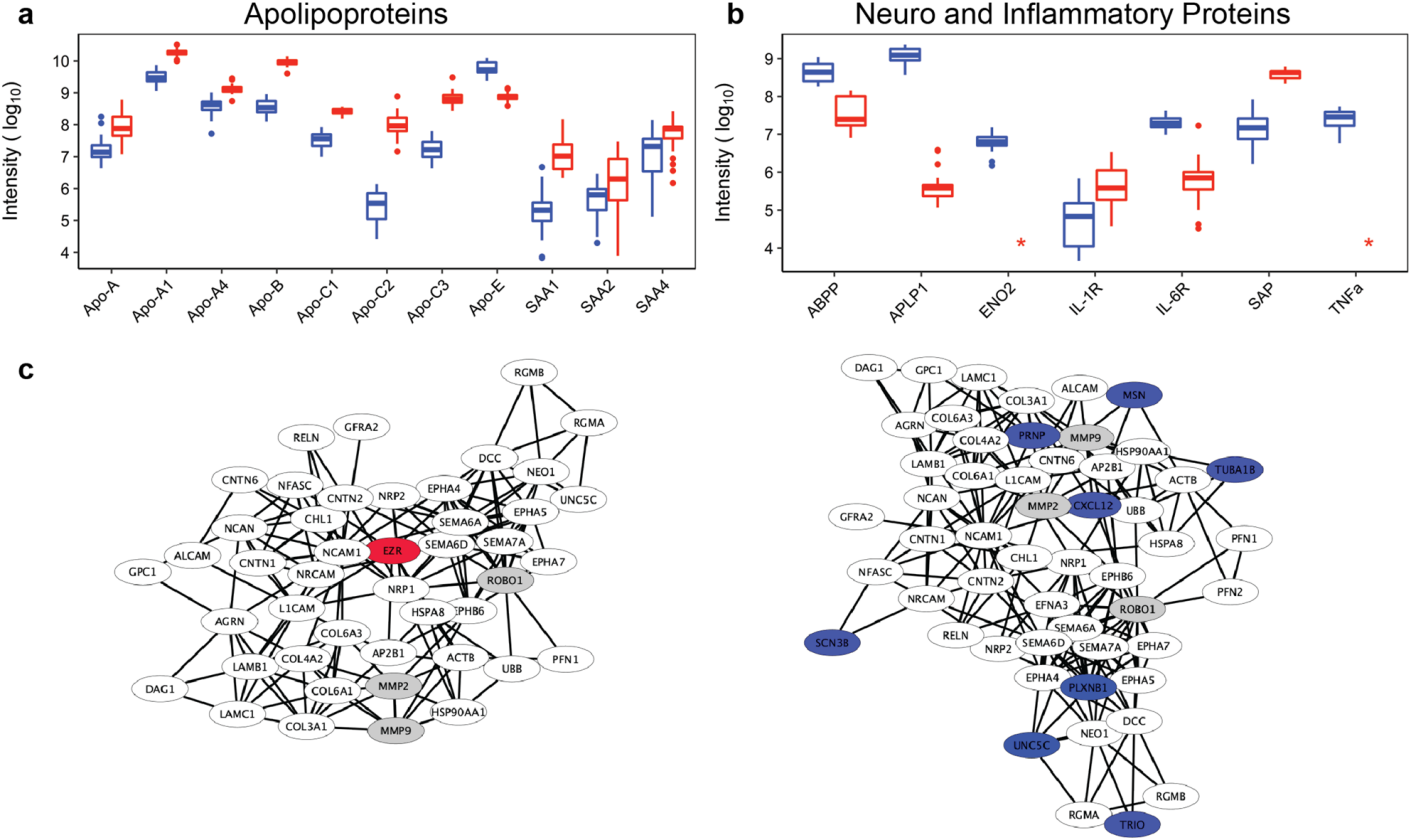
(**a**) Boxplots of apolipoprotein intensity and comparison between CSF (blue) and serum (red). Serum amyloid A proteins (SAA#) and apolipoproteins (Apo-letter). Apolipoproteins are generally more abundant in serum over CSF with the exception of Apo-E. (**b**) Boxplots of important detected neuro and inflammatory proteins serum amyloid-P and P1 (SAP), amyloid precursor protein (ABPP), Interleukins 1 and 6 (IL-1 and IL-6), γ-enolase (ENO2). (**c**) STRING over-representation analysis of axon guidance. Proteins marked in grey control multiple biochemical pathways and were common in our over-representation analysis. Proteins marked in white are common to CSF and serum, proteins marked in blue and unique to CSF, and the protein marked in red is unique to serum.

Several biomarkers and physiological processes have been implicated in the pathology of neuronal insults and injury. Yet, many of these signatures are expressed in healthy cells—albeit at different concentrations than injured ones. In this section, we examine the relative distribution for some of these signatures in healthy CSF and serum, to establish a baseline for their expression and consequent extrapolation of change in insult and injury. Specifically, we compare MS intensities between apolipoproteins (Fig. 4a) and important neuroproteins in CSF and serum (Fig. 4b)^10,30^. Apolipoproteins, particularly Apo-E (P02649) are implicated in a variety diseases from cardiovascular, neurodegenerative and TBI^31,32^. We did not find any significant differences in these proteins based on the gender of the individuals found in another modern report^33^. Apo-E is produced in the liver by hepatocytes and in the brain, and is the seventh major protein in CSF. Indeed, we found that Apo-E is expressed in significantly (10X) higher abundance in CSF over serum (Fig. 4a). Serum amyloid A1, A2, and A4 (SAA) (P0DJI8, P0DJI9, P35542) are constitutively expressed apolipoproteins that change expression in response to cytokine induced inflammation (IL-1, IL-6, IL-8, and TNFα). These proteins have been implicated to vary during the course of TBI, as a function of gender and with larger cohorts^19,34^. In accordance, our findings indicate constitutive SAA levels are higher in serum over CSF, as expected for healthy individuals. However, contrary to other findings, we found no baseline difference in SAAs between males and females. Further comparison of Apo-A, Apo-B, and Apo-C revealed expected trends of higher baseline concentrations in serum over CSF, where these proteins are associated with host lipoproteins such as HDL, LDL, and VLDL. Other relevant insult and injury markers (Fig. 4b) observed in healthy CSF and serum include (1) IL-6 receptor subunit beta (P40189) present in greater abundance in CSF—activator of JAK-MAPK and JAK-STAT3 signaling, (2) the IL1 receptor accessory protein (Q9NPH3)—part of the IL-33 signaling system responsible for the pre- and postsynaptic differentiation of neurons, (3) serum amyloid P (P02743)—related to amyloidosis and aggregation in plaques, (4) amyloid-like protein 1 (P51693)—part of postsynaptic function and a transcriptional regulator, (5) amyloid precursor protein (P05067)—a metal binding protein important for axiogenesis, synaptogenesis, neuronal growth, and adhesion (among other functions), and 6) γ-enolase (P09104)—a highly important neuroprotective/neurotrophic enzyme with a broad range of biochemical functions was found exclusively in CSF.

Over-representation pathway-based analyses using bioinformatics tools are useful to identify patterns of proteins associated with known biochemical functions. Here, we used STRING of the proteins associated with *axon guidance* FDR = 2.14E−7 (Reactome R-HSA-422475) in CSF and serum are presented in Fig. 4c. This analysis takes co-occurrence, co-expression, direct experimental evidence, text mining, and database evidence to generate the clustering set at the highest confidence limit (0.9). Each node represents a single protein and the lines connecting the nodes are associated confidence. Proteins marked in grey—matrix metalloproteinase (MMP) 2/9, and roundabout homolog 1 (ROBO1)—control multiple biochemical pathways and were common in our over-representation analysis. Proteins detected in CSF that were *not* detected in serum in the present study include; moesin (MSN, P26038), major prion protein (PRNP, P04156), stromal cell-derived factor 1 (CXCL12, P48061), tubulin alpha-1B chain (TUBA1B, P68363), triple function domain protein (TRIO, O75962), sodium channel subunit beta-3 (SCN3B, Q9NY72), plexin-B1 (**PLXNB1**, O43157), netrin receptor (**UNC5C**, O95185). Of significance, many of these proteins are associated with actin remodeling, cell migration and growth. PLXNB1 and UNC5C are directly responsible for axon guidance necessary for neuronal tissue repair after a TBI^35^. The single protein detected in serum, but *not* detected in CSF, is ezrin (EZR, P15311), a protein associated with axon guidance that forms complexes with radixin and moesin part of actin cytoskeleton. Variance in network associations of biochemical pathways, such as axon guidance, can provide useful information when there is disruption in the blood brain barrier or some other dysregulation in protein production and function.

### Assigned metabolites

**Figure 5:**
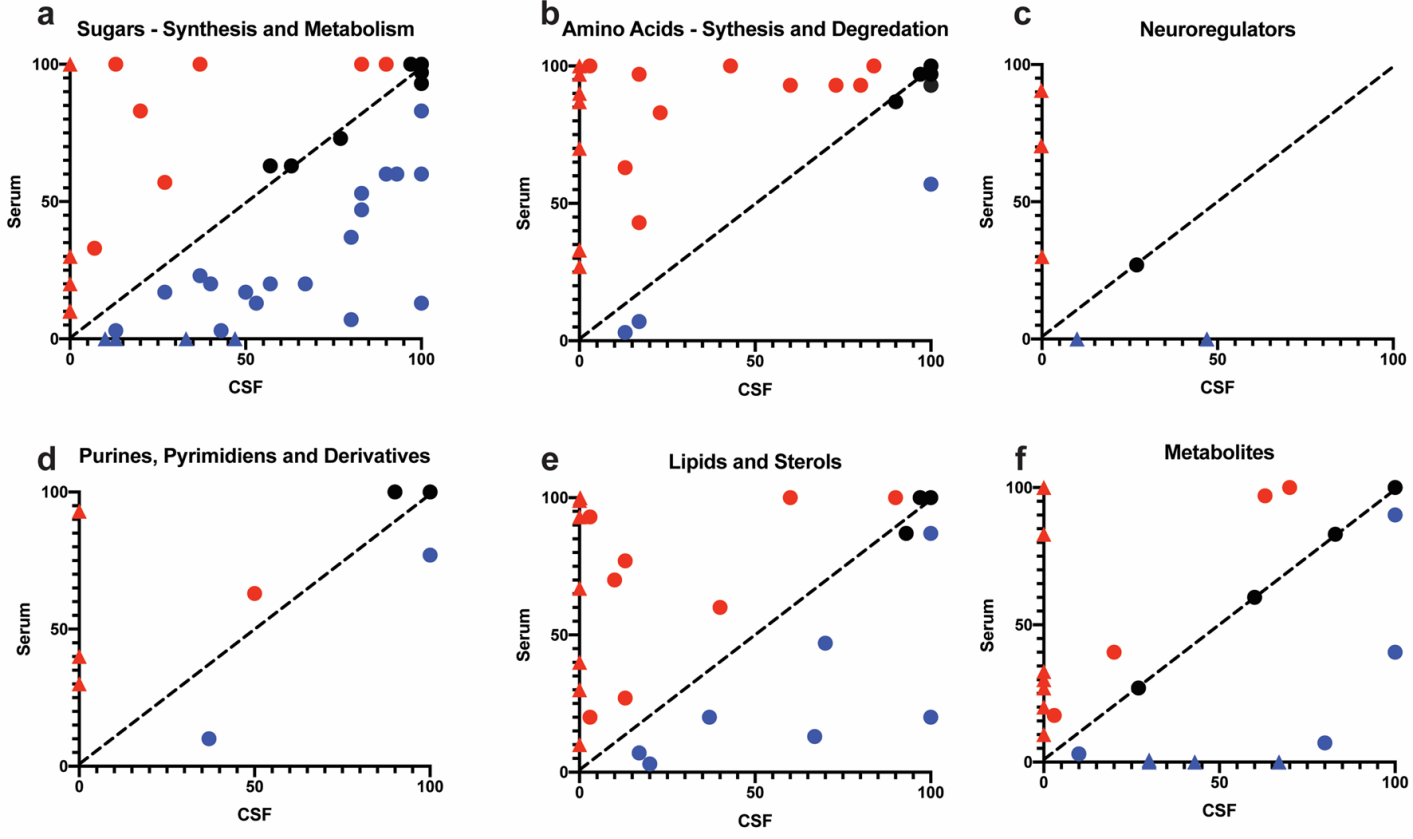
Plots of major classes of identified metabolites, compounds in greater abundance in serum (red circles), exclusively in serum (red triangles), compounds in greater abundance in CSF (blue circles), exclusively in CSF (blue triangles), and compounds in equal (within 5%) abundance (black circles). The black dashed diagonal line represents equal intensities. (**a**) Carbohydrate (sugar) monomers associated with synthesis and metabolism as well as artificial sweeteners. (**b**) The 21 natural amino acids were all detected, amino acid derivatives, and metabolites associated with protein degradation. (**c**) The neuroregulators detected were primarily in serum or CSF. d) Purines, pyrimidines, and their derivatives that make up the nucleosides in DNA and RNA. (**e**) Low molecular weight lipids were detected primarily in serum over CSF. (**f**) Other metabolites that includes urea cycle products, food and drug metabolites.

An investigation of the relative MS intensities of the positively assigned metabolites are plotted based on biochemical class in Fig. 5. In these plots, each point represents a single metabolite and its x,y position is the intensity (0–100) in CSF vs. serum. Points near the diagonal y = x (hashed line in Fig. 5) are from proteins that have nearly equal concentration in CSF and serum. This analysis delineates the metabolic and biochemical needs of each fluid. For example, metabolites associated with sugar synthesis and metabolism are in greater abundance in CSF (Fig. 5a) where amino acid synthesis and metabolism are in greater abundance in serum (Fig. 5b). Circulating serum levels of free amino acids are reflective of protein intake and muscle synthesis. On the other hand, the brain is the most metabolically demanding organ, accounting for 20% of the sugar metabolism. Interestingly, synthetic sugars (e.g., xylitol, sorbitol, and mannitol) were all found in greater abundance in CSF over serum. Of the seven positively identified neuroregulators, (Fig. 5c) we found only one that was detected in both CSF and serum, 5-methoxytryptamine, 5-aminovaleric acid a weak GABA agonist. Serotonin and phenylethylamine were exclusively detected in serum perhaps owing to the lumbar puncture acquisition of CSF. We also note that neuroregulators are concentrated around the brain (Fig. 5d). The two neuroregulators detected solely in CSF were N-acetyl aspartic acid, a modified amino acid found predominately in neurons and the primary metabolite of serotonin, 5-hydroxy-3-indoleacetic acid^36^. We found a greater number of lipids and sterols only in serum; notably palmitoleic acid, linoleic acid, deoxycholic acid, cholic acid, cis-gondoic acid, arachidonic acid, and beta-glycerolphosphate (Fig. 5e). A complete table of the identified metabolites and the relative MS intensities can be found in S5.

#### Gene ontology comparing the proteome and metabolome using gene ontology

Assigning biochemical pathways to lists of proteins and metabolites reveals active/inactive biological functions that can be used to evaluate an individual’s disease state. Here, in our normal population we assign the most common classes of pathways by found by over-representation analysis to our “normal” population (Table 1). These results were derived from using Reactome on our datasets, as we found this to be most illustrative tool of normal biochemical function. Of the off-normal metabolic functions, we found platelet activation and degranulation likely associated with the sample acquisition from the lumbar and venipuncture procedures. The analysis identifies many of the expected normal biochemical pathways including extracellular matrix organization, hemostasis, immune system, metabolism of proteins, and vesicle mediated transport (Table 1). All pathways represented here are associated with soluble proteins/metabolites found in serum and CSF because cells were removed prior to proteomic and metabolomic profiling. Notably, our positively identified metabolites were quite low (as is common) therefore we get limited overlapping coverage with our proteome identified pathways. The complete table of associated pathways from detected proteins (serum S6 and CSF S7) and metabolites (serum S8 and CSF S9) that make up this table can be found in the SI.

**Table 1:**
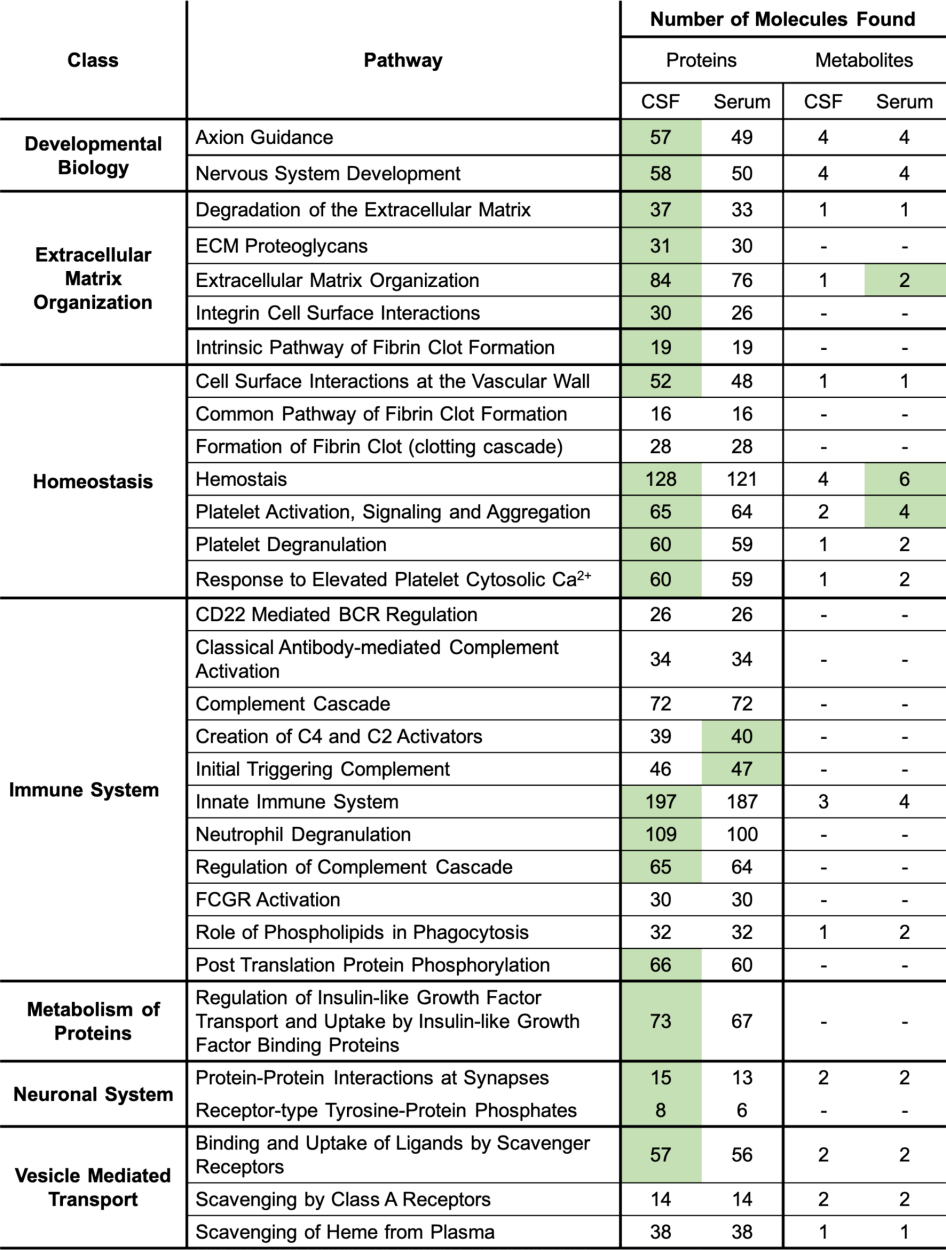
Over-representation analysis of intersecting pathways between the proteome and metabolome of CSF and serum from Reactome.

The general class of pathways (left), pathways found (middle), and the number of either proteins or metabolites found associated with each (right). The media (CSF or serum) with a higher number of identified molecules was highlighted in green.

## Discussion

Development of new therapeutics and diagnostics for neurological insults and injuries requires both the identification of specific biomarkers and associated quantification of normal and abnormal concentrations to determine thresholds for disease detection. The advent of new ‘omic tools has led to innovation in the former however, the later requires examination across different sample types. Further, as our understanding of disease pathophysiology advances, panels of biomarkers have emerged as a more informative diagnostic measurements over single diagnostic targets. Herein, we present proteomic and metabolomic analyses of 60 samples, 30 CSF and 30 serum from individuals with no previous conditions. With this analysis we seek to be as transparent as possible and offer our complete dataset of identified proteins and metabolites (SI) such that other researchers can benefit from a complete control comparison. These proteomic and metabolomic profiles can be used as a control set for other similar ‘omic studies, for comparison to existing datasets, or for thresholding future biomarker discovery efforts—particularly with the common markers implicated in TBI (*e.g*. apolipoproteins). Further, we hope to guide others when selecting the FDR of DIA proteomics in careful consideration of balancing inclusivity with precision. To our knowledge, this is the first comparison of both the metabolome and proteome between CSF and serum. In our demographic analyses we found no significant differences between the metabolome and proteome based on sex. However, we found two significant groups based on age in both the metabolome and proteome that has implications for downstream diagnostic development.

Protein identification was highly dependent on the FDR level. There is a positive relationship between the sensitivity of the procedure (probability of identifying a protein that is in the sample) and the FDR (probability that the identified protein is not in the sample). Since we use protein identification as a pre-processing step to reduce the noise in our measurements, we are willing to be inclusive, meaning that we use a non-negligible FDR. We found that using an FDR of 10% for most analysies provided a good compromise between the two types of errors and is in agreement with other untarged DIA proteomics reports^37^. We had limited ability to identify very low abundance proteins because we chose to not deplete albumin and IgGs due to the error and irreproducibility of these depletion procedures in our hands. Multiple factors can impact the measured intensity and proportional concentration of the detected proteins in serum and CSF. For most of the paper, we assumed that specific protein intensities were not significantly affected by sample matrix effects. The adequacy of this assumption relies on protein concentrations being within the instrument’s linear quantification range. Metabolome identification is intrinsically limited to the sensitivity of BinBase analyses of GC–MS/MS. However, BinBase identification results proved similar to those using other means of identifications^38^. The protocol used for metabolomics normalized to the sum peak height of all structurally annotated compounds of each respective sample matrix.

To contextualize the metabolome and proteome, we analyzed the biochemical pathways using a combination of Reactome, STRING, and KEGG^39–41^. The 216 positively assigned metabolites primary and secondary metabolites were assigned based on KEGG and BinBase. The 813 serum and 932 CSF proteins were assigned to biochemical pathways were assigned between Reactome and STRING. We found only marginal pathway overlap between the metabolome and proteome largely owing to the fact we were looking for extracellular proteins few of which are intrinsically tied to primary/secondary metabolism. We attempted to analyze our data sets using PANTHER and DAVID; however, these tools assigned disease states to our known healthy population largely owing to how their bioinformatic libraries are constructed. While ontological assignments are always challenging owing to limitations of bioinformatic libraries we took care in contextualizing our healthy patent population. In all, we present a comparative cohort of the proteome and metabolome across 30 individuals. These data are a contribution in developing diagnostic/therapeutic targets for injuries/insults to the brain.

## Methods

### General sample information

A total of 30 paired CSF and human serum samples (60 biological samples) from 30 healthy individuals were purchased from PrecisionMed Inc. (Solana Beach, CA). The set comprised 15 males and 15 females ranging in age from 23 to 74. All subjects were Caucasian (European descent) and underwent the Mini International Neuropsychaiactric Interview (M.I.N.I. PLUS) as part of the inclusion. Full details of inclusion/exclusion criteria can be found in Table S10. Blood samples were drawn by venipuncture and collected into sterile tubes; serum was prepared by allowing the blood to sit at room temperature for 15–30 min allowing it to clot. The clotted blood was centrifuged at ~ 1500 × g for 10 min and the serum was aliquoted and stored at − 80 °C. For cerebrospinal fluid collection, the lumbar region of the spine was anesthetized with 2% lidocaine administered subcutaneously. Lumbar puncture was conducted with either a Quincketype or Sprotte side-hole 22G 3.5″ needle. The bevel was placed in line to the Dural cephalocaudal axis to minimize Dural tearing. The needle was placed between the posterior spinous processes of either L5–S1, L4–L5, L3–L4, or L2–L3 and once fluid is seen opening pressure was measured by a sterile manometer. CSF was collected, aliquoted, and stored at − 80 °C. All samples were handled using Eppendorf™ LowBind microcentrifuge tubes and Eppendor Dualfilter T.I.P.S. PCR clean and sterile pipette tips. LCMS grade formic acid, LCMS grade water, and LCMS grade acetonitrile were obtained from Sigma Aldrich. Lipidomic analyses were conducted on an Agilent 6890 GC equipped with Gerstel CIS4 (with dual MPS injector) and a Pegasus III TOF MS. Proteomic analyses were conducted using a Thermo Scientific Fusion Lumos mass spectrometer running in DIA mode.

#### Proteomic sample processing and data acquisition

Sample Preparation Protocol: 100 mL of either CSF or serum were snap frozen in liquid N_2_ in low-bind salinized 1.5 mL microcentrifuge tubes and lyophilized to dryness. Samples were shipped on ice to the UC Davis proteomics core (Davis, CA) for sample processing and MS analysis. These methods where adapted from general protocols of the UC Davis proteomics core^42^.

### Protein digestion

Freeze dried serum and CSF was rehydrated with 5% SDS and 50 mM triethylammonium bicarbonate (TEAB) at pH 7.55. Protein concentration was determined by BCA assay (Fig. S3) and (Pierce), 150 μg of serum was digested on a S-Trap Mini Spin Digestion column and 50 μg of CSF was digested on a S-Trap Micro spin digestion column. Initially, 10 mM dithiothreitol (DTT) was added and incubated at 50 °C for 10 min and rested at room temperature for 10 min. Next, 5 mM iodoacetamide (IAA) was added and incubated at room temperature for 30 min in the dark with a gentle shake. The samples were acidified with 12% phosphoric acid followed by the addition of 2.348 mL of S-Trap buffer (90% methanol, 100 mM TEAB, pH 7.1) and mixed immediately. The entire acidified lysate/St-buffer mix was transferred to the S-Trap spin column and centrifuged at 3000 rcf for 1 min or until all the solution passed through the column. Columns were washed with 600 μL of S-Trap buffer and centrifuged at 2000 rcf until dry. Columns were transferred to a clean elution tube. Trypsin enzyme digest buffer was carefully added (1:25 enzyme: total protein in 121 μL 50 mM TEAB, pH 8.0) to the column and incubated at 37 °C. After the first hour, the trypsin addition step was repeated and the digestion was allowed to continue overnight. Peptide elution steps included 80 μL of 50 mM TEAB (pH 8.0) followed by centrifugation at 1000 rcf for 1 min, 80 μL of 0.5% formic acid followed by centrifugation at 1000 rcf for 1 min, 80 μL of the solution containing 50% acetonitrile and 0.5% formic acid followed by centrifugation at 4000 rcf for 1 min. The final pooled elution was dried in a speed-vacuum. Peptides were resuspended in 0.1% TFA 2% ACN and quantified using Pierce Quantitative Fluorometric Peptide Assay (Thermo Fisher Scientific). Equal portions of all samples, based on the Fluorometric Peptide Assay, were mixed together to make a reference sample to be run six times for chromatogram library runs.

### LC–MS/MS

Peptides were desalted and trapped on a Thermo PepMap trap and separated on an Easy-spray 100 μm × 25 cm C18 column using a Dionex Ultimate 3000 nUPLC at 200 nL/min. Solvent A = 0.1% formic acid, Solvent B = 100% Acetonitrile 0.1% formic acid. Gradient conditions = 2% B to 50% B over 60 min, followed by a 50–99% B in 6 min and then held for 3 min than 99% B to 2% B in 2 min and total run time of 90 min using Thermo Scientific Fusion Lumos mass spectrometer running in DIA mode. Six-gas phase fractionated (GPF) chromatogram library injections were made using staggered 4 Da isolation widows. GPF1 = 400–500 m/z, GPF2 = 500–600 m/z, GPF3 = 600–700 m/z, GPF4 = 700–800 m/z, GPF5 = 800–900 m/z, GPF6 = 900–1000 m/z, mass spectra were acquired using a collision energy of 35, resolution of 30 K, maximum inject time of 54 ms and a AGC target of 50 K.

Each individual sample was run in DIA mode using the same settings as the chromatogram library runs except using staggered isolation windows of 12 Da in the m/z range 400–1000 m/z. DIA data was analyzed using Scaffold DIA v.2.0.0 (Proteome Software, Portland, OR, USA). Raw data files were converted to mzML format using ProteoWizard v.3.0.11748^43^. Total ion chromatograms can be found in S10 for CSF and S11 for Serum.

### Chromatogram library creation

The Reference Spectral Library was created by EncyclopeDIA v.0.9.2. Chromatogram library samples were individually searched against Prosit predicted databases created using Prosit online server (https://www.proteomicsdb.org/prosit/) and converted for ScaffoldDIA using the Encyclopedia tools^44^. The input for the Prosit prediction consisted of Uniprot proteome UP000005640 (*Homo sapiens*) and 114 common laboratory contaminants (https://www.thegpm.org/crap/) with a peptide mass tolerance of 10.0 ppm and a fragment mass tolerance of 10.0 ppm. Variable modifications considered were: oxidation of methionine and carbamidomethyl of cysteine. The digestion enzyme was assumed to be Trypsin with a maximum of 1 missed cleavage site(s) allowed. Only peptides with charges in the range [2‥3] and length in the range [6‥30] were considered. Peptides identified in each search were filtered by Percolator 3.01.nightly-13-655e4c7-dirty) to achieve a maximum FDR of 0.01^45,46^. Individual search results were combined, and peptides were again filtered to an FDR threshold of 0.01 for inclusion in the reference library.

### Spectral library search

Analytic samples were aligned based on retention times and individually searched against the chromatogram library created from the six-gas phase fractionated runs described above with a peptide mass tolerance of 10.0 ppm and a fragment mass tolerance of 10.0 ppm. Variable modifications considered were: Oxidation of methionine and carbamidomethyl of cysteine. The digestion enzyme was assumed to be Trypsin with a maximum of 1 missed cleavage site(s) allowed. Only peptides with charges in the range [2‥3] and length in the range [6‥30] were considered. Peptides identified in each sample were filtered by Percolator (3.01.nightly-13-655e4c7-dirty) to achieve a maximum FDR of 0.01^45–47^. Individual search results were combined and peptide identifications were assigned posterior error probabilities and filtered to an FDR threshold of 0.01 by Percolator (3.01.nightly-13-655e4c7-dirty).

### Quantification and criteria for protein identification

Peptide quantification was performed by EncyclopeDIA v. 0.9.2. For each peptide, the five highest quality fragment ions were selected for quantitation. Proteins that contained similar peptides and could not be differentiated based on MS/MS analysis were grouped to satisfy the principles of parsimony. Proteins with a minimum of 1 or 2 identified peptides and with an FDR of 1.0 or 10.0% were investigated.

### Gene ontology annotation

Proteins were annotated by a combination of STRING^48^ and Reactome^49^. Metabolites were processed in BinBase v 4.0 and KEGG. Comparative analyses were conducted in Reactome, reported pathways had a *p*-value of less than 10^–4^ (S6–S9).

#### Metabolomic sample processing and data acquisition

Sample Preparation Protocol: 500 mL of either CSF or serum were snap frozen in liquid N_2_ in low-bind salinized 1.5 mL microcentrifuge tubes and stored at − 80 °C prior to shipping. Samples were shipped on dry ice to the Westcoast Metabolomics Core (Davis, CA) for sample processing and MS analysis*.*

### Extraction protocol

The following methods were adapted from Feihn et al.^38,50,51^ Samples were thawed at room temperature and vortexed at for 10 s at low speed to homogenize. Samples were aliquoted (30 μL for serum and 50 μL for CSF) and 1 mL ice-cold 3:10 (v/v) MeOH/MTBE + QC mix/CE 22:1 (FAME standard) extraction solvent mixture was added to each aliquot, keeping the samples and extraction solvent on ice during the procedure. Each sample was subsequently vortexed for 10 s (multi-tube vortexer VWR VX-2500). All samples were then centrifuged for 2 min at 14,000 rcf. The organic supernatants were separated into two separate 450 mL aliquots, one for primary analysis. 75 µL of the remaining organic phases was transferred to 50 mL conical tube to generate pooled samples of either CSF or serum. The remaining organic phases were separated and kept at − 20 °C as backups. All primary and pooled samples were dried *in vauco* by a speed vacuum concentration system (Labcono Centrivap cold trap). Serum samples were further cleaned by resuspended in 500 μL 50:50 (v/v) ACN:H_2_O degassed with argon. The samples were centrifuged for 2 min at 14,000 ref and supernatants (475 μL) were transferred to new Eppendorf tubes.

### Sample derivatization and GC–MS sample setup

To remove very hydrophobic lipids, serum and CSF samples were resuspended in 500 μL 50:50 (v/v) ACN:H_2_O degassed with argon. The samples were centrifuged for 2 min at 14,000 ref and supernatants (475 μL) were transferred to new Eppendorf tubes.

### GC–MS conditions and settings

The Agilent 6890 GC is equipped with a Gerstel automatic liner exchange system (ALEX) that includes a multipurpose sample (MPS2) dual rail, and a Gerstel CIS cold injection system (Gerstel, Muehlheim, Germany) with temperature program as follows: 50 °C to 275 °C final temperature at a rate of 12 °C/s and held for 3 min. Injection volume is 0.5 μL with 10 μL/s injection speed on a spitless injector with purge time of 25 s. For quality assurance, the liner (Gerstel #011,711-010-00) was changed after every 10 samples, (using the Maestro1 Gerstel software vs. 1.1.4.18). Before and after each injection, the 10 μL injection syringe is washed three times with 10 μL ethyl acetate.

A 30 m long, 0.25 mm i.d. Rtx-5Sil MS column (0.25 μm 95%, dimethyl 5%, diphenyl polysiloxane film) with additional 10-m integrated guard column (Restek, Bellefonte PA). 99.9999% pure Helium with built-in purifier (Airgas, Radnor PA) is set at constant flow of 1 mL/minute. The oven temperature is held constant at 50 °C for 1 min and then ramped at 20 °C/minute to 330 °C where it was held constant for 5 min. A Leco Pegasus IV time of flight mass spectrometer is controlled by the Leco ChromaTOF software versus 2.32 (St. Joseph, MI). The transfer line temperature between gas chromatograph and mass spectrometer is set to 280 °C. Electron impact ionization at 70 V is employed with an ion source temperature of 250 °C. Acquisition rate is 17 spectra/second, with a scan mass range of 85–500 Da.

### Quantification and criteria for metabolite identification

Raw data files are preprocessed directly after data acquisition and stored as ChromaTOF-specific *.peg files, as generic *.txt result files and additionally as generic ANDI MS *.cdf files. Preprocessing in ChromaTOF vs. 2.32 (Leco) is conducted without smoothing, a baseline subtraction is performed along with automatic spectral deconvolution and peak detection with S/N of 5:1. Apex masses and a corresponding *.txt output with the absolute intestines are exported for further processed by a filtering algorithm implemented in the metabolomics BinBase v 4.0 database. Details on the BinBase (https://code.google.com/p/binbase/) algorithm was developed by Feihn et al.^27,38^ Spectra are automatically aligned to the QC mix within BinBase and samples were normalized to the sum peak heights of all structurally identified compounds—to correct for matrix effects of serum and CSF. Known metabolites are assigned to their respective PubChem, KEGG, and InChi Key.

### Statistical methods

#### Imputation of missing values

Missing values of intensity were imputed for all proteins detected in at least one of the samples. We assumed intensities followed a lognormal distribution with a detection threshold varying between proteins and matrices. We fitted each truncated distribution to find the mean and variance, approximating the threshold as equal to the smallest of the observed intensities. When less than 3 intensity values were observed for a protein, we assumed a mean and standard deviation equal to the average across the fitted distributions (mean = 4, std = 1). Unobserved values were imputed by generating random numbers from the censored part of the derived distributions. For metabolomics data, BinBase imputed intensity values when a metabolite was detected in at least one of the samples^38^. Imputed values were chosen such that the intensity was within the range of the unexplained noise in each mass spectrum.

#### Statistical analyses

All analyses were performed after imputation of missing values using R 3.6.3. To assess whether intensities differed in terms of sample type (CSF vs. serum), we first performed a Principal Component Analysis on each of the proteomics and metabolomics datasets. We then plotted the samples on the first two principal components to observe differences in this simplified space. We identified single proteins that significantly differed in relative intensity between sample types by performing paired sample t-tests on the logarithm of the concentrations, adjusting the p-values for multiplicity of testing using Benjamini–Hochberg method. We further computed the average fold-change in intensity across participants to assess the clinical significance of findings.

We evaluated whether groups of participants appeared to have similar proteomics profiles. For this purpose, we performed a Principal Component Analysis on each of the two sample types. We then selected components for which the explained variance appeared to be signal over noise (before the elbow of the plot of explained variance over component number). Using these components, we performed hierarchical clustering with Ward’s distance and plotted the obtained tree. We then visually assessed whether there appeared to be groups with similar profiles. Groups of participants were then compared as a function of age and sex using t-tests and chi-squared tests, respectively. The above was repeated on metabolomics data.

## Supplementary Information

Supplementary Information.
