# Supplementary material for: Methods to capture proteomic and metabolomic signatures from cerebrospinal fluid and serum of healthy individuals
Source: Sci Rep. 2022 Aug 3;12:13339. doi: 10.1038/s41598-022-16598-1 (PMC9349260; doi:10.1038/s41598-022-16598-1)
Supplement: Supplementary file 1 — Supplementary Information. [file 41598_2022_16598_MOESM1_ESM.docx]

Supporting Information for:

**Methods to Capture Proteomic and Metabolomic Signatures from Cerebrospinal Fluid and Serum of Healthy Individuals**

# Laura M. Lilley^a^; Steven Sanche^a^; Shepard C. Moore^a^; Michelle R. Salemi^b^; Dung Vu^a^; Srinivas, Iyer^a^; Nicolas W. Hengartner^a^; Harshini Mukundan^a*^

^a^ Los Alamos National Laboratory, Los Alamos, New Mexico 87545, USA

^b^ Genome Center, Proteomics Core Facility, University of California, Davis, CA 95616, USA

Email: [harshini@lanl.gov](mailto:harshini@lanl.gov)

[**Table S1:** Serum proteins detected by FDR, UNIPROT identifiers listed. 2](#_Toc82097011)

[**Table S2:** CSF proteins detected by FDR, UNIPTOT identifiers listed. 6](#_Toc82097012)

[**Table S3:** Proteome clustering based on age, proteins reported by UNIPROT identifiers. 10](#_Toc82097013)

[**Table S4:** Metabolome clustering based on age, proteins reported by BinBase identifier numbers and Binbase name. 26](#_Toc82097014)

[**Table S5:** Table of identified metabolites and the associated MS percentages in CSF and Serum reported by BinBase name, BinBase identifier, and KEGG identifier. 38](#_Toc82097015)

[**Table S6:** Reactome pathways found from serum proteins detected at the 10% FDR level. Entities found are the entered proteins, total entities are the reported proteins in the pathway. Pathways were cut off at p = 0.05. 54](#_Toc82097016)

[**Table S7:** Reactome pathways found from CSF proteins detected at the 10% FDR level. Entities found are the entered proteins, total entities are the reported proteins in the pathway. Pathways were cut off at p = 0.05. 59](#_Toc82097017)

[**Table S8:** Reactome pathways found from serum metabolites detected at the 10% FDR level. Entities found are the entered proteins, total entities are the reported proteins in the pathway. Pathways were cut off at p = 0.05. 66](#_Toc82097018)

[**Table S9:** Reactome pathways found from CSF metabolites detected at the 10% FDR level. Entities found are the entered proteins, total entities are the reported proteins in the pathway. Pathways were cut off at p = 0.05. 70](#_Toc82097019)

[**Figure S1:** Total ion chromatograms from proteomic analysis of the 30 CSF samples. 73](#_Toc82097020)

[**Figure S2:** Total ion chromatograms from proteomic analysis of the 30 serum samples. 74](#_Toc82097021)

[**Table S10:** PrecisionMed sample banking Inclusion/Exclusion Criteria. To qualify subjects must answer yes to all inclusion criteria and no to all exclusion criteria. 75](#_Toc82097022)

**Figure S3** Protein concentration in each sample determined by BCA assay………………………....….76

**Table S1:** Serum proteins detected by FDR, UNIPROT identifiers listed.

| **1% FDR** | | | **5% FDR** | **10% FDR** |
| --- | --- | --- | --- | --- |
| A0A075B6H9  A0A075B6I0  A0A075B6J9  A0A075B6K2  A0A075B6K4  A0A075B6K5  A0A075B6Q5  A0A075B6R2  A0A075B6S5  A0A075B6S6  A0A087WSX0  A0A087WSY6  A0A087WSZ0  A0A087WW87  A0A0A0MRZ8  A0A0A0MS14  A0A0A0MS15  A0A0B4J1U3  A0A0B4J1U7  A0A0B4J1V0  A0A0B4J1V2  A0A0B4J1V6  A0A0B4J1X8  A0A0B4J1Y8  A0A0B4J1Y9  A0A0B4J2D9  A0A0B4J2H0  A0A0C4DH24  A0A0C4DH25  A0A0C4DH29  A0A0C4DH31  A0A0C4DH32  A0A0C4DH33  A0A0C4DH34  A0A0C4DH38  A0A0C4DH39  A0A0C4DH43  A0A0C4DH55  A0A0C4DH68  A0A0G2JMI3  A0A0J9YX35  A0A0J9YXX1  A0M8Q6  B9A064  C9JN71  O00187  O00241  O00391  O00461  O00462  O00533  O14498  O14594  O14646  O14786  O14791  O15031  O15240  O15389  O15394  O43300  O43505  O43615  O43866  O75078  O75144  O75223  O75326  O75636  O75882  O94769  O94910  O94985  O95445  O95502  O95633  O95967  P00338  P00450  P00488  P00558  P00734  P00736  P00738  P00739  P00740  P00742  P00746  P00747  P00748  P00751  P00760  P00766  P00915  P01008  P01009  P01011  P01019  P01023  P01024  P01031  P01033  P01034  P01042  P01344  P01591  P01593  P01597  P01599  P01601  P01602  P01619  P01700  P01701  P01703  P01704  P01718  P01721  P01743  P01780  P01817  P01833  P01834  P01857  P01859  P01860  P01861  P01871  P01876  P01877  P01880  P01891  P01893  P02042  P02452  P02461  P02533  P02538  P02647  P02649  P02652  P02654  P02655  P02656  P02671  P02675  P02679  P02741  P02743  P02745  P02746  P02747  P02748  P02749  P02750  P02751  P02753  P02760  P02763  P02765  P02766  P02768  P02769  P02774  P02786  P02787  P02788  P02790  P02792  P03951  P03952  P04003  P04004  P04040  P04070  P04114  P04180  P04196  P04217  P04259  P04264  P04275  P04278  P04406  P04430  P05060  P05067  P05090  P05154  P05155  P05156  P05160  P05362  P05413  P05452  P05543  P05546  P05787 | P06276  P06312  P06331  P06396  P06681  P06702  P06727  P07225  P07333  P07339  P07355  P07357  P07358  P07360  P07451  P07477  P07602  P07711  P07858  P07900  P07942  P07996  P07998  P08123  P08138  P08174  P08185  P08253  P08294  P08519  P08571  P08603  P08637  P08697  P08709  P08779  P09172  P09417  P09486  P09668  P09871  P09972  P0C0L4  P0C0L5  P0CG47  P0DJI8  P0DOY2  P0DP01  P0DP04  P10253  P10412  P10599  P10619  P10643  P10645  P10721  P10909  P11021  P11047  P12035  P12109  P12111  P12259  P12830  P13473  P13591  P13598  P13611  P13645  P13647  P13671  P13727  P13796  P14151  P14314  P14543  P14618  P14625  P14923  P15144  P15151  P15169  P15291  P15586  P15814  P15924  P16070  P16112  P16870  P17174  P17936  P18065  P18428  P19021  P19320  P19652  P19823  P19827  P19909  P20742  P20774  P20851  P20933  P22061  P22105  P22352  P22692  P22792  P22891  P23083  P23142  P23470  P23471  P25311  P26927  P26992  P27169  P27487  P27797  P27918  P29622  P30043  P31150  P32119  P33151  P33908  P35052  P35443  P35527  P35542  P35555  P35858  P35908  P36222  P36955  P36980  P39060  P40925  P41217  P41222  P42785  P43121  P43146  P43251  P43652  P47972  P48058  P48740  P49065  P49641  P49747  P49908  P51693  P51884  P52758  P54289  P54756  P54802  P55058  P55083  P55268  P55285  P55290  P60174  P60709  P61626  P61769  P62736  P62937  P68871  P69905  P78324  P78417  P78509  P80108  P80188  P98095  P98160  Q02413  Q02809  Q03591  Q04695  Q04756  Q06033  Q06481  Q06830  Q07954  Q08174  Q08380  Q08554  Q08629  Q10588  Q12805  Q12860  Q12907  Q12913  Q12931  Q13217 | Q13201  Q76LX8  Q9Y2K3  A0A0C4DH67  P00883  Q13449  Q13740  Q13822  Q13835  Q13867  Q14118  Q14126  Q14515  Q14520  Q14624  Q14697  Q14982  Q14CN4  Q15063  Q15113  Q15166  Q15223  Q15517  Q15582  Q15782  Q15818  Q15828  Q16270  Q16610  Q24JP5  Q53EL9  Q562R1  Q5SRE5  Q6EMK4  Q6IE36  Q6UWP8  Q6UX71  Q6UXB8  Q6UY11  Q6YHK3  Q6ZMI3  Q7LFX5  Q7Z5L0  Q7Z794  Q7Z7G0  Q7Z7M0  Q86SF2  Q86UN3  Q86UX2  Q86VB7  Q86YZ3  Q8IV08  Q8IVF6  Q8IZJ3  Q8N1N4  Q8NBP7  Q8NCL4  Q8NCX0  Q8NFP4  Q8NFY4  Q8NFZ4  Q8NFZ8  Q8TCZ2  Q8TDD5  Q8TEU8  Q8WWM7  Q92496  Q92820  Q92823  Q92859  Q92876  Q92954  Q96BZ4  Q96GW7  Q96IY4  Q96KG7  Q96KN2  Q96PD5  Q96PX8  Q96S96  Q99435  Q99453  Q99784  Q9BQ16  Q9BRK5  Q9BUN1  Q9BWP8  Q9BYH1  Q9BZR6  Q9C0A0  Q9H2X0  Q9H3G5  Q9H4E7  Q9H4G4  Q9H7Y0  Q9H8L6  Q9HCB6  Q9HDC9  Q9NPH3  Q9NQ79  Q9NR34  Q9NRJ7  Q9NRN5  Q9NSB2  Q9NYQ8  Q9NZP8  Q9P2E7  Q9P2S2  Q9P2S5  Q9UBP4  Q9UBR2  Q9UBX1  Q9UEW3  Q9UGM5  Q9UHC6  Q9UHG3  Q9UJJ9  Q9UK55  Q9ULB1  Q9ULP0  Q9UMF0  Q9UN36  Q9UNN8  Q9UNW1  Q9Y376  Q9Y4C0  Q9Y5R5  Q9Y5Y7  Q9Y6N8  Q9Y6R7  A0A075B6H7  A0A075B6S9  A0A075B7B8  A0A075B7D0  A0A075B7D4  A0A075B7D8  A0A087WYE8  A0A0B4J2B8  A0A0C4DH35  A0A0C4DH36  A0A0G2JRQ6  A0A0J9YW62  A0A0J9YY99  A0A075B6I9  O14490  P02776  P11597  P14780  P22735  P40197  P55056  Q13103  Q15485  Q15848  Q86XP3  P02775  P05062  P07359  Q13093  Q13790  Q9BXR6  P28799  P49913 | A1L4H1  O00264  O00468  O00584  O14514  O15197  O60243  O60245  O60279  O60462  O75787  O76009  O94856  O95428  O95490  P00390  P00441  P00505  P02689  P04066  P04216  P06733  P07195  P07585  P07686  P07737  P08572  P09211  P10451  P10586  P11142  P11279  P11362  P11717  P12318  P13521  P13929  P14621  P15259  P19022  P21810  P22004  P22304  P22748  P22897  P23284  P23468  P23515  P29279  P29762  P30041  P34096  P35237  P35442  P38571  P39059  P40189  P42658  P48745  P50750  P50897  P51888  P52797  P52799  P54764  P55291  P60983  P63010  Q01459  Q01995  Q02246  Q04760  Q06828  Q08345  Q13228  Q13332  Q13421  Q13508  Q14004  Q14112  Q14165  Q14315  Q14393  Q14766  Q14767  Q16653  Q16706  Q16849  Q31612  Q495W5  Q5JRA6  Q5JST6  Q5KU26  Q6MZW2  Q6NW40  Q6UX73  Q7Z3B1  Q86UD1  Q8IUC8  Q8IUK5  Q8IUX7  Q8IWV2  Q8IZP7  Q8N126  Q8N2S1  Q8N3H0  Q8N3J6  Q8N3T6  Q8N475  Q8NBJ4  Q8NES3  Q8NHP8  Q8TAG5  Q8TDQ0  Q8TEA8  Q8TER0  Q8WVQ1  Q8WXD2  Q92752  Q92765  Q969P0  Q96CX2  Q96FE7  Q96ID5  Q96RR4  Q99497  Q99523  Q99574  Q99715  Q99983  Q9BQT9  Q9BRK3  Q9BTY2  Q9BXJ0  Q9BY67  Q9GZX9  Q9H741  Q9HAR2  Q9HBT6  Q9HBW1  Q9NPC4  Q9NPD7  Q9NT99  Q9NTU7  Q9NX62  Q9NZ08  Q9P121  Q9UBQ6  Q9UBX5  Q9UF11  Q9UHG2  Q9UHL4  Q9UM22  Q9UM47  Q9UN70  Q9UPU3  Q9UQ52  Q9UQM7  Q9Y279  Q9Y2I2  Q9Y2T3  Q9Y5I4  Q9Y6N7  Q8IVF4  Q8NBS9  Q9UIB8  P15311 | A0A0A0MT36  O00115  O00339  O00451  O14773  O75711  O94779  O94919  O95185  O95206  O95897  P02144  P03973  P06865  P07093  P07108  P08195  P08758  P0DP58  P15289  P16035  P17050  P17900  P20062  P21246  P23435  P24043  P24592  P24593  P30086  P30101  P32004  P34059  P48723  P50895  P55283  P60033  P61916  Q01469  Q02487  Q03167  Q08431  Q10469  Q10471  Q12841  Q13214  Q13510  Q13642  Q15303  Q15375  Q15904  Q16769  Q5VU97  Q6FHJ7  Q6UXD5  Q8NFT8  Q8WZA1  Q92520  Q92911  Q969T9  Q96B86  Q99969  Q9BU40  Q9H2A7  Q9H2E6  Q9HAT2  Q9NPR2  Q9NPY3  Q9NS85  Q9NS98  Q9NY93  Q9NY97  Q9NZ53  Q9UBG0  Q9UBX7  Q9ULF5  Q9Y240  Q9Y4L1  Q9Y617  Q9Y646  O95970  P29972  O60568  P17931  Q6UY14  Q9Y6Z7 |

**Table S2:** CSF proteins detected by FDR, UNIPTOT identifiers listed.

| **1% FDR** | | | | | **5% FDR** | **10% FDR** |
| --- | --- | --- | --- | --- | --- | --- |
| A0A075B6H9  A0A075B6I0  A0A075B6J9  A0A075B6K2  A0A075B6K4  A0A075B6K5  A0A075B6Q5  A0A075B6R2  A0A075B6S5  A0A075B6S6  A0A087WSX0  A0A087WSY6  A0A087WSZ0  A0A087WW87  A0A0A0MRZ8  A0A0A0MS14  A0A0A0MS15  A0A0B4J1U3  A0A0B4J1U7  A0A0B4J1V0  A0A0B4J1V2  A0A0B4J1V6  A0A0B4J1X8  A0A0B  A0A0B4J1Y9  A0A0B4J2D9  A0A0B4J2H0  A0A0C4DH24  A0A0C4DH25  A0A0C4DH29  A0A0C4DH31  A0A0C4DH32  A0A0C4DH33  A0A0C4DH34  A0A0C4DH38  A0A0C4DH39  A0A0C4DH43  A0A0C4DH55  A0A0C4DH68  A0A0G2JMI3  A0A0J9YX35  A0A0J9YXX1  A0M8Q6  B9A064  C9JN71  O00187  O00241  O00391  O00461  O00462  O00533  O14498  O14594  O14646  O14786  O14791  O15031  O15240  O15389  O15394  O43300  O43505  O43615  O43866  O75078  O75144  O75223  O75326  O75636  O75882  O94769  O94910  O94985  O95445  O95502  O95633  O95967  P00338  P00450  P00488  P00558  P00734  P00736  P00738  P00739  P00740  P00742  P00746  P00747  P00748  P00751  P00760  P00766  P00915  P01008  P01009  P01011  P01019  P01023  P01024  P01031  P01033  P01034  P01042  P01344  P01591  P01593  P01597  P01599  P01601  P01602  P01619  P01700  P01701  P01703  P01704  P01718  P01721  P01743  P01780  P01817  P01833  P01834  P01857  P01859  P01860  P01861  P01871  P01876  P01877  P01880  P01891  P01893  P02042  P02452  P02461  P02533  P02538  P02647  P02649  P02652  P02654  P02655  P02656  P02671  P02675  P02679  P02741  P02743  P02745  P02746  P02747  P02748  P02749  P02750  P02751  P02753  P02760  P02763  P02765  P02766  P02768  P02769  P02774  P02786  P02787  P02788  P02790  P02792  P03951  P03952  P04003  P04004  P04040  P04070  P04114  P04180  P04196  P04217  P04259  P04264  P04275  P04278  P04406  P04430  P05060  P05067  P05090  P05154  P05155  P05156  P05160  P05362  P05413  P05452  P05543  P05546  P05787  P06276  P06312  P06331  P06396  P06681  P06702  P06727  P07225  P07333  P07339 | P07355  P07357  P07358  P07360  P07451  P07477  P07602  P07711  P07858  P07900  P07942  P07996  P07998  P08123  P08138  P08174  P08185  P08253  P08294  P08519  P08571  P08603  P08637  P08697  P08709  P08779  P09172  P09417  P09486  P09668  P09871  P09972  P0C0L4  P0C0L5  P0CG47  P0DJI8  P0DOY2  P0DP01  P0DP04  P10253  P10412  P10599  P10619  P10643  P10645  P10721  P10909  P11021  P11047  P12035  P12109  P12111  P12259  P12830  P13473  P13591  P13598  P13611  P13645  P13647  P13671  P13727  P13796  P14151  P14314  P14543  P14618  P14625  P14923  P15144  P15151  P15169  P15291  P15586  P15814  P15924  P16070  P16112  P16870  P17174  P17936  P18065  P18428  P19021  P19320  P19652  P19823  P19827  P19909  P20742  P20774  P20851  P20933  P22061  P22105  P22352  P22692  P22792  P22891  P23083  P23142  P23470  P23471  P25311  P26927  P26992  P27169  P27487  P27797  P27918  P29622  P30043  P31150  P32119  P33151  P33908  P35052  P35443  P35527  P35542  P35555  P35858  P35908  P36222  P36955  P36980  P39060  P40925  P41217  P41222  P42785  P43121  P43146  P43251  P43652  P47972  P48058  P48740  P49065  P49641  P49747  P49908  P51693  P51884  P52758  P54289  P54756  P54802  P55058  P55083  P55268  P55285  P55290  P60174  P60709  P61626  P61769  P62736  P62937  P68871  P69905  P78324  P78417  P78509  P80108  P80188  P98095  P98160  Q02413  Q02809  Q03591  Q04695  Q04756  Q06033  Q06481  Q06830  Q07954  Q08174  Q08380  Q08554  Q08629  Q10588  Q12805  Q12860  Q12907  Q12913  Q12931  Q13217  Q13449  Q13740  Q13822  Q13835  Q13867  Q14118  Q14126  Q14515  Q14520  Q14624  Q14697  Q14982  Q14CN4  Q15063  Q15113  Q15166  Q15223  Q15517  Q15582  Q15782 | Q15818  Q15828  Q16270  Q16610  Q24JP5  Q53EL9  Q562R1  Q5SRE5  Q6EMK4  Q6IE36  Q6UWP8  Q6UX71  Q6UXB8  Q6UY11  Q6YHK3  Q6ZMI3  Q7LFX5  Q7Z5L0  Q7Z794  Q7Z7G0  Q7Z7M0  Q86SF2  Q86UN3  Q86UX2  Q86VB7  Q86YZ3  Q8IV08  Q8IVF6  Q8IZJ3  Q8N1N4  Q8NBP7  Q8NCL4  Q8NCX0  Q8NFP4  Q8NFY4  Q8NFZ4  Q8NFZ8  Q8TCZ2  Q8TDD5  Q8TEU8  Q8WWM7  Q92496  Q92820  Q92823  Q92859  Q92876  Q92954  Q96BZ4  Q96GW7  Q96IY4  Q96KG7  Q96KN2  Q96PD5  Q96PX8  Q96S96  Q99435  Q99453  Q99784  Q9BQ16  Q9BRK5  Q9BUN1  Q9BWP8  Q9BYH1  Q9BZR6  Q9C0A0  Q9H2X0  Q9H3G5  Q9H4E7  Q9H4G4  Q9H7Y0  Q9H8L6  Q9HCB6  Q9HDC9  Q9NPH3  Q9NQ79  Q9NR34  Q9NRJ7  Q9NRN5  Q9NSB2  Q9NYQ8  Q9NZP8  Q9P2E7  Q9P2S2  Q9P2S5  Q9UBP4  Q9UBR2  Q9UBX1  Q9UEW3  Q9UGM5  Q9UHC6  Q9UHG3  Q9UJJ9  Q9UK55  Q9ULB1  Q9ULP0  Q9UMF0  Q9UN36  Q9UNN8  Q9UNW1  Q9Y376  Q9Y4C0  Q9Y5R5  Q9Y5Y7  Q9Y6N8  Q9Y6R7  A0A075B6H7  A0A075B6S9  A0A075B7B8  A0A075B7D0  A0A075B7D4  A0A075B7D8  A0A087WYE8  A0A0B4J2B8  A0A0C4DH35  A0A0C4DH36  A0A0G2JRQ6  A0A0J9YW62  A0A0J9YY99  A1L4H1  O00264  O00468  O00584  O14514  O15197  O60243  O60245  O60279  O60462  O75787  O76009  O94856  O95428  O95490  P00390  P00441  P00505  P02689  P04066  P04216  P06733  P07195  P07585  P07686  P07737  P08572  P09211  P10451  P10586  P11142  P11279  P11362  P11717  P12318  P13521  P13929  P14621  P15259  P19022  P21810  P22004  P22304  P22748  P22897  P23284  P23468  P23515  P29279  P29762  P30041  P34096  P35237  P35442  P38571  P39059  P40189  P42658  P48745  P50750  P50897  P51888  P52797  P52799  P54764  P55291  P60983  P63010  Q01459  Q01995  Q02246  Q04760  Q06828  Q08345  Q13228  Q13332  Q13421  Q13508  Q14004  Q14112  Q14165  Q14315  Q14393  Q14766  Q14767  Q16653  Q16706  Q16849  Q31612  Q495W5 | Q5JRA6  Q5JST6  Q5KU26  Q6MZW2  Q6NW40  Q6UX73  Q7Z3B1  Q86UD1  Q8IUC8  Q8IUK5  Q8IUX7  Q8IWV2  Q8IZP7  Q8N126  Q8N2S1  Q8N3H0  Q8N3J6  Q8N3T6  Q8N475  Q8NBJ4  Q8NES3  Q8NHP8  Q8TAG5  Q8TDQ0  Q8TEA8  Q8TER0  Q8WVQ1  Q8WXD2  Q92752  Q92765  Q969P0  Q96CX2  Q96FE7  Q96ID5  Q96RR4  Q99497  Q99523  Q99574  Q99715  Q99983  Q9BQT9  Q9BRK3  Q9BTY2  Q9BXJ0  Q9BY67  Q9GZX9  Q9H741  Q9HAR2  Q9HBT6  Q9HBW1  Q9NPC4  Q9NPD7  Q9NT99  Q9NTU7  Q9NX62  Q9NZ08  Q9P121  Q9UBQ6  Q9UBX5  Q9UF11  Q9UHG2  Q9UHL4  Q9UM22  Q9UM47  Q9UN70  Q9UPU3  Q9UQ52  Q9UQM7  Q9Y279  Q9Y2I2  Q9Y2T3  Q9Y5I4  Q9Y6N7  A0A0A0MT36  O00115  O00339  O00451  O14773  O75711  O94779  O94919  O95185  O95206  O95897  P02144  P03973  P06865  P07093  P07108  P08195  P08758  P0DP58  P15289  P16035  P17050  P17900  P20062  P21246  P23435  P24043  P24592  P24593  P30086  P30101  P32004  P34059  P48723  P50895  P55283  P60033  P61916  Q01469  Q02487  Q03167  Q08431  Q10469  Q10471  Q12841  Q13214  Q13510  Q13642  Q15303  Q15375  Q15904  Q16769  Q5VU97  Q6FHJ7  Q6UXD5  Q8NFT8  Q8WZA1  Q92520  Q92911  Q969T9  Q96B86  Q99969  Q9BU40  Q9H2A7  Q9H2E6  Q9HAT2  Q9NPR2  Q9NPY3  Q9NS85  Q9NS98  Q9NY93  Q9NY97  Q9NZ53  Q9UBG0  Q9UBX7  Q9ULF5  Q9Y240  Q9Y4L1  Q9Y617  Q9Y646  A0A0C4DH67  A6NGN9  A6NLU5  O14793  O14917  O15466  O43157  O43291  O43405  O43529  O43556  O60241  O60888  O75493  O75503  O75509  O75828  O75962  O94772  O95965  P01210  P04075  P04156  P04179  P04745  P05089  P05937  P06744  P07237  P08493  P08670  P09104  P09382  P09603  P09619  P13987  P14415  P15328  P21333  P21802  P22607  P23582  P25774  P26038  P26572  P29218  P29401  P29966  P30530  P31944  P35080  P35590  P36871  P43234  P45877 | P48061  P49257  P54803  P57087  P61981  P68363  P69849  P69891  P78539  P80723  Q02818  Q06418  Q07092  Q10472  Q13231  Q13554  Q14C87  Q16288  Q16568  Q16620  Q16674  Q5BIV9  Q6UVK1  Q6UW01  Q6UXK2  Q6ZRP7  Q86VZ4  Q86Y38  Q8IVN8  Q8IW52  Q8IZA0  Q8N6C5  Q8NBI6  Q8NCW5  Q8WUJ3  Q8WY21  Q92563  Q92673  Q92743  Q92932  Q93091  Q95604  Q969H8  Q96AP7  Q96FE5  Q96JF0  Q96RS0  Q99538  Q9BRA2  Q9BX67  Q9H3T3  Q9HC56  Q9HCU0  Q9NY47  Q9NY72  Q9NYX4  Q9P0K1  Q9UJA9  Q9UKU6  Q9ULL4  Q9UP79  Q9UQ35  Q9Y5F6  Q9Y696 | A0A075B6I9  O14490  P02776  P11597  P14780  P22735  P40197  P55056  Q13103  Q15485  Q15848  Q86XP3  Q8IVF4  Q8NBS9  O95970  P29972  O15354  P41271  Q14574  Q6UXH9  Q99519  Q9C0C4  Q9NY15 | P02775  P05062  P07359  Q13093  Q13790  Q9BXR6  Q9UIB8  O60568  A0A0B4J1X5  P24821  P30740  Q53RD9  Q9BS26 |

**Table S3:** Proteome clustering based on age, proteins reported by UNIPROT identifiers.

| UNIPROT | Adjusted p-value | Mean Young | Mean Old |
| --- | --- | --- | --- |
| P40925  P05156  Q15818  Q9BZR6  P02649  Q6MZW2  Q9ULB1  P07358  Q92823  Q96GW7  Q9Y4C0  P08697  O43505  O00533  O95502  P00505  P13671  P17174  Q6UXD5  Q7Z7M0  Q8IV08  Q96B86  P51693  Q8WVQ1  Q9BYH1  O94985  P32004  Q9C0A0  P27169  P01700  P07195  O14594  P18428  Q86UN3  Q99435  P12109  P35237  P54289  Q92859  O00451  Q9NS85  P17900  P29622  P02760  P01877  P78509  P07093  P07360  Q9P0K1  P36980  Q9NT99  P02679  O14514  P23515  P02675  P60174  P06681  Q16288  Q9NX62  Q8WXD2  Q9NQ79  Q9P121  P02748  Q99574  Q9UBQ6  O15394  P05067  P00734  O75326  P14618  O94856  Q14697  P43146  O75962  P02750  P13591  Q12860  P54764  P55290  P48058  O60241  P01034  Q14624  P01591  Q96PD5  Q9Y2T3  P78324  Q14C87  Q7Z3B1  P01042  Q9ULF5  P07602  O75493  Q92520  Q96FE5  Q9UHC6  Q06481  P54756  O94910  O75509  P09104  Q8NFT8  P19021  P47972  Q9Y5F6  P04217  Q24JP5  Q9UQM7  P00450  P02671  Q9UBP4  P04196  P10586  A0A0J9YX35  Q08431  P01031  P30086  P19827  A0A087WSY6  P14314  Q14520  Q9UQ52  P01019  P35858  O95206  P08185  A0A0A0MS15  P01008  A0A075B7D4  Q12907  P23471  A0A0C4DH38  P05154  Q8NFZ8  Q96PX8  A0A0C4DH68  Q9UMF0  Q96KN2  Q14982  P00441  A0A0B4J2D9  A0A0C4DH36  Q9HAR2  A0A075B6S9  A0A075B6K5  Q8IW52  Q9NY93  Q13332  P10909  Q92954  P02774  P04075  P30530  Q08174  Q9Y5I4  P04216  P00740  Q9NTU7  Q9H3T3  P31150  O15240  P01780  Q92876  P05362  Q9Y2I2  P04156  P15169  O60245  Q9UM22  P04180  P02749  P01721  O00468  P41222  Q9UJA9  P05543  P55291  Q8N3J6  P15151  P19022  P11279  P09972  A6NLU5  P14621  Q14515  P00748  O75787  O75636  P33908  P27797  Q16610  P16870  P23468  Q16620  Q16849  P26992  P23083  P01009  P07711  P27918  Q8WZA1  P02743  P41271  P01593  Q9NPD7  P08174  Q8WY21  Q92932  Q9HC56  Q8NFY4  P00747  P04004  P13473  Q969T9  Q9UP79  Q9UGM5  P04114  O14791  P35590  P22061  A0A0C4DH31  P25311  Q6UW01  Q9HBT6  A0A0B4J1X8  P01011  P43652  A0A075B6J9  Q6EMK4  P04179  P69849  Q9NYX4  Q6NW40  P35080  O00241  P06744  P03952  A0A075B7D0  P01619  P52758  A0A0A0MS14  P35052  A0A075B6H7  P02790  P13987  P10645  Q53EL9  Q9P2E7  Q9BQT9  Q10472  P21802  Q8N126  Q8NFZ4  P01718  Q15223  P57087  P49641  P01859  P02765  P29279  Q8N475  P01876  P11717  Q9P2S5  P80723  P01023  Q86UX2  Q9Y646  P05060  Q6UXK2  P02654  P07858  P01891  P36955  Q6UVK1  A0A087WYE8  Q86VZ4  Q03167  P04275  P19823  P01743  P02787  Q9NPR2  P43251  Q86Y38  O15197  P40189  P01602  P07357  A0A0A0MRZ8  P02763  P43234  Q16653  Q9BY67  Q5VU97  P19652  Q8TEA8  Q16568  Q9NZ53  A0A0B4J2H0  P22748  Q99497  Q9UN36  P01834  Q15582  Q9UPU3  Q14767  Q16769  Q9NYQ8  P22304  Q9H2A7  P00338  Q02246  Q9BXR6  Q15166  O60568  P01880  Q8NCW5  Q9Y6N7  P52797  Q15485  Q8IUC8  P00738  A0A0B4J1U3  P02741  O14793  O15466  Q6UX71  P14151  P43121  P07333  Q86SF2  Q96RS0  O43866  Q5BIV9  P01704  O95445  P02751  O43291  A0A0B4J1X5  Q8NBP7  Q8NES3  P01597  P08603  P22607  P07686  Q14118  P0DP58  P49747  P80108  Q99784  O00391  A0A0B4J1U7  Q13449  Q8NCL4  Q12913  P01860  P14780  P06312  Q9H8L6  O14917  P13796  Q9NY72  O43157  Q96KG7  Q6UY11  O75078  P07225  P51884  Q10588  Q9UBG0  Q9Y6R7  Q8N3H0  P23435  P23142  P11362  Q06418  Q5SRE5  Q8TDD5  Q9Y4L1  P50750  Q14004  A0A0G2JMI3  P09668  P29218  P13611  Q06828  Q96AP7  P78417  P01024  P0DOY2  Q01469  P10451  O60279  P01833  O00461  P02647  Q13508  Q12805  Q9BRA2  Q07954  A0A075B6K2  Q6ZMI3  Q9Y6N8  Q95604  P12318  Q8N6C5  A0A075B6S5  O94772  P09417  P15814  Q9NZP8  P61916  P02656  O15031  P31944  Q8TDQ0  O00187  P19320  Q13228  Q96CX2  Q9Y617  Q6UX73  Q15113  Q9NY47  P08195  Q9UN70  Q9C0C4  P0DJI8  O43556  Q9NZ08  P05546  P11021  Q9NRN5  P00766  P06865  P01601  O95897  Q8NFP4  A0A075B6K4  O14786  P68363  Q06033  O00462  Q8NBJ4  Q02413  Q02809  Q16674  Q15375  Q5JRA6  Q8NBS9  P04066  Q495W5  Q8IZP7  O94919  A0A0B4J2B8  Q15782  Q5KU26  A1L4H1  P55283  O95490  A0A0C4DH25  P35442  P04430  P07339  A0A0C4DH35  P01871  P14625  Q8IZJ3  Q96FE7  P02766  P00742  Q9UNN8  Q8NHP8  P0C0L4  P60983  O94769  Q9BX67  Q9UBX5  Q9H7Y0  Q9GZX9  O14773  A0A0C4DH29  Q13214  P01857  Q9NS98  P02792  P07355  Q06830  Q99523  P00746  P09871  O94779  Q8NBI6  P63010  A0A0B4J1Y8  P15289  P13521  Q969P0  P04745  Q92752  Q15904  Q92673  P04278  P00390  P18065  Q9HDC9  P02461  P52799  Q8IZA0  P08493  O15354  P05452  P06733  P01033  Q13554  O75223  Q9HAT2  A0A0C4DH39  P09486  P00739  Q02487  Q15063  P01861  P21810  Q96RR4  P02786  Q9HCU0  Q93091  P48745  Q14126  P05155  P0DP04  P11047  Q86UD1  Q99453  Q99715  P07108  P62937  Q03591  Q96BZ4  Q9UBR2  P02747  P02689  P10253  P06727  P07998  P51888  P10721  P10599  P55268  P29762  Q10471  Q13835  Q8N1N4  Q8N3T6  Q8TEU8  P32119  A0A0B4J1V6  P00760  P60709  Q08345  Q8IVN8  O75711  Q9UHG2  P61769  Q14315  A0A0A0MT36  Q6UXB8  Q969H8  Q9H2X0  P30041  P0DP01  P06396  P0CG47  P24043  P20774  P00751  P22897  P02144  P03973  P20742  P34096  P05937  P02746  A0A0B4J1Y9  P16070  P21333  Q8TCZ2  Q9UNW1  Q04760  Q6UWP8  Q92820  P14543  Q12841  P61626  A0A0C4DH34  A0A0B4J1V2  P11142  P49257  Q9Y240  P21246  P20851  O14498  Q9BU40  P03951  Q14393  Q99983  A0A0C4DH24  Q9BTY2  O75144  P23470  Q7LFX5  P26572  Q14165  P45877  Q04756  A0A0J9YY99  Q9BRK5  P30101  A0A0J9YXX1  A0A075B6Q5  Q01995  Q7Z5L0  Q08629  P0C0L5  Q96S96  Q13867  Q14766  Q8WUJ3  P98095  Q8WWM7  P69891  Q6YHK3  A0A075B7D8  Q9P2S2  Q07092  A0A0C4DH32  Q9BQ16  P54802  P15291  P33151  Q562R1  O00584  P22105  P16112  P30740  P54803  O00115  P01344  Q9BUN1  Q53RD9  Q9Y376  A6NGN9  O60888  Q13740  O75503  A0A0G2JRQ6  O15389  O43615  O95633  P04406  P80188  Q8IUK5  Q96IY4  Q99519  Q9Y5Y7  P06276  P08294  A0A075B6H9  P22792  Q99538  Q96ID5  Q99969  Q16270  P12830  P15586  Q9BWP8  P02775  Q31612  P08571  P29401  P04259  Q9NPH3  O43300  P35443  P20933  Q9UK55  P61981  Q08380  P15328  P02768  Q6FHJ7  Q8IVF6  Q9UBX1  Q9UJJ9  P05160  A0M8Q6  P22692  P00915  P04040  O75882  Q9H2E6  P35555  O43529  P09603  P55285  A0A0C4DH67  P15144  P36871  P35527  P40197  P02652  P04003  P23582  P36222  Q13231  P39059  P20062  P02655  Q6IE36  Q04695  P12035  Q9UIB8  P23284  P05090  P02533  Q9HCB6  P10412  Q9UHL4  O76009  O75828  P48723  Q9NRJ7  A0A075B6I9  P29966  Q9UHG3  Q16706  P11597  Q9H741  P01703  P01210  P08253  P13598  P98160  Q92496  Q92563  Q9ULP0  Q14CN4  A0A0C4DH43  P00558  P24592  Q13510  Q13822  C9JN71  P02452  A0A075B7B8  P01599  P16035  P08572  P50895  P02745  P05787  Q6ZRP7  Q9NY15  P06331  Q9NR34  Q13103  Q9NSB2  Q9UQ35  P68871  P08670  P39060  P22352  Q8IUX7  Q9NPC4  P10643  P42658  O60243  P01817  P07585  Q9Y696  Q9UEW3  P27487  Q13790  P08637  P04264  O95185  P04070  P02776  P22891  Q9Y5R5  A0A087WSX0  P19909  Q01459  P50897  P08138  P05089  P30043  Q7Z7G0  P00736  P13645  P10619  P29972  P55058  Q13217  Q08554  P55056  P13929  P22004  P48740  P62736  P07237  P69905  Q92911  P60033  Q5JST6  P06702  P09211  P08519  Q96JF0  P41217  O95428  O00339  Q9UM47  P02788  P14923  O60462  P05062  P49065  Q14112  P15259  P02769  P25774  P38571  P49908  Q8N2S1  O95967  A0A075B6R2  P00488  P24593  Q15828  P26038  P02042  P08779  P26927  P07451  Q9ULL4  Q13421  P78539  Q86YZ3  Q8IWV2  Q92765  P02538  Q7Z794  Q8TER0  A0A075B6S6  P07737  P17936  P12111  Q9UBX7  Q86VB7  Q9Y279  P09382  Q02818  P15924  O00264  P13647  Q15517  O95965  P07942  P09172  Q9BXJ0  A0A087WW87  P12259  P01893  P55083  Q9H4G4  A0A0C4DH33  P08123  Q8NCX0  Q9BRK3  Q9H3G5  P07477  P42785  Q92743  P02753  P07996  P35542  P35908  P48061  Q8TAG5  A0A075B6I0 | 3.17E-06  4.4E-06  4.4E-06  4.4E-06  7.63E-06  7.63E-06  1.44E-05  1.56E-05  1.56E-05  1.56E-05  2E-05  2.44E-05  3.4E-05  4.11E-05  4.11E-05  4.11E-05  4.11E-05  4.11E-05  4.11E-05  4.11E-05  4.11E-05  4.11E-05  4.69E-05  4.69E-05  5.04E-05  6.44E-05  6.44E-05  6.44E-05  7.8E-05  9.82E-05  9.92E-05  0.000110685  0.000110685  0.000111231  0.000117554  0.000126193  0.000128034  0.000129338  0.000129736  0.000155625  0.000176695  0.000177214  0.000177214  0.000212076  0.000216152  0.000216152  0.000232473  0.000232473  0.000232473  0.000251562  0.000251562  0.000262067  0.000263686  0.000263686  0.000266875  0.000266875  0.000286077  0.000286077  0.000295452  0.000295589  0.000295589  0.000295589  0.000297968  0.000297968  0.000297968  0.000301878  0.000301878  0.000329263  0.000330242  0.00038884  0.00040618  0.00040618  0.000450107  0.000450209  0.000460608  0.000493334  0.000494479  0.000516966  0.000536982  0.00056793  0.000576419  0.000576419  0.000576419  0.000644144  0.000671385  0.000671385  0.000678579  0.000678579  0.000706962  0.000708079  0.000708079  0.00071234  0.000725784  0.00073699  0.000747681  0.000766251  0.00078838  0.000808582  0.000821139  0.000828867  0.000836205  0.000836205  0.000851201  0.000851201  0.000898224  0.000916082  0.000916082  0.000916082  0.000919639  0.00092305  0.000926246  0.000950013  0.000953059  0.000992856  0.000998123  0.001006723  0.001021923  0.001053377  0.001090238  0.001121777  0.001121777  0.001121777  0.00112488  0.001221706  0.001318852  0.001365647  0.001408938  0.001411465  0.001450436  0.001550012  0.001554996  0.001641746  0.001679866  0.001683995  0.001683995  0.001725061  0.00186702  0.001892251  0.001897455  0.001938852  0.001981368  0.001999326  0.001999326  0.002086909  0.002147215  0.002180336  0.002194772  0.002474939  0.002533962  0.002533962  0.002677833  0.002677833  0.002677833  0.002677833  0.002677833  0.00272712  0.002746583  0.002750536  0.002781695  0.002864588  0.003045318  0.003088891  0.003088891  0.003158905  0.003203461  0.003405357  0.003485741  0.003648887  0.003801052  0.004072492  0.004195023  0.004337231  0.004516252  0.004516252  0.004516252  0.004517682  0.004538901  0.004538901  0.004799055  0.004876126  0.004877284  0.004982524  0.005049126  0.005149471  0.005149471  0.00540835  0.00540859  0.005458509  0.005531987  0.005585552  0.005585552  0.005607511  0.005968823  0.006469311  0.006469311  0.006723107  0.007513192  0.007621354  0.007621354  0.007621354  0.007621354  0.007783512  0.007783512  0.008206487  0.008348843  0.008349739  0.008391557  0.008608468  0.008608468  0.0087394  0.008799519  0.008941898  0.008941898  0.008941898  0.008941898  0.009050709  0.009136473  0.009357854  0.009357854  0.009454683  0.009546315  0.009546315  0.009546315  0.009783736  0.00979939  0.010384785  0.010384785  0.010642924  0.010642924  0.011048157  0.011418995  0.011418995  0.011448982  0.011523127  0.011581346  0.011632994  0.012018307  0.012339201  0.012879905  0.012892724  0.012977537  0.013142751  0.013250136  0.013250136  0.013250136  0.013289406  0.013681491  0.01465946  0.014828382  0.015028931  0.015307682  0.015307682  0.016169238  0.016244202  0.016295248  0.016395132  0.016437823  0.01670697  0.01670697  0.01670697  0.016836839  0.017226136  0.01729755  0.017640398  0.018183728  0.018438599  0.018744339  0.018987895  0.019044851  0.01936946  0.019431247  0.020092252  0.020610192  0.020915919  0.022395707  0.022452462  0.022454023  0.022602272  0.022911047  0.02339839  0.023578026  0.023578026  0.023578026  0.023638348  0.023762749  0.024335203  0.024430608  0.024533163  0.024600067  0.025067333  0.025100042  0.025733882  0.026814607  0.027096737  0.027156884  0.027964771  0.028079586  0.02853482  0.02853482  0.028891252  0.029376633  0.029558005  0.029612984  0.029643432  0.030153564  0.030153564  0.030456332  0.030456332  0.030476945  0.030660007  0.031151911  0.031383122  0.031383122  0.031987757  0.032994786  0.033940624  0.034148472  0.034148472  0.034434831  0.034456462  0.034456462  0.034456462  0.03474894  0.035192893  0.035192893  0.035917452  0.037007324  0.03719126  0.038892338  0.039656422  0.04021296  0.04021296  0.04021296  0.041032958  0.041032958  0.041493919  0.041623914  0.041623914  0.043518854  0.043802377  0.043802377  0.043802377  0.044446433  0.045278242  0.045278242  0.045614052  0.046386901  0.04850041  0.04850041  0.04850041  0.049644146  0.05096937  0.051520609  0.054454019  0.05678248  0.057586157  0.057990794  0.05859692  0.060243545  0.060843553  0.06101565  0.061587483  0.06165617  0.062137522  0.062890709  0.066059084  0.066792202  0.066792202  0.066792202  0.067723452  0.067750228  0.067829338  0.069475893  0.069656547  0.070696511  0.071322431  0.074667407  0.075301696  0.075301696  0.075800897  0.075878006  0.075878006  0.076132053  0.076132053  0.076132053  0.076400778  0.076423638  0.077361684  0.077486116  0.078509928  0.079146516  0.080325068  0.081359349  0.081784332  0.087769563  0.088248918  0.088388259  0.089167529  0.089676967  0.089685596  0.090926061  0.091348997  0.091978224  0.092523816  0.094424035  0.097031481  0.097236197  0.098694227  0.099266013  0.10006539  0.100844214  0.104264029  0.104669965  0.105058142  0.105202495  0.105202495  0.106181723  0.106522172  0.107036353  0.107036353  0.107036353  0.107513769  0.109036344  0.110268968  0.110268968  0.112274977  0.112274977  0.113374531  0.113944226  0.115846606  0.116528527  0.116659678  0.119580351  0.120153415  0.121110758  0.121224192  0.121487923  0.123113315  0.123113315  0.124535936  0.125173768  0.125173768  0.12623402  0.127217756  0.127217756  0.127262704  0.128754134  0.13001207  0.130221144  0.131367333  0.133112747  0.133239608  0.136282077  0.137151458  0.138055139  0.138391035  0.138391035  0.14000417  0.143739515  0.143842858  0.147295365  0.149386738  0.149698372  0.150920386  0.151292866  0.152181768  0.152668548  0.154983565  0.158419859  0.158419859  0.16060645  0.160714349  0.161257149  0.164343331  0.164435352  0.165093077  0.165146096  0.165146096  0.16545548  0.167276673  0.170987724  0.171632636  0.1744342  0.175032042  0.178337418  0.182945075  0.184247943  0.184247943  0.184247943  0.184247943  0.184801186  0.184920885  0.188200871  0.188200871  0.192777221  0.195619419  0.196804889  0.200972549  0.202224325  0.202381096  0.206029815  0.206374042  0.206374042  0.211712298  0.213045143  0.21592107  0.221824238  0.222859334  0.224609663  0.227082727  0.227978159  0.229321404  0.231164089  0.231164089  0.231164384  0.231164384  0.23191649  0.23709518  0.238850205  0.239199663  0.239362195  0.240870712  0.241462024  0.245512027  0.245751469  0.246800157  0.250312491  0.251157937  0.253209206  0.259255163  0.259255163  0.2642701  0.264849897  0.265947201  0.265947201  0.265947201  0.265947201  0.266206708  0.266733413  0.268138099  0.268138099  0.268138099  0.268179179  0.269415475  0.271072303  0.27820433  0.27820433  0.27820433  0.288527627  0.296416692  0.296416692  0.301692268  0.305549053  0.305549053  0.305549053  0.305549053  0.305549053  0.306720724  0.308343007  0.30929586  0.313625356  0.320034072  0.320034072  0.320773091  0.321860089  0.32495104  0.326216937  0.331507894  0.333075566  0.333075566  0.333075566  0.333133544  0.334675689  0.338031736  0.341674501  0.350940043  0.35303077  0.353231807  0.353299476  0.355478273  0.355478273  0.358447155  0.358447155  0.363318454  0.36493163  0.365637626  0.365637626  0.365637626  0.365637626  0.365637626  0.365688142  0.370350663  0.377534432  0.378862161  0.378862161  0.38167712  0.387285892  0.390772751  0.390772751  0.390772751  0.390772751  0.391527755  0.392320447  0.392828475  0.398735496  0.401384208  0.401543364  0.401543364  0.401842002  0.401842002  0.403300695  0.403300695  0.403716937  0.418205599  0.41863131  0.420668914  0.427331515  0.429327567  0.430085942  0.43148293  0.432304531  0.434942157  0.435120146  0.435774922  0.4399732  0.44410853  0.44552498  0.446982495  0.446982495  0.447200315  0.448839942  0.448839942  0.451280452  0.451280452  0.451997726  0.45458179  0.472340786  0.473056699  0.478671038  0.484382748  0.487055299  0.492114344  0.492114344  0.494645011  0.498624061  0.505771707  0.505902168  0.506896027  0.508783102  0.508783102  0.508783102  0.511967976  0.514979538  0.515428887  0.522052268  0.522199466  0.524323581  0.526129111  0.526129111  0.526129111  0.526129111  0.526129111  0.526129111  0.526129111  0.526129111  0.526129111  0.526129111  0.527383114  0.527383114  0.534602271  0.535496821  0.537582251  0.537794921  0.539658157  0.545204146  0.551966939  0.558263473  0.560354892  0.565074583  0.565074583  0.568339281  0.568339281  0.571285647  0.575673967  0.575993444  0.583150173  0.583324888  0.591640579  0.594701602  0.600350316  0.600432651  0.600991616  0.620948177  0.620948177  0.624401439  0.6262353  0.626289918  0.63025476  0.63025476  0.63443526  0.634535355  0.635156017  0.637519054  0.640984743  0.642904967  0.642904967  0.643861264  0.645528423  0.649295765  0.654075484  0.654171205  0.655247214  0.655751485  0.655751485  0.655751485  0.655751485  0.657376493  0.658860244  0.660940587  0.661162021  0.663189983  0.667515033  0.670737033  0.670737033  0.671698736  0.67650219  0.68010943  0.68010943  0.68465396  0.68465396  0.68628196  0.68667511  0.68667511  0.70018306  0.701964065  0.701964065  0.701964065  0.703312255  0.70350551  0.70350551  0.706308539  0.707460288  0.707460288  0.707460288  0.710734179  0.71590162  0.720040385  0.720040385  0.720121662  0.731252382  0.731252382  0.731252382  0.731252382  0.731252382  0.735467098  0.735467098  0.736007124  0.736007124  0.736007124  0.738345991  0.738345991  0.742240395  0.742240395  0.742240395  0.745098022  0.745502691  0.745502691  0.745563131  0.745563131  0.745563131  0.746255295  0.749116572  0.749116572  0.750811289  0.750811289  0.750811289  0.754170644  0.754745647  0.75484119  0.75484119  0.75484119  0.75484119  0.757341006  0.761687183  0.761687183  0.764159523  0.764586926  0.764746856  0.764746856  0.764751048  0.764751048  0.764751048  0.767319199  0.768613247  0.769791149  0.772384839  0.775513809  0.780245895  0.788859229  0.788859229  0.789019229  0.789019229  0.792065525  0.796182946  0.796182946  0.796888083  0.803060585  0.804475683  0.804650533  0.804650533  0.804650533  0.804650533  0.806351254  0.807246896  0.814740408  0.817108596  0.817108596  0.824314049  0.831481701  0.832696485  0.832696485  0.833166451  0.835664331  0.841694348  0.844976621  0.845233061  0.855512125  0.856630303  0.856684347  0.856684347  0.856684347  0.860159336  0.868687315  0.871192916  0.876475812  0.876475812  0.876475812  0.877197942  0.880293561  0.880293561  0.880293561  0.880293561  0.881960525  0.883614628  0.883614628  0.884944652  0.887514735  0.889046797  0.889140559  0.895010253  0.895010253  0.895257206  0.895257206  0.895383661  0.895383661  0.901913356  0.904288019  0.905751692  0.914320778  0.919783727  0.921266037  0.922229193  0.931236416  0.932447421  0.934215409  0.938880564  0.939772391  0.940502892  0.941752994  0.943292378  0.943292378  0.943292378  0.943292378  0.949143101  0.959623192  0.961491765  0.96159212  0.961909932  0.962047882  0.98051098  0.98051098  0.98051098  0.98051098  0.981626042  0.981626042  0.984510327  0.987176451  0.987176451  0.987176451  0.987176451  0.989253884  0.989326411  0.995831878 | 7.556666248  7.899857514  8.589335741  7.741343686  9.919768657  7.858419569  8.147260469  7.313562054  8.863554653  8.553824492  8.328523371  7.813201758  9.013597012  8.980009047  8.207463666  7.156438125  7.399863067  8.049477579  7.758418369  8.081563338  7.766166433  7.27597613  9.243265304  7.234703323  7.812961708  8.95164822  7.286697008  7.655393251  7.517053031  7.588687348  7.722471324  8.364742151  6.752067558  7.588257835  8.793659142  8.255309609  7.109225684  8.447039918  8.085343894  7.45200086  6.548824444  8.462826152  7.724371463  8.184565266  7.235955919  8.406341948  6.778664946  7.24925744  6.579514313  7.004351325  7.928551848  7.946945254  6.582172653  7.918610897  8.206298  7.656117509  7.767295516  6.894660138  7.25278693  8.504429699  8.504333926  8.026475333  7.895585688  7.952321983  7.419250298  8.159236212  8.805312149  8.414778405  8.136691831  8.321538612  8.287084708  6.793660768  7.44677234  7.559639831  7.985142725  8.550979561  8.865140289  8.168654058  8.32058402  7.685190146  7.310240289  9.851777263  8.235167968  6.604626411  7.852086195  7.376445602  8.226448098  6.008059829  7.900016612  8.710232599  6.694066735  8.737918783  6.915025862  8.489372392  7.39265302  7.347075171  7.911742555  6.946508206  7.635983503  7.555024423  6.928424942  7.635057421  8.472986021  7.222528512  6.705467912  8.533318892  7.894471983  7.05467086  8.843910403  7.940335483  9.406288472  8.171012531  7.314490553  6.666849223  7.160636858  8.069277308  8.158162545  7.843329338  7.009175712  7.196677372  7.006144436  6.859768082  9.513661748  7.334658614  6.342673053  7.696287766  7.092195039  8.669481395  6.669231004  8.123715173  8.171274055  7.247566297  7.442552651  8.194706156  7.462878578  6.540467063  7.41160157  9.089320207  7.947831522  8.01941707  6.079293283  6.257459163  7.314551474  6.304199571  7.400762987  6.485555789  7.205743859  7.701264791  9.738279978  7.10659289  8.93524671  7.666570214  7.575580755  7.274924166  7.092021491  8.206978194  6.701477665  7.433671083  5.613239133  6.963283626  7.924089775  7.515020581  9.056611043  4.783306279  6.856184934  8.254073244  6.284703647  6.865760068  7.458600654  8.014263174  8.379215866  6.103750777  7.685453761  9.788109342  5.956317446  7.51269162  7.338068768  6.745341257  6.781816844  7.969457625  7.506431562  7.616018428  7.141941098  6.34065426  8.542701703  7.763522316  7.232369679  5.394889993  7.357801711  7.5459194  8.404870114  8.397624452  7.570334769  7.421277996  6.651010623  7.097821738  5.819587217  9.45457766  7.470259102  6.025901876  7.648612655  6.927483076  8.459698098  7.212459791  6.959762751  6.959108681  6.295633902  6.904704618  6.693807653  6.873356458  8.589955907  8.604870678  8.051864457  6.605287566  6.355449974  7.16913829  8.439054182  6.584173639  4.940021689  6.65056786  6.352392271  8.498773849  7.053579535  6.014788991  6.560147445  8.997859141  8.149360566  6.511668332  7.404026541  6.396106988  4.681807986  5.66249072  7.13045598  5.595199859  7.113832572  6.387746131  7.501072699  7.164575163  8.339964179  6.181539575  6.121475133  7.026326486  6.37629905  9.534434627  7.943363785  8.566718598  7.732691279  5.974987904  7.051731373  5.909019913  7.22662177  7.798165285  6.7665108  5.331823087  7.208626173  6.0802199  7.949481665  8.110657138  8.37539957  7.109797265  6.799059183  8.481805585  5.873887551  5.325537696  6.957068706  9.422444728  7.754144158  7.81860297  8.514789341  6.107352212  7.386775378  7.54270579  5.619756731  9.339515438  6.346309329  5.974808526  5.619080053  5.510418421  7.560863655  7.931035033  5.977746052  10.5233207  7.078058547  7.967213444  5.698958485  6.525904878  7.383259281  6.377626297  7.476827011  8.226803943  8.900153325  5.66356562  7.201280259  7.64107172  6.189352323  8.497543856  5.022999452  6.345000837  6.743401055  5.64448401  7.009737845  6.839896742  6.640974453  9.332089753  7.609751196  5.795559186  6.758626642  7.196255  7.839131927  7.476451838  6.96877843  6.659893393  8.516693137  7.247034065  6.672206208  3.573276712  6.482082986  5.937382734  6.80673898  6.801946058  5.562853077  6.636311851  8.554154124  6.001591256  5.943835687  4.843113164  5.797968535  7.356278499  6.46833513  7.786640351  7.820281816  6.733193371  6.5119743  6.498994468  4.505527762  6.002397135  6.838266434  9.162889278  5.754293972  7.67400417  5.225707921  6.16369212  5.258242134  8.903878202  6.736753837  7.039473621  7.874639756  7.217396076  6.235500142  6.725446229  6.875250633  7.78220117  7.750146226  8.035055759  6.220528437  6.120933013  7.762853901  3.789634323  7.685091754  7.364767043  5.860670572  6.711596992  6.467267548  6.065150866  7.317842025  6.188975486  5.980315135  8.179410803  7.988401426  6.088967985  5.940222036  8.246141982  6.234444589  6.907304283  9.007431124  7.452783092  6.740058665  6.842604169  6.842604169  6.558452727  7.406176366  7.406176366  4.901447965  7.365662106  6.188835919  7.4822493  6.411297501  6.539351847  6.591551563  9.778496171  5.936393944  5.605244772  8.310644392  6.424828057  5.135941537  5.837584533  9.410273738  7.264692898  8.990669038  5.550710377  7.358312484  5.941809246  6.815330136  5.945692435  5.274525431  4.879145096  5.845349162  4.557179503  5.291761427  7.1787783  6.155693582  6.386440236  8.316511112  7.108975426  7.529193238  6.21690893  6.551958691  5.223289296  6.704881226  7.862285297  5.879650771  6.476536478  7.42346195  8.506149297  6.521852396  6.577408966  6.353280255  4.403572621  5.04324653  6.014165433  6.380097672  8.345482573  7.515926682  7.570695697  4.675187946  7.051107058  3.958955789  6.287956829  6.799510269  6.463567698  6.599371482  5.748154131  6.397540115  7.265505065  7.064262262  7.666782344  6.181820527  6.544412103  6.46362547  5.839147252  4.388973705  7.268300163  5.80180034  6.390819435  7.459889289  4.984638594  6.200476325  6.934697448  6.225671421  6.146547547  3.314691424  7.750854922  6.298489386  6.057047643  8.766902791  6.736803518  7.881092955  7.710821385  5.046297941  7.604653621  10.04523138  7.532961426  6.729071199  6.146433846  7.254270239  5.810245386  7.172033754  5.755595786  7.506531348  4.862369302  3.93574718  7.514823676  5.915467879  6.420282364  9.758343133  6.697098308  6.408431999  5.684293228  5.958364633  6.5761454  7.772132908  8.585283151  5.972947037  6.211290228  4.672862763  6.219564565  5.925134054  6.757518379  7.819779615  5.468778278  6.80407613  7.158141475  6.603836469  6.923626102  6.380576575  8.382872946  4.555830647  7.100737684  6.412117958  4.794509067  6.122420933  5.637621181  8.518633985  6.850705769  8.135932422  5.851376677  5.506659227  7.336943407  5.868414528  8.001176196  6.708085968  6.041113235  4.656562131  8.023722888  5.078563622  9.320526639  6.021947058  5.541299282  7.316591795  6.909126937  6.206696999  9.198533629  6.899525385  7.379944995  6.59769072  6.230253288  5.065169327  6.331447477  6.882670397  4.756246742  6.653621138  6.858329957  8.182708767  4.077363239  4.433170503  8.486436064  7.737234235  7.277654928  5.706590314  7.046510741  6.774469557  4.911258015  6.863340814  5.28842345  5.911903659  5.131587655  7.654698327  6.912986973  4.533514063  8.542257559  6.716883056  6.39221133  4.539886756  6.190484228  6.47082249  7.827266155  5.282447378  3.191412026  7.249933131  5.260054367  6.41666388  6.453705809  5.104102856  8.978527972  7.444164872  6.592347438  8.604159232  8.743668369  6.87248729  7.140187022  5.817395747  7.695566894  7.187882988  6.146000245  8.190130986  7.007371209  7.905217649  5.578427927  6.639065594  7.010427587  5.380829163  4.586267931  7.308239972  7.259035614  6.922312659  6.989938404  5.668152193  4.558944789  6.133535463  5.57965581  6.771728596  6.530537897  5.203814729  7.803350564  6.690748595  6.037936631  7.110065627  7.474630168  6.284647253  6.824314345  7.463719264  7.940405257  5.890370916  6.56759699  6.54762405  6.477962496  6.314437164  6.326212738  6.182746208  6.050181367  3.647000497  5.416313199  5.815196679  5.365107965  5.898788095  8.078273842  7.627049947  6.147610029  6.423474761  5.496681669  7.085312736  7.131254736  3.486476986  6.151627095  4.875558707  8.625078459  5.739531631  6.017156998  6.675599956  6.37467669  6.62717555  6.144508912  6.217181412  6.999833059  7.443139616  6.843853678  4.690865872  5.123683975  6.250947951  7.950132581  6.156453103  5.621172863  6.612838071  6.568642417  6.640468637  8.059198079  5.896529339  6.527039579  6.251428027  6.606503583  5.217692685  8.021524496  4.568749141  5.26081693  7.216318995  5.705337716  6.865759784  6.645116869  8.319674108  5.92998133  6.867455555  5.254717373  6.173796148  7.117557205  8.735636092  4.179963017  6.504837714  4.510492969  4.066901574  5.434872086  8.458305356  4.308005951  6.921442791  4.569627189  5.799164024  6.787226685  6.61926382  6.663758183  6.751345118  8.416376237  5.882887359  10.88054712  6.478577775  4.661513977  6.992867976  7.058603783  6.710154648  5.61506645  6.625305457  6.682468564  5.758561352  7.699345789  6.727821922  6.65398868  5.47061109  5.890028068  6.185165604  7.302416294  7.269713576  4.269415663  8.986852894  7.115565164  6.517934306  7.903770167  4.949875744  8.329033371  5.205499866  6.527237728  7.033202773  5.349719183  6.513205103  6.838717832  6.530175812  4.115110634  7.683437641  8.94926784  8.008739285  7.575488698  5.710037134  7.297944786  3.123938745  5.562225426  6.518221596  4.526948608  6.631284818  4.997822846  8.125213112  6.798424428  4.449558929  6.169876841  6.023609725  6.201476611  7.585196493  5.219319639  8.014090619  6.244829475  5.971243349  4.249735528  7.43864828  6.861091565  7.079919255  7.588778737  6.903399599  8.993064587  6.394768342  7.766688585  7.126643033  6.437232211  7.316096381  6.003537549  5.522179315  7.015704  6.81073737  6.033245571  5.162467407  6.414555187  6.060956058  4.865631559  7.802643222  6.832847119  7.482900276  5.199149523  7.813797143  8.130414502  7.233767324  5.226576463  8.445660982  7.230467096  5.983729533  4.918363609  7.436679823  5.238382825  7.001900835  5.136567906  4.965971496  6.62675058  9.081115417  4.933863415  6.698577705  5.213978169  6.363814593  6.039926609  4.930719018  4.842612141  6.47180417  5.413879161  4.4686026  5.326540756  4.167951504  4.615820884  8.277148509  8.818246899  8.458162567  5.857434454  8.376485265  4.680566416  6.043954727  4.154585998  5.576432792  5.339696497  7.220357281  6.12883552  4.806433481  7.484385706  4.606938802  4.332183664  4.692914177  5.950188527  6.62954892  7.218479243  4.885935358  6.291147928  6.170299935  5.07289124  6.03685164  7.220681882  6.15567722  7.736776693  4.715430516  7.283409273  6.99322051  6.427980813  7.966311745  5.983394866  5.77272705  7.619718714  7.589509263  7.184598477  4.969519183  4.874838205  5.787290525  5.84936798  6.688210768  6.380188453  7.463888657  7.802680874  5.652230066  5.775671997  5.383967653  5.422283142  5.511800628  7.071689449  7.449301622  7.380443886  5.234978339  6.272583291  5.124661665  6.326046601  6.715719696  8.122075266  6.702634496  7.339383509  7.012230578  6.782434731  6.101752967  7.332925171  4.50269842  7.828435604  5.53777192  7.355844813  6.922431877  6.507350413  6.848012065  6.456629074  8.031802218  6.309201996  6.486192268  4.117291619  6.281728463  7.60127571  4.557059883  6.098398184  7.388325903  7.274642073  7.275309587  6.961425475  7.986039318  6.948184799  6.990847609  8.588013849  6.254134103  5.73627083  5.729924519 | 7.231576458  8.180650771  8.278821217  7.415487133  9.589405955  7.514716052  7.894636151  7.71528511  8.495057864  8.277003363  8.033840921  8.160338604  8.716081632  8.649737999  7.83735882  6.944113968  7.819063822  7.797686194  7.463936355  7.838101197  7.453003316  6.996064562  8.8981401  7.012892832  7.457288334  8.68079427  7.006850397  7.443561745  7.890484704  7.845262907  7.545428198  8.078819726  7.218702178  7.157541988  8.482173994  8.049836907  6.945447573  8.142198595  7.879803585  7.055829453  6.241322087  8.241898896  7.993678338  8.41821124  7.743060778  8.169548224  6.285861647  7.653157832  6.254260244  7.351650394  7.708653897  8.333890014  6.200127548  7.674733267  8.602894801  7.363210182  7.979724687  6.614284648  7.01163032  8.171152795  8.272438252  7.819644101  8.22960375  7.651185567  7.21517896  7.941519875  8.453902493  8.655316659  7.891508173  8.094015241  8.038884829  6.547516942  7.146262938  7.269497358  8.270906754  8.35920485  8.702804494  7.895247344  7.995793634  7.462563151  6.975441572  9.573652468  8.490813872  7.036580867  8.134699406  7.035208688  7.962504103  5.104902934  7.623647318  8.933352099  6.380781364  8.386488777  6.604932873  8.281373493  6.94993779  6.941057728  7.645300886  6.616551494  7.377107009  7.217065996  6.648179002  7.438600853  8.236445528  6.534501376  6.16359517  8.732367951  7.672752664  6.588059743  9.016804218  8.30964776  9.133374472  8.452972216  7.105686893  7.161620822  6.832663874  8.364219675  7.988942232  8.136446616  7.332560556  6.945232397  7.390433053  6.613547846  9.304387394  7.612785469  5.959031068  7.9244732  7.371498557  8.856249674  7.037150674  7.934885881  8.003166996  7.517489752  7.740922299  8.022708262  7.114869444  6.897928053  7.009696653  8.897193469  7.667910543  7.787511485  6.894180739  6.665666455  7.021168101  6.719922031  7.644876342  6.217386078  7.027913153  7.494151023  9.571679249  7.395766681  9.110692612  7.478313942  7.386268951  6.949362608  6.806344293  7.920293557  7.061355605  7.091584662  5.104151956  6.727554555  7.573231001  7.737917231  8.860185511  5.288119971  6.58856063  8.013529831  6.73429622  6.494371079  7.240951798  7.871713349  8.56980083  6.60693883  7.516647154  9.548396938  5.248665495  7.749472903  7.107239191  6.276964787  6.605234535  7.726461997  7.361645091  7.471614701  6.813579496  6.093615042  8.412423175  7.976680811  7.033485548  5.918608547  7.172141042  7.377851469  8.250412606  8.25120485  7.399800141  7.1256567  6.110980386  6.86218703  6.481769556  9.625871984  7.313567483  6.252186761  7.488311062  7.363384463  8.180707287  7.473995157  6.707745568  6.763465178  5.695303365  6.540820438  6.296098143  6.685167252  8.760622975  8.753572644  7.857979037  6.344527905  6.053224565  7.569261686  8.685701498  7.100045269  4.343564041  6.231518792  6.733707774  8.642630712  6.771180118  5.539200232  6.889531701  9.144966706  8.399156009  6.849660171  7.257751347  6.185972434  4.122979864  4.968189733  6.873474889  5.039233246  6.842687622  6.167253669  7.709503396  7.605582026  8.517791932  5.845178671  6.547959802  6.760196735  6.740755077  9.67764995  7.774105767  8.136851525  7.559050642  5.555751575  6.793852469  5.533648201  6.993038515  7.636660379  6.576034476  4.890802243  6.99729947  5.728014786  7.758126143  8.359968106  8.56215514  6.815881962  6.598504977  8.794196466  5.533900766  6.035606994  6.2936066  9.327966067  7.63693628  7.70599817  8.241288104  5.585145386  7.634209305  7.400283725  5.104269605  9.21101148  6.173356922  6.476881207  4.893593733  5.125344599  7.71022148  8.088949349  6.414464987  10.39126373  6.939840006  7.867750989  4.973773249  6.073236124  7.241857682  6.752019049  7.771431576  8.380194848  9.124748078  5.176887793  6.981545915  7.43536442  5.752707164  8.735827329  4.544849629  5.676140268  6.424007105  5.9513471  6.805535543  6.625653742  6.250464708  9.4969803  7.727024543  5.33112301  6.991584415  7.009137583  7.701884943  7.244618754  6.868393307  6.44689212  8.398726449  7.112666394  6.968700893  2.312208591  7.015543949  5.65034666  6.596730599  6.662911021  5.914203851  6.448817922  8.941280173  6.275181917  6.497517724  4.424828917  5.334389605  7.248330837  6.647771987  7.655229766  7.660964305  6.450776057  6.262656688  6.7697411  3.922860562  6.533316053  7.133278841  9.065109393  5.364100075  7.837027655  4.933629579  5.986847486  5.704728405  9.001653584  6.432298682  6.881246411  7.747624045  6.936988463  6.538806636  6.892423219  6.568988413  7.707783481  8.067255152  7.871924432  6.003331566  5.960022265  7.945443452  4.317474732  7.89579215  7.284349815  5.501545598  6.883080162  6.134881809  5.785412502  7.120495316  5.949879738  5.580038139  8.239275816  8.135233677  6.628352099  5.596154924  8.388482863  5.820925491  6.72832431  8.909319619  7.337514131  6.425486433  7.025193593  7.025193593  6.283678558  7.293414422  7.293414422  5.414446625  7.183739822  5.917316418  7.39622887  6.583087192  6.395948374  6.317183951  9.836046093  6.382991323  5.320938789  8.154227615  5.929926358  5.572864901  5.557288099  9.5735831  7.166475562  8.911225336  5.215146342  7.232935688  6.14830189  6.623545728  5.667263263  4.774206112  4.565801536  5.537582393  4.834901684  4.743227063  7.066431719  6.550868407  6.557383096  8.225991291  7.333578299  7.370878414  6.418966411  6.421750162  5.939827841  6.921946219  7.71481693  6.080845871  6.134841441  7.337497916  8.449423805  6.327340968  6.403563527  6.14710116  3.974142634  5.479059342  5.814368245  6.185715892  8.451996397  7.444713665  7.461634114  5.001508827  6.940981563  4.560381242  6.084454657  7.23286476  6.648745978  6.470272808  5.398137414  6.637958275  7.181754413  6.847100913  8.229233222  5.926005564  6.753506235  6.345582961  5.63056023  4.11032429  7.13055739  5.584081261  6.181007461  7.352123332  5.339395717  6.585499982  6.631172481  6.011060934  5.934274407  4.179628194  7.885045078  6.436262888  6.237879893  8.655662658  6.952248375  8.017254818  8.089945839  4.662065265  7.301893802  9.931334333  7.642980334  6.625612602  5.946422941  7.454220629  5.578270882  7.277039898  5.415734904  7.457741918  5.390657791  3.670634049  7.385720894  6.245667615  6.166311143  9.874131708  6.580513913  6.206732827  5.984464349  5.704351033  6.405478317  7.873784196  8.537077478  6.200121059  6.089210252  4.343714385  6.632920117  5.621424496  6.463850408  7.757779762  5.218300433  6.650438067  6.912480872  6.416870782  7.116629193  6.169031342  8.269796071  4.876162273  7.185462212  6.316176949  4.569033443  6.309000244  5.3302157  8.453286428  6.762616739  8.194532037  5.645208931  5.215125604  7.221747475  6.239702694  7.92530645  6.798994668  5.900796094  4.990547956  8.263209337  5.625048479  9.180200383  5.617313475  5.004959743  7.255656233  6.757241576  6.085764946  9.16387246  7.103038363  7.68859122  6.50180262  5.951299995  4.718954215  6.056712773  6.786988457  5.327452147  6.475498018  6.787463007  8.125147183  4.468979393  4.71715765  8.62170067  7.604791261  7.34849212  5.38453746  6.943271198  6.680744848  4.652313569  6.784516738  4.885782213  6.060250622  4.596194306  7.587870412  6.779083993  4.734700118  8.442906656  6.479696081  6.179210752  4.831236672  6.036354687  6.293805055  7.703955815  4.790313737  2.498697159  7.322649847  5.067034102  6.315862153  6.36285356  5.544006637  8.942722923  7.321621341  6.674710878  8.56088232  8.859924452  6.790620001  7.549272201  6.04616154  7.885551667  7.250690191  6.032612068  8.153593032  7.246396715  7.837854761  5.777856129  6.429268611  6.952199045  5.185867089  4.216090585  7.240725702  7.181666657  6.874577386  7.094987421  5.928516525  4.978676376  5.92569888  5.413221715  6.672400463  6.401869871  4.803435236  7.757200417  6.612877733  6.19930967  7.053741463  7.420329089  6.54221223  6.684663651  7.367355464  7.77219729  6.015285096  6.501417941  6.494001995  6.390609091  6.53264502  6.548447722  6.041315804  5.894421307  3.976736231  5.676904984  5.994442317  5.112848864  5.733924521  8.142429018  7.562603228  6.229097071  6.334839315  5.361043196  6.982012418  7.226374102  3.107498233  6.040766167  5.104643539  8.554909729  5.863760929  6.226087328  6.601474446  6.275017563  6.389365495  6.237808031  6.085069486  6.955412577  7.508855366  6.631339749  4.545693597  4.938832557  6.146433862  7.990674756  6.064201001  5.480625717  6.927748589  6.436755727  6.540084129  7.97794619  5.799231366  6.68150114  6.0523601  6.529930793  5.40206728  7.957493862  4.762949815  5.101452098  7.406301789  5.573645147  6.80410425  6.5844619  8.260841427  6.120723358  6.989803464  5.395472377  5.998819289  7.184877786  8.67602526  4.395786183  6.415205547  4.65109423  4.322297301  5.237433237  8.42288883  4.577038618  6.694869494  4.754453922  5.671594017  6.738212086  6.514537966  6.819356158  6.700710084  8.392887028  6.068705154  10.84673252  6.544292333  4.993955813  7.027552695  7.098122709  6.642473918  5.855815345  6.544403948  6.819862611  5.885636706  7.71839219  6.62231714  6.713700384  5.276965401  5.801370897  6.10836321  7.278407404  7.187805397  4.365733623  8.927157356  6.954254857  6.624916652  7.762788494  4.696135136  8.283145768  5.335540216  6.591976896  7.071500835  5.470087072  6.544471233  6.964325847  6.603040902  4.470695056  7.649562547  8.921234191  7.947886937  7.469703363  5.880187119  7.242930191  2.63981895  5.50146116  6.457071745  4.68990549  6.402625033  4.830326167  8.181362218  6.832800341  4.544970357  6.097834491  5.824590141  6.105196384  7.614735965  5.298647369  8.04077036  6.321446378  5.903856372  4.147134921  7.554122982  7.021592435  7.177773702  7.542815916  6.878845893  8.971843091  6.478773614  7.80085941  7.211670694  6.331103024  7.338384802  5.925758268  5.461496918  6.952565755  6.898855812  5.97808169  4.955333492  6.487473185  6.101894186  4.78430027  7.760288457  6.765312584  7.334523671  5.343405649  7.831241506  8.117163391  7.256567913  5.339946831  8.466694335  7.285972636  5.900321158  4.783937873  7.406567307  5.134501476  7.045909186  5.041745451  4.830198527  6.583031498  9.050991519  4.865030444  6.734748521  5.281488551  6.418344787  6.168108051  5.008458316  4.978512835  6.435006572  5.339109021  4.407394936  5.169317127  4.355968876  4.531042741  8.289482373  8.788826557  8.40681874  5.782767632  8.39577752  4.608136893  6.109959662  4.20675207  5.512632741  5.274145957  7.193181937  6.05810553  4.747704132  7.565643922  4.459267187  4.376052598  4.651426541  5.99872252  6.613634579  7.178847395  4.785593216  6.371126533  6.133020488  5.033461688  6.011372052  7.240642695  6.2072955  7.77380912  4.669607943  7.340837182  7.01096298  6.413887655  7.952201363  5.95321284  5.79749233  7.631249748  7.571466481  7.203631534  4.884399944  4.911505966  5.715380901  5.806435716  6.671040023  6.417212456  7.50679572  7.85835015  5.711024507  5.805105516  5.426845982  5.379754401  5.548982807  7.047579956  7.455444583  7.347868986  5.279070902  6.256263196  5.071054362  6.342804695  6.699381057  8.149636337  6.692377245  7.34500479  7.021662116  6.774783907  6.07328175  7.315483928  4.522364773  7.839559969  5.56502693  7.38519466  6.908778986  6.519280241  6.842145607  6.467070809  8.036407246  6.298408401  6.478873006  4.12562329  6.270281868  7.60445944  4.56301479  6.094206779  7.385117981  7.270316105  7.273333545  6.96347354  7.986909759  6.943126269  6.984385872  8.58603293  6.256035611  5.733521821  5.731939017 |

**Table S4:** Metabolome clustering based on age, proteins reported by BinBase identifier numbers and Binbase name.

| BinBase ID | BinBase Name | Adjusted p-value | Mean Young | Mean Old |
| --- | --- | --- | --- | --- |
| 414134  79  97698  110889  382579  414015  14721  1718  130396  416738  418093  424855  16826  87705  382090  384891  394878  1679  31263  46258  354282  14759  1912  6  19  1380  1862  1871  1977  31858  46307  46362  100321  104131  110018  119054  120562  135613  376650  377697  382585  384894  391739  391842  391853  397709  4716  3163  21725  127  4930  26741  109435  209171  345404  378013  46315  33006  3191  4577  5244  41833  41989  125  68  17651  408463  342736  4550  217691  136398  110343  390143  3009  210312  329430  390137  11  412430  384906  4541  365431  356984  401124  33429  31357  342712  31563  4534  2000  2039  342919  329435  16872  210249  187846  382905  209679  111399  370189  4705  7458  18157  33037  390188  390549  20902  209167  100107  100248  171020  64742  18197  21502  391855  1693  4757  34183  7876  133583  114256  401723  31632  251689  46377  168861  407455  22969  97456  1966  371262  2193  18176  1688  210803  161875  110403  231137  45351  344959  16661  231695  5691  46128  367353  372461  351512  421453  84087  1799  390140  108834  347424  94  356347  97870  327545  100841  391858  110873  370230  32000  133264  3204  2005  379374  4  4600  356926  21714  131558  52863  211988  223087  346241  413693  16942  133605  43100  1391  172  4879  592  390194  101023  11831  53486  356941  7362  395912  384948  169  4746  85651  371557  6529  100335  118869  210653  346561  135260  347190  65  370223  390156  135615  341970  4723  98  102661  4548  31601  45341  380462  3029  26755  27145  223106  215853  87783  1743  391740  168  120665  231827  390185  2821  42194  1723  214112  357377  118816  241684  110022  4721  14817  133590  364462  405512  47235  95440  46123  86075  249248  26713  228052  146  20282  14736  105141  113643  119066  360841  376648  419123  48608  342924  76  390192  3171  100017  100036  372446  109386  3187  17225  54  367427  46292  170278  351271  97455  11481  18407  100897  109990  26736  84185  41924  170069  224095  217920  355607  17297  22021  360226  18223  413  12444  128767  85112  168874  377304  390397  17982  33210  46143  161365  31356  4081  95409  223203  352123  148  112501  111823  16747  131144  390198  346884  25922  41866  237707  365909  380514  390131  112530  31547  388180  31743  4533  782  5990  1692  26704  97834  210712  390144  100556  91  45  1687  2080  4983  130640  250586  230854  20961  34113  583  43180  390179  130716  10  1715  131996  84521  108825  390218  8270  34149  101725  110805  34014  106623  5857  97747  133780  210714  28  3063  134  4605  341715  17267  377751  213180  382599  17664  395982  29  770  6324  31552  171972  379924  34163  4833  87822  41683  126391  188569  16691  21703  165  3167  125830  107960  1982  384956  379063  22735  50366  391898  119025  371571  35  1771  145501  377103  190066  110612  89050  360154  2543  2551  351222  102714  384938  110021  133434  31695  404192  171967  119129  288  5393  6376  88993  1981  329772  171970  1996  61  18173  349293  5259  380761  384978  109464  31559  320847  224096  390155  360150  171298  321077  32158  376949  2670  390135  92  341711  100  115876  41996  98022  15370  573  5837  160  4877  84213  253093  355471  17958  345452  413543  458  112264  89045  4543  100343  357374  3  34081  30  161085  210313  162  3256  1741  1684  110008  408450  412761  4545  892  21683  1690  20903  100326  100876  3232  12265  171564  357409  416577  18386  3470  307  110355  112544  228818  372453  1712  88994  1677  377384  31  97687  210697  85113  284  112556  223080  88981  7490  31223  44509  4529  234947  390122  25  18488  12267  110341  376823  1373  104901  33142  8240  41682  359963  104394  391864  16  3169  34023  121468  391859  9489  247758  188923  121467  228119  127847  34110  4929  209677  14  6432  94582  11214  413572  210239  4219  34097  34089  17947  17883  33282  88992  21705  171969  353342  1208  1673  210231  15  6646  386801  390222  110258  485  7543  3173  119023  135253  364413  2044  208  40  344793  210343  365939  31764  12  356925  330287  104444  33  127277  69  105066  1979  11353  189936  210272  294  1821  4546  110321  120744  110328  1669  34007  34100  131421  1683  7501  84524  161876  2448  210790  31889  3294  110881  395262  1663  389434  5884  6355 | 414134  aspartic acid  97698  110889  382579  414015  14721  fumaric acid  hydroxycarbamate  416738  418093  424855  citramalic acid  acetoacetate  382090  dihydro-3-coumaric acid  glyceric acid  isothreonic acid  31263  nonadecanoic acid  354282  dihydroxymalonic acid  1912  glycine  cholesterol  1380  1862  butylamine  3-hydroxypropionic acid  5-hydroxymethyl-2-furoic acid  46307  46362  100321  104131  110018  119054  120562  135613  376650  377697  382585  384894  391739  391842  391853  397709  4716  dehydroascorbic acid  21725  myristic acid  4930  26741  109435  209171  345404  378013  behenic acid  33006  glucoheptulose  4577  5244  41833  41989  adipic acid  68  17651  408463  342736  4550  217691  136398  110343  390143  fucose  210312  azelaic acid  390137  palmitic acid  412430  384906  4541  365431  356984  401124  pimelic acid  31357  6-deoxyglucose  31563  4534  2-hydroxyglutaric acid  2039  342919  329435  16872  210249  187846  382905  209679  111399  370189  uridine  7458  18157  deoxypentitol  390188  390549  20902  209167  100107  100248  171020  64742  18197  21502  391855  glycerol-3-galactoside  5-hydroxynorvaline  34183  7876  4-aminophenol  114256  401723  xylulose  dehydroabietic acid  46377  168861  407455  22969  97456  guanosine  371262  2193  18176  pseudo uridine  210803  161875  cholic acid  231137  inositol-4-monophosphate  344959  sucrose  231695  5691  46128  367353  methionine sulfoxide  sinigrin  421453  montanic acid  1799  390140  108834  347424  cystine  356347  97870  327545  100841  391858  110873  370230  3-hydroxy-3-methylglutaric acid  133264  3204  phenylethylamine  379374  phosphate  4600  356926  2-deoxypentitol  131558  52863  211988  223087  346241  413693  guanidinosuccinate  133605  43100  malic acid  threonic acid  4879  592  390194  acetaminophen  11831  53486  356941  ribitol  395912  ribose  xylose  4746  85651  371557  arachidonic acid  threose  118869  210653  346561  135260  347190  cysteine  370223  390156  135615  341970  4723  98  erythrose  4548  31601  ethanolamine  380462  3029  26755  27145  223106  215853  cis-gondoic acid  maleimide  391740  168  120665  231827  390185  2821  tocopherol beta  phosphoethanolamine  214112  357377  2-ketoadipic acid  241684  110022  4721  N-acetylmannosamine  133590  364462  405512  47235  95440  lactic acid  86075  249248  26713  228052  asparagine  20282  14736  105141  3-phenyllactic acid  119066  pentose  376648  419123  48608  342924  glucose  390192  erythronic acid  100017  100036  arabitol  109386  erythronic acid lactone  17225  54  367427  46292  170278  351271  97455  11481  18407  100897  109990  26736  84185  41924  170069  224095  217920  355607  17297  beta-glycerolphosphate  360226  alanine  aminomalonate  12444  128767  lyxose  168874  377304  indole-3-lactate  cerotinic acid  uric acid  46143  161365  pantothenic acid  N-acetylaspartic acid  deoxycholic acid  223203  352123  beta-alanine  112501  trehalose  16747  131144  390198  346884  2-hydroxyvaleric acid  41866  237707  365909  380514  390131  112530  31547  388180  2,3-dihydroxybutanoic acid  4533  782  5990  thymine  1,2,4-benzenetriol  97834  kynurenine  390144  100556  91  methionine  glycerol-alpha-phosphate  2-monopalmitin  4983  130640  250586  230854  oleamide  34113  pyruvic acid  trans-4-hydroxyproline  390179  130716  oxoproline  1715  N-acetyl-D-tryptophan  maltotriose  108825  390218  8270  34149  itaconic acid  110805  34014  106623  xylitol  N-acetylglycine  133780  210714  glutamic acid  salicylic acid  134  4605  341715  17267  377751  213180  382599  17664  395982  aconitic acid  threitol  6324  5-hydroxy-3-indoleacetic acid  glutamine  379924  34163  tagatose  87822  41683  126391  188569  quinic acid  putrescine  linoleic acid  glucose-1-phosphate  125830  107960  galactinol  384956  379063  22735  fructose  palmitoleic acid  119025  371571  2-aminobutyric acid  1771  3-hydroxybutyric acid  377103  190066  110612  89050  360154  2543  2551  351222  3,6-anhydro-D-galactose  384938  110021  133434  31695  404192  171967  119129  citric acid  methanolphosphate  6376  88993  1981  329772  proline  1996  61  18173  349293  5259  380761  384978  109464  31559  320847  224096  390155  hippuric acid  threonine  321077  32158  376949  conduritol-beta-epoxide  390135  erythritol  lactamide  tocopherol alpha-  pinitol  41996  98022  610256  573  5837  160  4877  84213  253093  isopropylbenzene  17958  345452  413543  458  112264  89045  4543  100343  357374  valine  34081  glycerol  161085  210313  sorbitol  urea  myo-inositol  1684  110008  408450  undecanoic acid  tocopherol gamma-  892  21683  1690  20903  100326  100876  3232  3-aminoisobutyric acid  171564  357409  5-aminovaleric acid  18386  xylonic acid  307  110355  112544  228818  372453  citrulline  88994  oleic acid  377384  creatinine  97687  210697  85113  5-methoxytryptamine  indole-3-propionic acid  223080  88981  7490  31223  44509  4529  234947  390122  serine  18488  isocitric acid  3-(4-hydroxyphenyl)propionic acid  376823  cellobiose  lactose  33142  8240  41682  359963  104394  391864  tyrosine  levoglucosan  piperidone  121468  391859  9489  247758  188923  121467  228119  127847  34110  4929  209677  tryptophan  lactulose  94582  saccharic acid  413572  210239  4219  34097  34089  17947  mannitol  33282  88992  UDP-glucuronic acid  leucine  353342  2-deoxytetronic acid  1673  210231  isoleucine  6646  386801  mannose  110258  butane-2,3-diol  7543  3173  119023  135253  serotonin  2044  2-ketoisocaproic acid  2-hydroxybutanoic acid  glucuronic acid  210343  365939  31764  lysine  356925  330287  2,8-dihydroxyquinoline  phenylalanine  127277  indole-3-acetate  2-ketobutyric acid  maltose  11353  189936  210272  alpha-ketoglutarate  ornithine  4546  110321  120744  110328  xanthine  34007  2-deoxyerythritol  131421  ribonic acid  gluconic acid  inosine  161876  pipecolinic acid  210790  phenol  3294  110881  395262  hypoxanthine  389434  5884  histidine | 0.000105361  0.000282601  0.000282601  0.000282601  0.000282601  0.000282601  0.000521281  0.000554099  0.000554099  0.000554099  0.000554099  0.000554099  0.000765706  0.000765706  0.000765706  0.000765706  0.000833515  0.001003526  0.001003526  0.001003526  0.001003526  0.001114177  0.001141322  0.001165942  0.001165942  0.001165942  0.001165942  0.001165942  0.001165942  0.001165942  0.001165942  0.001165942  0.001165942  0.001165942  0.001165942  0.001165942  0.001165942  0.001165942  0.001165942  0.001165942  0.001165942  0.001165942  0.001165942  0.001165942  0.001165942  0.001165942  0.001232826  0.001235292  0.001276495  0.001313131  0.001313131  0.001313131  0.001313131  0.001407231  0.001407231  0.001480497  0.001480872  0.001496031  0.001580763  0.001580763  0.00158372  0.00158372  0.00158372  0.001600719  0.001601872  0.001602001  0.001602001  0.001606667  0.001624606  0.001624606  0.001822246  0.001922532  0.002008934  0.00204963  0.00204963  0.00204963  0.002071626  0.002193469  0.002307234  0.002348301  0.002468155  0.002475548  0.002492585  0.00255089  0.002592936  0.002634201  0.002718729  0.002974296  0.003086444  0.003127602  0.003127602  0.003562416  0.003576984  0.003622517  0.003905498  0.004109759  0.004218542  0.004269499  0.004457256  0.004544569  0.004640213  0.004762702  0.004762702  0.004762702  0.004762702  0.004762702  0.005207852  0.005319391  0.005609992  0.006346882  0.006364971  0.00653475  0.006587083  0.006587083  0.006587083  0.006984569  0.007014133  0.007018402  0.007277086  0.007990126  0.008098608  0.008226945  0.008440299  0.008440299  0.008509762  0.008759728  0.008788887  0.00903249  0.009043565  0.00920018  0.00920018  0.009419596  0.009774594  0.009783085  0.009905169  0.00992816  0.010295827  0.010312853  0.01047399  0.010623551  0.011170924  0.011597692  0.011972625  0.011972625  0.011972625  0.011972625  0.012932203  0.012932203  0.012979222  0.013025977  0.013025977  0.01384711  0.014170641  0.015005373  0.015152282  0.015299582  0.015299582  0.016938879  0.016938879  0.018112427  0.018372982  0.018824223  0.019279998  0.020453289  0.02129615  0.02129615  0.02242327  0.023227155  0.023227155  0.023908592  0.023908592  0.024210945  0.024210945  0.024210945  0.024223802  0.024223802  0.024954709  0.025716034  0.02658707  0.026720906  0.028273268  0.028273268  0.029301651  0.029301651  0.030346614  0.031267137  0.031267137  0.031267137  0.031316733  0.032229382  0.032665119  0.03315779  0.03315779  0.03315779  0.034390713  0.034716661  0.034716661  0.035213917  0.035213917  0.035338733  0.036076662  0.036594426  0.036705178  0.036816495  0.037254283  0.038888276  0.039059941  0.039288186  0.039629611  0.039629611  0.039826234  0.039826234  0.039826234  0.039826234  0.040951633  0.041036657  0.041036657  0.041036657  0.041807402  0.041820775  0.042326176  0.042707625  0.042825643  0.043213579  0.043213579  0.043213579  0.043633113  0.043633113  0.044015566  0.04428499  0.04428499  0.045222179  0.045424272  0.046218402  0.048583165  0.049870262  0.049870262  0.049870262  0.049870262  0.053117523  0.056931609  0.057605637  0.060158106  0.060271963  0.06156913  0.06156913  0.063923523  0.063923523  0.064408048  0.066048892  0.066048892  0.066048892  0.066048892  0.066048892  0.066048892  0.066601232  0.067020042  0.067159037  0.068148803  0.068977379  0.06899593  0.06994491  0.070328355  0.073588923  0.074380813  0.074380813  0.075679439  0.077476055  0.077492803  0.077572446  0.078079533  0.078484332  0.081150854  0.081150854  0.081150854  0.081150854  0.081387795  0.082177081  0.082972802  0.082972802  0.082972802  0.086369198  0.086576935  0.087833364  0.087833364  0.087833364  0.087940089  0.088525471  0.088637908  0.088637908  0.089457616  0.089840748  0.089840748  0.089840748  0.090808995  0.091182446  0.09168384  0.092024125  0.092574927  0.093892371  0.097176301  0.098070374  0.099631525  0.101745912  0.103434817  0.104200333  0.104386482  0.104515211  0.105478554  0.110635343  0.111315399  0.114306124  0.114306124  0.114306124  0.118682484  0.119607935  0.12085347  0.122129858  0.126171258  0.127998542  0.128346775  0.129276931  0.134616823  0.140099599  0.142228495  0.142228495  0.142228495  0.142228495  0.142652892  0.145141964  0.147358752  0.147358752  0.147358752  0.147358752  0.152778326  0.154192638  0.155939307  0.15877908  0.161373359  0.16239889  0.16239889  0.165593431  0.169715063  0.176887416  0.179717988  0.181508601  0.185935043  0.185935043  0.187776017  0.188918317  0.191732426  0.192983091  0.197303041  0.198576571  0.198576571  0.198892569  0.202290689  0.206183611  0.20779959  0.208428617  0.216654334  0.216963395  0.216963395  0.216963395  0.217899301  0.218692127  0.218955352  0.221370581  0.222390962  0.222390962  0.223689131  0.223689131  0.223689131  0.223689131  0.223689131  0.223689131  0.224565825  0.227359852  0.230222262  0.23138963  0.23138963  0.23138963  0.232751848  0.232751848  0.23379949  0.235246235  0.235694329  0.236207235  0.237085879  0.249244769  0.251785569  0.256697465  0.256697465  0.256697465  0.261392178  0.261392178  0.265869915  0.267915566  0.268417643  0.269506275  0.274756574  0.274929736  0.283716109  0.289052525  0.289367312  0.290444049  0.290444049  0.290896973  0.295855599  0.301562779  0.303117776  0.305260167  0.305722238  0.306695898  0.3079703  0.309633353  0.309633353  0.309633353  0.31333854  0.323042752  0.323042752  0.324842435  0.330431958  0.33836408  0.33906977  0.33906977  0.351877017  0.355212538  0.355212538  0.356368757  0.357359441  0.359415994  0.361249201  0.364325254  0.369149457  0.370127627  0.370735749  0.371315184  0.373567255  0.374084223  0.383053804  0.383833198  0.383833198  0.385376234  0.385433958  0.392369258  0.395310212  0.399077977  0.403814909  0.403814909  0.422667934  0.422667934  0.423525104  0.423525104  0.425178086  0.432068808  0.432068808  0.437429942  0.441166477  0.44311944  0.445493563  0.445810477  0.449546685  0.465824628  0.466643998  0.468412147  0.468583153  0.468583153  0.468583153  0.4685944  0.4685944  0.470048168  0.473803325  0.473890561  0.483078895  0.487583486  0.487947113  0.489112364  0.4922819  0.501540478  0.519096372  0.519096372  0.519379236  0.520415664  0.520415664  0.520415664  0.520415664  0.53829317  0.538859121  0.546529481  0.546688236  0.546688236  0.546688236  0.546688236  0.546688236  0.555128264  0.555128264  0.56349334  0.567819262  0.575664429  0.575664429  0.58060173  0.586650643  0.588650892  0.589820021  0.589820021  0.601776347  0.608485446  0.608485446  0.608485446  0.612345783  0.620303038  0.621294627  0.625554215  0.626499129  0.634684559  0.634986922  0.634986922  0.637292648  0.637292648  0.64359757  0.647904069  0.647904069  0.647904069  0.658090635  0.65935456  0.660199446  0.668204771  0.672062682  0.676232648  0.676232648  0.677680595  0.684623377  0.699447652  0.701576016  0.704158346  0.707266313  0.716971252  0.71964485  0.730120615  0.747615448  0.756423859  0.756423859  0.757717373  0.780366308  0.785371791  0.790785295  0.790785295  0.793777908  0.799465555  0.802144879  0.802144879  0.807924291  0.826418774  0.834178644  0.834178644  0.854290733  0.856717363  0.862752779  0.865887485  0.865887485  0.870730459  0.872457529  0.880458357  0.883298848  0.883298848  0.889592692  0.889592692  0.889592692  0.889592692  0.895466261  0.900729325  0.901374333  0.902705084  0.905928162  0.907161632  0.91351394  0.924218169  0.929434082  0.931221931  0.936688725  0.940589121  0.940589121  0.947138023  0.947138023  0.965292577  0.965292577  0.965292577  0.965292577  0.965339332  0.965339332  0.965339332  0.965339332  0.965339332  0.966592495  0.972400919  0.97478675  0.976297091  0.980607524  0.98152063  0.98152063  0.982968332  0.982968332  0.985815417  0.986838206  0.988634796  0.988646712  0.988646712  0.988646712  0.993608135  0.998109507  0.99824007  0.99824007 | 3.062615037  3.089882968  2.567027974  2.437928369  3.509047853  4.082715315  2.861427843  3.126471343  3.543464503  2.792252675  4.028711668  3.252990084  3.513451639  4.262662817  3.070641035  2.966688284  4.853230551  4.976479333  3.54803916  2.972476864  3.02895446  2.771825047  3.081856135  4.526623141  3.917119948  3.496750789  4.183963454  3.014724164  3.912365245  2.843966263  2.442727544  2.690427376  2.357141651  3.229183573  3.361198761  3.340372774  3.236508759  2.829396155  3.508528215  3.675603471  3.358846269  2.933052958  1.805256682  3.236508759  2.926786537  2.799208367  2.786713066  4.436461324  2.015572567  3.648830227  3.56814536  1.791394987  3.119421512  7.086521748  2.909164367  2.95678042  3.399294736  3.055761099  2.594792418  2.156077968  4.414533401  3.472963649  3.306650629  3.682364402  4.557266172  2.913462585  4.43960213  2.978626135  3.055812077  3.884628717  2.791998904  2.999584032  3.147051962  3.963352176  2.827226391  3.032267351  2.231464135  4.655782639  3.701408645  3.535421205  3.696430437  1.781222237  3.31155637  1.682915384  3.269060979  2.384146246  4.350002544  3.404764893  2.429524263  3.00710033  3.508895602  1.81361632  2.349479478  2.165157366  3.521661669  2.644908306  3.007963037  2.261463537  3.574668026  1.966887584  1.807974647  3.074505134  4.294029196  3.870240618  2.925278907  3.390730766  3.669852582  2.571908271  2.206571649  2.36967867  1.676941485  2.201690075  4.026624452  1.698920975  3.825221717  3.508803617  3.293817816  2.548405922  2.694805948  3.220341842  3.792405895  2.950570092  3.501557998  2.797160586  2.918160143  2.267575135  3.665604423  3.492719999  3.0823905  1.698395002  1.935498528  3.947677726  3.710140719  3.761446143  2.05896874  2.138537459  2.048987001  2.50916019  1.952896755  3.681744129  3.065554683  1.771732711  3.324962057  2.563244777  3.286657926  3.142762266  2.263396571  2.249550795  2.860904212  2.482695745  3.341257906  1.967459168  1.73856125  2.561607735  1.689385127  1.828303918  1.686420681  4.401563619  3.24671713  3.21895647  2.524055601  2.703570257  2.332153423  2.710272702  2.597428622  3.827861949  3.565945875  1.719041106  2.375833015  3.530664195  3.11001635  3.060885943  3.129381556  2.276745906  3.139168609  3.309799919  1.779837752  3.350656124  2.504582776  2.724595474  5.339062661  1.937638776  2.550710382  2.550710382  3.856395253  1.985113175  2.64717724  5.234747092  4.307640575  2.937464646  3.918168902  4.560441367  3.401979853  2.051918057  3.446370701  2.654362422  3.1436004  2.419496144  3.770164607  3.790577311  3.415767147  1.818549211  5.122109254  2.72214094  3.517902449  3.116305384  2.763364071  3.775862449  2.217971447  3.117698863  2.114988586  1.80402312  5.207077791  2.903355242  3.661145214  2.760103545  2.633610166  3.191308396  2.616358205  1.657975524  2.903355242  2.079198096  3.800753361  1.807960135  1.728180238  2.694756462  2.797438444  2.031633969  3.187662218  2.112241568  2.000248373  4.091649259  2.616474194  2.97932397  3.837896524  3.586415184  3.466241569  1.590027478  3.814800729  1.966460036  2.177799075  5.551552251  3.066368315  1.808309246  3.3249159  3.531957861  3.625058001  3.474175829  2.379938138  2.556728851  2.184712498  3.396118875  3.975717456  3.976225486  3.827706773  1.788142993  2.824225033  4.723143251  3.458053177  2.647759485  3.763348703  2.994049439  5.504358037  3.324466719  3.471890071  2.854088285  2.807686682  1.561169311  2.030552026  2.554556251  2.202383648  2.128432228  2.029423864  3.027637289  3.494059313  1.606266459  1.654309674  2.443945111  2.858689365  4.509947087  2.411075562  2.781069804  1.771324213  3.742127557  4.222411544  1.854843575  4.989377903  2.34173532  3.381612376  2.50324367  3.714158637  2.64038739  3.027282023  1.965756061  2.420957603  2.782182908  2.931673579  3.187603833  2.335280305  3.305392045  1.80535494  3.004843411  2.767297214  1.876844229  2.236633141  2.681232341  3.114288899  2.750408557  4.645553133  3.480776457  4.347501153  2.921649861  3.084462957  1.572117808  4.617211668  1.710127705  1.990994415  3.631253664  2.697389017  3.057338243  1.692617267  3.365292734  2.603258538  2.235065978  2.954092043  2.181645535  1.852852913  3.367051997  2.59428093  4.412824157  3.920905943  3.225184644  4.272615535  1.798119586  1.781286083  1.983339734  3.378265188  2.874518068  2.811675698  4.549111871  2.766501701  1.850716147  3.16125582  5.589100695  4.030347524  1.680423615  1.769475342  2.113495895  4.164403147  1.964506083  2.418266288  3.622881397  2.184106914  2.723172571  1.153184721  3.485264716  2.669131919  1.783774916  4.291002082  4.341975552  2.586895344  3.032636272  1.863255172  1.676646703  2.633415005  2.304758972  2.126390551  3.054416437  4.079536727  3.037287624  3.446944537  4.098734893  2.39695512  2.624329493  5.810276446  4.316797865  2.854234536  3.758413569  3.826902158  2.92122945  4.0937047  2.441259917  2.841984473  3.261485475  1.931230815  4.037370251  2.436744355  2.055688343  2.519047016  2.776967306  2.637000564  0.375952866  5.544197191  2.210513228  2.267061039  3.336734094  4.12095016  3.070303304  4.216478677  3.174327399  2.213572892  2.894321264  2.061697974  4.829820432  2.071974895  3.180899588  3.021607963  2.210121391  4.057882895  1.875600221  2.36702827  3.025219674  2.49271669  3.916216372  2.602699926  5.726602689  1.834036005  2.553642487  2.062278023  3.928183909  2.781399771  3.004360261  3.566310915  3.197462727  2.178744569  3.296484794  1.700810957  2.851882768  2.288080307  3.29182791  2.220058984  2.977374766  1.64264987  2.220058984  4.22454621  4.49805364  3.368632022  2.965752968  2.890956389  2.888604045  2.50743982  5.121574566  2.993852132  1.762182921  2.007529932  2.631982094  2.009091747  3.599386201  2.208132468  3.609206979  2.670352624  2.820552314  3.032881342  1.631907386  3.239699568  2.730478592  3.026574304  2.591356003  1.519160269  2.002471451  2.412002017  4.120235212  3.428751387  1.658027445  4.791963766  2.496758384  4.982032231  4.053237198  3.525455017  5.127815607  6.296572482  5.979830097  2.288585155  1.747611988  4.402983502  3.741194047  1.923179142  4.517955717  2.168433288  3.117429866  3.146038098  3.013675634  2.050708108  2.47926555  1.628157517  4.485842119  3.154626444  2.499238541  3.416533853  1.741685334  4.292413289  2.512537866  1.673317034  2.315297616  4.292413289  3.826692711  1.818916121  2.401056032  3.845855657  5.265347387  4.909041044  2.562465353  2.786096234  1.701165933  2.922310792  3.017722782  1.841702065  2.403666048  1.844047509  2.143022697  3.292481985  2.01056602  5.045050932  4.929036746  2.670067589  4.024295896  2.757670184  3.298261368  2.616513307  2.616513307  4.92578757  3.175517131  2.687797071  2.249806727  1.532457743  2.195661551  4.685075086  3.483881165  2.686667209  2.097576535  1.867043155  2.063364473  2.371804813  2.408609328  2.382425856  3.621822095  2.311868174  3.446643412  2.556241013  2.382099985  3.466029097  2.585871961  2.581803896  2.625241807  1.528230286  3.83572856  2.256011018  2.152775623  3.803644245  4.114334336  4.462625232  4.862660119  2.492088177  2.940336623  4.739371229  2.55770612  2.864625865  3.391391117  2.99559535  4.371880615  3.391057061  2.103734256  5.193925041  3.281527896  4.532362117  3.821084593  2.887012642  2.97911442  3.436683623  1.698372604  2.582421895  3.19610165  5.056319773  3.54272265  2.938134148  1.990682759  3.505480916  3.958713929  4.091004154  2.707716549  2.142245313  4.320118746  4.550333967  2.391436575  3.723296915  2.871077368  3.501780989  3.415408953  4.288964033  2.80223665  4.364078824  2.013286642  2.693180751  3.034181815  1.542634965  2.649118723  2.792139143  3.699925277  4.077304676  4.478241496  3.328555215  2.564176777  3.187556833  2.356587896  2.133374682  4.265895981  2.701021042  1.827015726  3.024033393  3.397483372  2.8157838  1.999062338  3.660706391 | 2.744184496  2.922488768  2.410861674  2.274591405  3.253476956  3.835157253  2.606937717  2.940717884  3.192264865  3.269447953  3.798653036  3.013873858  2.958832437  3.825515513  2.831474699  2.760628612  4.621076158  4.825844534  3.389890316  2.771346931  2.810543807  2.541583472  2.871011503  4.215844651  3.763098208  3.310038464  3.974796074  2.781506136  3.753289118  2.635430755  2.126379247  2.503166066  3.012445217  3.046472736  3.178231836  3.224074887  3.062163189  2.653326623  3.258306792  3.514400167  3.172208743  2.751342595  1.545610965  3.062163189  2.738023298  2.603942292  2.650717076  4.80555678  2.374155102  3.479687186  3.383349752  1.546740566  3.34486843  6.895802869  2.730812038  2.672670603  3.223189249  2.690764449  2.392746608  1.917527783  3.966371427  3.264793361  3.729126761  3.504796958  4.112227553  2.734078232  3.998417038  3.374835834  2.823645283  3.741623871  3.186379498  2.873792344  2.835094441  3.814933428  3.199407482  2.89175242  2.048931881  4.485861788  3.535308421  3.445848777  3.920819689  1.497779475  3.143886955  1.37268915  3.086123012  2.109800518  4.206721371  3.227137253  2.230796253  2.8210587  3.278180366  1.606919952  2.100749019  2.486358995  3.430675278  2.194411736  2.903633026  2.076596119  3.24643612  1.692646018  1.593533292  2.92338233  4.050499087  3.73748101  2.694497467  3.20463535  3.50863523  2.426078256  2.613887126  2.702574438  1.415379538  1.902740522  3.767507169  1.501287047  3.690856702  3.414654983  3.02767978  2.342910306  2.490642923  2.838022201  3.576120474  2.811843669  3.42974206  2.534376387  2.725696294  2.06847692  3.527181171  3.308627959  2.962489194  1.458240727  1.721335502  3.83997287  3.527600415  3.658568223  1.861933604  1.951458693  1.774129373  2.322780923  1.747130838  3.533195519  2.68969056  1.595250423  3.122988585  2.365822029  3.042456489  2.937560536  2.123494308  1.988591784  2.664744222  2.105081233  3.614308358  1.716457911  1.51856194  2.480480105  1.409553437  1.538218781  1.362845032  4.186047689  3.060777256  3.10861135  1.868604584  2.549921902  2.184769428  2.519944275  2.413860763  3.663431373  3.421283  1.443759174  2.217355766  3.440631964  3.004424688  2.599810823  2.912046283  2.079800634  2.992524988  2.916223156  1.598818688  3.559533465  2.113471893  2.532422877  5.082924779  1.711745491  2.428562134  2.428562134  3.017594177  1.743491102  2.459640303  5.148192575  4.213104396  2.832912935  3.86092995  4.465238341  3.299969853  1.798795759  3.362448754  2.48570729  2.938224856  2.273669561  3.627831333  3.648168719  3.323475849  1.684254838  4.199127447  2.545887905  3.351105254  2.245328866  2.64647278  3.605168106  2.093447056  2.948713451  1.929849314  1.611326494  5.117767149  2.74858154  3.482728062  2.66934089  2.463995132  3.079195876  2.473297799  1.444840482  2.751342595  1.93334481  3.489293411  1.592376423  1.551733165  2.52476043  2.65911177  1.771939721  3.051245487  1.94118807  1.755195561  4.268187962  2.475803729  2.864203452  3.675863971  3.488338873  3.3015912  1.418793675  3.652260145  1.610696676  2.006166475  5.855516602  2.967746726  1.663977108  3.156855049  3.447122164  3.508093923  3.347322565  2.641679598  2.415001593  2.060561799  3.265443214  3.735265162  3.870004679  3.682209697  1.59702023  2.744739034  4.81108646  3.381330975  2.951743445  3.65542047  2.864461701  5.402494869  3.24767549  3.678786999  2.767877561  2.662814256  1.393320064  1.923891783  2.433892525  2.07173853  1.976376572  1.923891783  2.901119306  3.305358488  1.412674172  1.393522627  2.563322395  2.685023135  4.383777004  2.194358918  2.605522563  1.633080521  3.523883471  4.12940696  1.701604234  5.090117231  2.239149813  3.29892207  2.274789571  3.529609815  2.447445547  2.894736243  1.859091218  2.302601925  3.017750835  2.791703563  3.12901412  2.481851783  3.151778016  1.614879574  2.898248466  1.945203547  1.739286381  2.142773826  2.52796275  2.949420217  2.585039914  4.559557938  3.320014582  4.27638948  2.721091922  3.704671314  1.271934356  4.739399462  1.514866739  1.810285281  3.484035834  2.571970121  2.967676467  1.450436435  3.610254298  2.535385125  2.147756685  2.812021639  1.822124297  1.580469426  3.60634332  2.69400805  4.304984602  3.84004691  3.13882523  4.08725452  1.629274095  1.655230436  1.830091452  3.270394524  2.785651704  2.700064975  4.667566026  2.682708394  1.972212461  3.075179577  5.536422566  3.974832344  1.538461625  1.665167603  1.958929671  4.213332764  1.879138694  2.285686918  3.510476874  2.081278933  2.65309843  0.432888786  3.438550152  2.570082571  1.683820044  4.466047915  4.292041872  2.434751716  2.776637517  1.690889024  1.544176371  2.549745679  2.090037725  1.884570372  2.961478799  3.93066494  2.956422514  3.405790713  4.006179586  2.307664384  2.539586407  5.768646003  4.221652048  2.762309405  3.579957749  3.723677836  2.675550735  3.949819459  2.213780575  3.043617262  3.175648694  1.851488785  3.998695208  2.525232874  1.724387053  2.460536044  2.515411684  2.555757065  0  5.623213223  2.090878974  2.196708177  3.278045222  4.206647197  2.990635852  4.025985255  2.990455351  2.103853421  2.663788867  2.281612531  4.682209143  1.929876829  3.049559572  2.945990435  2.115873076  4.152401698  1.618075005  2.292623151  2.958673118  2.413541927  4.127632667  2.868416749  5.778278489  1.730908809  2.654861408  1.955610877  3.876077376  2.625406497  3.130685479  3.493967857  3.129935086  2.368857599  3.061465405  1.602516558  2.758600811  2.396331025  3.181999965  2.36169009  2.881967723  1.528885638  2.358385814  4.281504568  4.439526224  3.448934176  2.888918296  2.999832329  2.988652815  2.44850395  5.0820436  2.957908344  1.672173257  1.89361671  2.526198987  1.781779069  3.563832134  2.020813505  3.556583499  2.597020484  2.724616443  2.955830117  1.508274628  3.315526338  2.892856305  2.8974253  2.675083043  1.864468009  1.914319658  2.469461787  4.229557303  3.288991201  1.587792997  4.836217078  2.585979407  4.942400737  3.99078329  3.494267373  5.189632614  6.22711022  5.936325706  2.240876898  1.661696038  4.345261773  3.828294029  1.839049653  4.427684178  2.094045091  3.086713763  3.194300192  3.073670375  1.932044012  2.431315984  1.555508827  4.409384746  3.052384464  2.377005088  3.378946376  1.84787042  4.322897375  2.348446352  1.565084639  2.342786976  4.322897375  3.784562445  1.766111252  2.324582703  3.774389433  5.234798448  4.87318768  2.619550614  2.75624498  1.757799993  2.748988468  3.091091041  1.770233184  2.46473503  1.903774018  2.073925349  3.064779265  1.835378256  5.072259537  4.955956424  2.641208527  4.046264694  2.694376763  3.268963224  2.651687008  2.651687008  4.961693673  3.126887402  2.661514144  2.300108243  1.443225714  2.167983877  4.712559231  3.41720252  2.619106624  2.13780576  1.843738131  2.014006949  2.300666057  2.378266807  2.435235623  3.587155099  2.279918724  3.420458365  2.597999658  2.421524152  3.438337791  2.608245368  2.601407262  2.605839239  1.473233074  3.818171896  2.277240222  2.132421535  3.751699222  4.139441807  4.476231143  4.888071536  2.51112818  2.900642509  4.757631487  2.53695863  2.845300345  3.423413172  2.977869387  4.383237417  3.373561135  2.065216069  5.204520417  3.298155531  4.592335858  3.809919354  2.893471721  2.968593971  3.461556744  1.679484275  2.573883061  3.215398451  5.069614242  3.552400563  2.974876372  1.980944021  3.512785895  3.964015278  4.085388467  2.701346743  2.150771182  4.313468261  4.585447419  2.381676143  3.716888052  2.865215571  3.497270558  3.410479145  4.284843188  2.805596695  4.36027594  1.987235558  2.688312463  3.011673993  1.553314951  2.645639232  2.795630278  3.705565578  4.069206142  4.480910721  3.326483914  2.565391838  3.184703355  2.35305941  2.127491482  4.264381631  2.702152935  1.831439858  3.026445881  3.396853858  2.816371842  1.998582987  3.660801803 |

**Table S5:** Table of identified metabolites and the associated MS percentages in CSF and Serum reported by BinBase name, BinBase identifier, and KEGG identifier.

| **BinBase name** | **BB id** | **KEGG** | **CSF** | **in Serum** |
| --- | --- | --- | --- | --- |
| xylulose | 31632 | C00312 | 57% | 63% |
| xylose | 169 | C00181 | 77% | 73% |
| xylonic acid | 3470 | C00502 | 13% | 3% |
| xylitol | 5857 | C00379 | 83% | 53% |
| xanthine | 1669 | C00385 | 37% | 10% |
| valine | 3 | C00183 | 60% | 93% |
| uridine | 4705 | C00299 | 0% | 30% |
| uric acid | 33210 | C00366 | 70% | 100% |
| urea | 3256 | C00086 | 83% | 83% |
| undecanoic acid | 412761 | C17715 | 60% | 100% |
| UDP-glucuronic acid | 21705 | C00167 | 50% | 63% |
| tyrosine | 16 | C00082 | 100% | 100% |
| tryptophan | 14 | C00078 | 97% | 97% |
| trehalose | 111823 | C01083 | 40% | 20% |
| trans-4-hydroxyproline | 43180 | C01157 | 0% | 100% |
| tocopherol gamma- | 4545 | C02483 | 0% | 97% |
| tocopherol beta | 42194 | C14152 | 0% | 63% |
| tocopherol alpha- | 100 | C00376 | 0% | 100% |
| thymine | 1692 | C00178 | 0% | 40% |
| threose | 100335 | C06463 | 43% | 3% |
| threonine | 171298 | C00188 | 100% | 100% |
| threonic acid | 172 | C01620 | 90% | 100% |
| threitol | 770 | C16884 | 100% | 100% |
| tagatose | 4833 | C00795 | 7% | 33% |
| sucrose | 16661 | C00089 | 37% | 100% |
| sorbitol | 162 | C00794 | 83% | 47% |
| sinigrin | 351512 |  | 43% | 0% |
| serotonin | 364413 | C00780 | 0% | 70% |
| serine | 25 | C00065 | 100% | 100% |
| salicylic acid | 3063 | C00805 | 13% | 83% |
| saccharic acid | 11214 | C00818 | 53% | 13% |
| ribose | 384948 |  | 90% | 87% |
| ribonic acid | 1683 | C01685 | 100% | 83% |
| ribitol | 7362 | C00474 | 100% | 60% |
| quinic acid | 16691 | C00296 | 20% | 40% |
| pyruvic acid | 583 | C00022 | 80% | 37% |
| putrescine | 21703 | C00138 | 100% | 40% |
| pseudo uridine | 1688 | C02067 | 100% | 100% |
| proline | 171970 | C00148 | 83% | 100% |
| piperidone | 34023 |  | 30% | 0% |
| pipecolinic acid | 2448 |  | 0% | 83% |
| pinitol | 115876 |  | 0% | 20% |
| pimelic acid | 33429 | C02656 | 100% | 97% |
| phosphoethanolamine | 1723 | C00346 | 77% | 97% |
| phosphate | 4 | C00009 | 63% | 97% |
| phenylethylamine | 2005 | C05332 | 0% | 70% |
| phenylalanine | 33 | C00079 | 100% | 100% |
| phenol | 31889 | C00146 | 3% | 17% |
| pentose | 360841 |  | 27% | 17% |
| pantothenic acid | 31356 | C12276 | 10% | 3% |
| palmitoleic acid | 391898 | C08362 | 0% | 93% |
| palmitic acid | 11 | C00249 | 97% | 100% |
| oxoproline | 10 | C01879 | 100% | 100% |
| ornithine | 1821 | C00077 | 100% | 100% |
| oleic acid | 1677 | C00712 | 13% | 77% |
| oleamide | 20961 | C19670 | 93% | 87% |
| nonadecanoic acid | 46258 | C16535 | 13% | 27% |
| N-acetylmannosamine | 14817 | C00645 | 100% | 20% |
| N-acetylglycine | 97747 |  | 17% | 7% |
| N-acetylaspartic acid | 4081 | C01042 | 10% | 0% |
| N-acetyl-D-tryptophan | 131996 | lactamide | 0% | 87% |
| myristic acid | 127 | C06424 | 90% | 100% |
| myo-inositol | 1741 | C00137 | 100% | 93% |
| montanic acid | 84087 |  | 20% | 3% |
| methionine sulfoxide | 372461 | C15998 | 13% | 63% |
| methionine | 45 | C00073 | 100% | 100% |
| methanolphosphate | 5393 |  | 0% | 100% |
| mannose | 390222 | C00159 | 83% | 100% |
| mannitol | 17883 | C00392 | 67% | 20% |
| maltotriose | 84521 | C01835 | 0% | 30% |
| maltose | 1979 | C00208 | 20% | 83% |
| malic acid | 1391 | C00711 | 100% | 90% |
| maleimide | 1743 | C07272 | 0% | 10% |
| lyxose | 85112 | C00476 | 13% | 0% |
| lysine | 12 | C00047 | 100% | 100% |
| linoleic acid | 165 | C01595 | 0% | 100% |
| levoglucosan | 3169 |  | 100% | 100% |
| leucine | 171969 | C00123 | 100% | 93% |
| lactulose | 6432 | C07064 | 60% | 63% |
| lactose | 104901 | C01970 | 0% | 10% |
| lactic acid | 46123 | C01432 | 63% | 63% |
| lactamide | 341711 |  | 57% | 20% |
| kynurenine | 210712 | C01718 | 0% | 87% |
| itaconic acid | 101725 | C00490 | 60% | 0% |
| isothreonic acid | 1679 | C00639 | 100% | 100% |
| isopropylbenzene | 355471 | C14396 | 0% | 10% |
| isoleucine | 15 | C00407 | 100% | 100% |
| isocitric acid | 12267 | C00451 | 100% | 10% |
| inositol-4-monophosphate | 45351 | C03546 | 3% | 20% |
| inosine | 84524 | C00294 | 90% | 100% |
| indole-3-propionic acid | 112556 |  | 0% | 90% |
| indole-3-lactate | 390397 |  | 0% | 100% |
| indole-3-acetate | 69 | C00954 | 0% | 100% |
| hypoxanthine | 1663 | C00262 | 100% | 77% |
| hydroxycarbamate | 130396 |  | 60% | 60% |
| histidine | 6355 | C00135 | 0% | 70% |
| hippuric acid | 360150 | C01586 | 0% | 33% |
| guanosine | 1966 | C00387 | 0% | 93% |
| guanidinosuccinate | 16942 | C03139 | 0% | 27% |
| glycine | 6 | C00037 | 100% | 100% |
| glycerol-alpha-phosphate | 1687 | C03189 | 100% | 100% |
| glycerol-3-galactoside | 1693 | C05401 | 100% | 93% |
| glycerol | 30 | C00116 | 100% | 87% |
| glyceric acid | 394878 | C00258 | 100% | 100% |
| glutamine | 171972 | C00064 | 100% | 100% |
| glutamic acid | 28 | C00025 | 43% | 100% |
| glucuronic acid | 344793 | C00191 | 13% | 100% |
| glucose-1-phosphate | 3167 | C00103 | 27% | 57% |
| glucose | 76 | C00221 | 100% | 97% |
| gluconic acid | 7501 | C00800 | 97% | 100% |
| glucoheptulose | 3191 | C02076 | 33% | 0% |
| galactinol | 1982 | C01235 | 10% | 0% |
| fumaric acid | 1718 | C00122 | 100% | 90% |
| fucose | 3009 | C02095 | 90% | 60% |
| fructose | 50366 | C02336 | 100% | 100% |
| ethanolamine | 45341 | C00189 | 80% | 7% |
| erythrose | 102661 | C01796 | 93% | 60% |
| erythronic acid lactone | 3187 | C02341 | 47% | 0% |
| erythronic acid | 3171 |  | 80% | 7% |
| erythritol | 92 | C00503 | 100% | 100% |
| dihydroxymalonic acid | 14759 |  | 67% | 0% |
| dihydro-3-coumaric acid | 384891 | C11457 | 0% | 27% |
| deoxypentitol | 33037 |  | 100% | 13% |
| deoxycholic acid | 95409 | C04483 | 0% | 40% |
| dehydroascorbic acid | 3163 | C05422 | 33% | 0% |
| dehydroabietic acid | 251689 | C12078 | 40% | 60% |
| cystine | 94 | C01420 | 0% | 97% |
| cysteine | 65 | C00097 | 23% | 83% |
| creatinine | 31 | C00791 | 100% | 100% |
| conduritol-beta-epoxide | 2670 |  | 37% | 20% |
| citrulline | 1712 | C00327 | 100% | 97% |
| citric acid | 288 | C00158 | 100% | 100% |
| citramalic acid | 16826 | C00815 | 77% | 3% |
| cis-gondoic acid | 87783 | C16526 | 0% | 30% |
| cholic acid | 110403 | C05463 | 0% | 10% |
| cholesterol | 19 | C00187 | 100% | 100% |
| cerotinic acid | 17982 |  | 37% | 20% |
| cellobiose | 1373 | C01971 | 0% | 10% |
| butylamine | 1871 | C18706 | 30% | 0% |
| butane-2,3-diol | 485 | C03046 | 10% | 20% |
| beta-glycerolphosphate | 22021 | C02979 | 0% | 67% |
| beta-alanine | 148 | C00099 | 0% | 100% |
| behenic acid | 46315 | C08281 | 17% | 7% |
| azelaic acid | 329430 | C08261 | 100% | 100% |
| aspartic acid | 79 | C00049 | 17% | 97% |
| asparagine | 146 | C00152 | 73% | 93% |
| arachidonic acid | 6529 | C00219 | 0% | 100% |
| arabitol | 372446 | C01904 | 97% | 100% |
| aminomalonate | 413 | C00872 | 3% | 100% |
| alpha-ketoglutarate | 294 | C00026 | 0% | 47% |
| alanine | 18223 | C00041 | 80% | 93% |
| adipic acid | 125 | C06104 | 100% | 93% |
| aconitic acid | 29 | C00417 | 100% | 70% |
| acetoacetate | 87705 | C00164 | 10% | 0% |
| acetaminophen | 101023 | C06804 | 10% | 7% |
| 6-deoxyglucose | 342712 |  | 73% | 33% |
| 5-methoxytryptamine | 284 | C05659 | 0% | 30% |
| 5-hydroxynorvaline | 4757 |  | 3% | 17% |
| 5-hydroxymethyl-2-furoic acid | 31858 | C20448 | 0% | 10% |
| 5-hydroxy-3-indoleacetic acid | 31552 | C05635 | 47% | 0% |
| 5-aminovaleric acid | 416577 | C00431 | 27% | 27% |
| 4-aminophenol | 133583 |  | 27% | 27% |
| 3,6-anhydro-D-galactose | 102714 | C06474 | 0% | 23% |
| 3-phenyllactic acid | 113643 |  | 0% | 33% |
| 3-hydroxypropionic acid | 1977 | C01013 | 17% | 0% |
| 3-hydroxybutyric acid | 145501 | C01089 | 70% | 47% |
| 3-hydroxy-3-methylglutaric acid | 32000 | C03761 | 67% | 13% |
| 3-aminoisobutyric acid | 12265 | C05145 | 0% | 90% |
| 3-(4-hydroxyphenyl)propionic acid | 110341 | C01744 | 0% | 20% |
| 2,8-dihydroxyquinoline | 104444 | C06342 | 0% | 30% |
| 2,3-dihydroxybutanoic acid | 31743 |  | 100% | 100% |
| 2-monopalmitin | 2080 |  | 3% | 93% |
| 2-ketoisocaproic acid | 208 | C00233 | 83% | 100% |
| 2-ketobutyric acid | 105066 | C00109 | 13% | 3% |
| 2-ketoadipic acid | 118816 | C00322 | 10% | 70% |
| 2-hydroxyvaleric acid | 25922 |  | 100% | 13% |
| 2-hydroxyglutaric acid | 2000 | C02630 | 13% | 97% |
| 2-hydroxybutanoic acid | 40 | C05984 | 100% | 57% |
| 2-deoxytetronic acid | 1208 |  | 10% | 3% |
| 2-deoxypentitol | 21714 |  | 50% | 17% |
| 2-deoxyerythritol | 34100 |  | 37% | 23% |
| 2-aminobutyric acid | 35 | C02721 | 17% | 43% |
| 1,2,4-benzenetriol | 26704 | C02814 | 0% | 57% |
| 390218 | 390218 |  | 100% | 97% |
| 390198 | 390198 |  | 100% | 93% |
| 356925 | 356925 |  | 100% | 47% |
| 210714 | 210714 |  | 100% | 100% |
| 170278 | 170278 |  | 100% | 0% |
| 161085 | 161085 |  | 100% | 90% |
| 110022 | 110022 |  | 100% | 3% |
| 100897 | 100897 |  | 100% | 0% |
| 86075 | 86075 |  | 100% | 3% |
| 31563 | 31563 |  | 100% | 3% |
| 31547 | 31547 |  | 100% | 97% |
| 18157 | 18157 |  | 100% | 0% |
| 11353 | 11353 |  | 100% | 93% |
| 8240 | 8240 |  | 100% | 20% |
| 4746 | 4746 |  | 100% | 100% |
| 4723 | 4723 |  | 100% | 67% |
| 4721 | 4721 |  | 100% | 77% |
| 4550 | 4550 |  | 100% | 100% |
| 3029 | 3029 |  | 100% | 100% |
| 2551 | 2551 |  | 100% | 93% |
| 2193 | 2193 |  | 100% | 0% |
| 1981 | 1981 |  | 100% | 100% |
| 1715 | 1715 |  | 100% | 17% |
| 168 | 168 |  | 100% | 100% |
| 345452 | 345452 |  | 97% | 83% |
| 228052 | 228052 |  | 97% | 77% |
| 210231 | 210231 |  | 97% | 47% |
| 170069 | 170069 |  | 97% | 87% |
| 100326 | 100326 |  | 97% | 0% |
| 18197 | 18197 |  | 97% | 0% |
| 1690 | 1690 |  | 97% | 0% |
| 424855 | 424855 |  | 93% | 0% |
| 395982 | 395982 |  | 93% | 0% |
| 376650 | 376650 |  | 93% | 57% |
| 345404 | 345404 |  | 93% | 37% |
| 209677 | 209677 |  | 93% | 3% |
| 1862 | 1862 |  | 93% | 97% |
| 379374 | 379374 |  | 90% | 23% |
| 230854 | 230854 |  | 90% | 87% |
| 100343 | 100343 |  | 90% | 0% |
| 17651 | 17651 |  | 90% | 100% |
| 17297 | 17297 |  | 90% | 0% |
| 4541 | 4541 |  | 90% | 23% |
| 3173 | 3173 |  | 90% | 80% |
| 1912 | 1912 |  | 90% | 100% |
| 119023 | 119023 |  | 87% | 100% |
| 110018 | 110018 |  | 87% | 0% |
| 87822 | 87822 |  | 87% | 20% |
| 41989 | 41989 |  | 87% | 0% |
| 34163 | 34163 |  | 87% | 3% |
| 6646 | 6646 |  | 87% | 97% |
| 1684 | 1684 |  | 87% | 27% |
| 382585 | 382585 |  | 83% | 93% |
| 346241 | 346241 |  | 83% | 90% |
| 247758 | 247758 |  | 83% | 60% |
| 131558 | 131558 |  | 83% | 0% |
| 84213 | 84213 |  | 83% | 0% |
| 17664 | 17664 |  | 83% | 7% |
| 12444 | 12444 |  | 83% | 50% |
| 1996 | 1996 |  | 83% | 93% |
| 134 | 134 |  | 83% | 100% |
| 418093 | 418093 |  | 80% | 43% |
| 346561 | 346561 |  | 80% | 47% |
| 217920 | 217920 |  | 80% | 0% |
| 209167 | 209167 |  | 80% | 97% |
| 7543 | 7543 |  | 80% | 0% |
| 5837 | 5837 |  | 80% | 83% |
| 84185 | 84185 |  | 77% | 20% |
| 46377 | 46377 |  | 77% | 100% |
| 4877 | 4877 |  | 77% | 100% |
| 1673 | 1673 |  | 77% | 17% |
| 367353 | 367353 |  | 73% | 0% |
| 110343 | 110343 |  | 73% | 7% |
| 34113 | 34113 |  | 73% | 70% |
| 61 | 61 |  | 73% | 100% |
| 395912 | 395912 |  | 70% | 53% |
| 360154 | 360154 |  | 70% | 97% |
| 187846 | 187846 |  | 70% | 0% |
| 100017 | 100017 |  | 70% | 0% |
| 34110 | 34110 |  | 70% | 7% |
| 31764 | 31764 |  | 70% | 7% |
| 91 | 91 |  | 70% | 87% |
| 407455 | 407455 |  | 67% | 63% |
| 380761 | 380761 |  | 67% | 3% |
| 370223 | 370223 |  | 67% | 27% |
| 110321 | 110321 |  | 67% | 100% |
| 2821 | 2821 |  | 67% | 0% |
| 414134 | 414134 |  | 63% | 100% |
| 382090 | 382090 |  | 63% | 67% |
| 210343 | 210343 |  | 63% | 100% |
| 100248 | 100248 |  | 63% | 0% |
| 100036 | 100036 |  | 63% | 0% |
| 97456 | 97456 |  | 63% | 0% |
| 388180 | 388180 |  | 60% | 83% |
| 241684 | 241684 |  | 60% | 93% |
| 223203 | 223203 |  | 60% | 0% |
| 5244 | 5244 |  | 60% | 30% |
| 391855 | 391855 |  | 57% | 13% |
| 390122 | 390122 |  | 57% | 57% |
| 382579 | 382579 |  | 57% | 57% |
| 223106 | 223106 |  | 57% | 0% |
| 33282 | 33282 |  | 57% | 3% |
| 351222 | 351222 |  | 53% | 87% |
| 109386 | 109386 |  | 53% | 70% |
| 7458 | 7458 |  | 53% | 100% |
| 341970 | 341970 |  | 50% | 30% |
| 228119 | 228119 |  | 50% | 63% |
| 210312 | 210312 |  | 50% | 0% |
| 4543 | 4543 |  | 50% | 3% |
| 416738 | 416738 |  | 47% | 0% |
| 405512 | 405512 |  | 47% | 27% |
| 382599 | 382599 |  | 47% | 0% |
| 346884 | 346884 |  | 47% | 87% |
| 217691 | 217691 |  | 47% | 27% |
| 41866 | 41866 |  | 47% | 23% |
| 34089 | 34089 |  | 47% | 7% |
| 419123 | 419123 |  | 43% | 0% |
| 329772 | 329772 |  | 43% | 0% |
| 210272 | 210272 |  | 43% | 43% |
| 128767 | 128767 |  | 43% | 0% |
| 127277 | 127277 |  | 43% | 0% |
| 41833 | 41833 |  | 43% | 67% |
| 31695 | 31695 |  | 43% | 53% |
| 3232 | 3232 |  | 43% | 0% |
| 371557 | 371557 |  | 40% | 7% |
| 342924 | 342924 |  | 40% | 77% |
| 210239 | 210239 |  | 40% | 80% |
| 209679 | 209679 |  | 40% | 0% |
| 136398 | 136398 |  | 40% | 0% |
| 34007 | 34007 |  | 40% | 43% |
| 390185 | 390185 |  | 37% | 20% |
| 210249 | 210249 |  | 37% | 0% |
| 114256 | 114256 |  | 37% | 67% |
| 100107 | 100107 |  | 37% | 0% |
| 41682 | 41682 |  | 37% | 3% |
| 412430 | 412430 |  | 33% | 37% |
| 404192 | 404192 |  | 33% | 77% |
| 390188 | 390188 |  | 33% | 77% |
| 390144 | 390144 |  | 33% | 30% |
| 377304 | 377304 |  | 33% | 0% |
| 130716 | 130716 |  | 33% | 0% |
| 119066 | 119066 |  | 33% | 100% |
| 32158 | 32158 |  | 33% | 3% |
| 18176 | 18176 |  | 33% | 3% |
| 16872 | 16872 |  | 33% | 0% |
| 3294 | 3294 |  | 33% | 0% |
| 68 | 68 |  | 33% | 43% |
| 382905 | 382905 |  | 30% | 0% |
| 353342 | 353342 |  | 30% | 70% |
| 344959 | 344959 |  | 30% | 97% |
| 223080 | 223080 |  | 30% | 97% |
| 210653 | 210653 |  | 30% | 67% |
| 119025 | 119025 |  | 30% | 0% |
| 41683 | 41683 |  | 30% | 67% |
| 26755 | 26755 |  | 30% | 0% |
| 21725 | 21725 |  | 30% | 0% |
| 17947 | 17947 |  | 30% | 43% |
| 782 | 782 |  | 30% | 7% |
| 413693 | 413693 |  | 27% | 0% |
| 390135 | 390135 |  | 27% | 100% |
| 384978 | 384978 |  | 27% | 0% |
| 378013 | 378013 |  | 27% | 0% |
| 377697 | 377697 |  | 27% | 3% |
| 377103 | 377103 |  | 27% | 10% |
| 330287 | 330287 |  | 27% | 0% |
| 161365 | 161365 |  | 27% | 43% |
| 131421 | 131421 |  | 27% | 0% |
| 119054 | 119054 |  | 27% | 3% |
| 41996 | 41996 |  | 27% | 17% |
| 34014 | 34014 |  | 27% | 7% |
| 14736 | 14736 |  | 27% | 10% |
| 4716 | 4716 |  | 27% | 3% |
| 3204 | 3204 |  | 27% | 10% |
| 408450 | 408450 |  | 23% | 10% |
| 391853 | 391853 |  | 23% | 47% |
| 371262 | 371262 |  | 23% | 20% |
| 342736 | 342736 |  | 23% | 0% |
| 211988 | 211988 |  | 23% | 97% |
| 210697 | 210697 |  | 23% | 0% |
| 190066 | 190066 |  | 23% | 0% |
| 171564 | 171564 |  | 23% | 13% |
| 135253 | 135253 |  | 23% | 0% |
| 125830 | 125830 |  | 23% | 0% |
| 109464 | 109464 |  | 23% | 7% |
| 20282 | 20282 |  | 23% | 67% |
| 18407 | 18407 |  | 23% | 0% |
| 16747 | 16747 |  | 23% | 30% |
| 376949 | 376949 |  | 20% | 23% |
| 357409 | 357409 |  | 20% | 0% |
| 356941 | 356941 |  | 20% | 17% |
| 354282 | 354282 |  | 20% | 13% |
| 189936 | 189936 |  | 20% | 0% |
| 135615 | 135615 |  | 20% | 20% |
| 131144 | 131144 |  | 20% | 0% |
| 110258 | 110258 |  | 20% | 0% |
| 105141 | 105141 |  | 20% | 0% |
| 100321 | 100321 |  | 20% | 0% |
| 22969 | 22969 |  | 20% | 0% |
| 14721 | 14721 |  | 20% | 0% |
| 7876 | 7876 |  | 20% | 17% |
| 4930 | 4930 |  | 20% | 3% |
| 390140 | 390140 |  | 17% | 7% |
| 135260 | 135260 |  | 17% | 37% |
| 133605 | 133605 |  | 17% | 17% |
| 133590 | 133590 |  | 17% | 3% |
| 33142 | 33142 |  | 17% | 30% |
| 18488 | 18488 |  | 17% | 90% |
| 377384 | 377384 |  | 13% | 17% |
| 371571 | 371571 |  | 13% | 7% |
| 209171 | 209171 |  | 13% | 7% |
| 126391 | 126391 |  | 13% | 87% |
| 121468 | 121468 |  | 13% | 73% |
| 121467 | 121467 |  | 13% | 20% |
| 100841 | 100841 |  | 13% | 37% |
| 46143 | 46143 |  | 13% | 7% |
| 41924 | 41924 |  | 13% | 57% |
| 34183 | 34183 |  | 13% | 40% |
| 27145 | 27145 |  | 13% | 73% |
| 17267 | 17267 |  | 13% | 57% |
| 414015 | 414015 |  | 10% | 13% |
| 390192 | 390192 |  | 10% | 17% |
| 359963 | 359963 |  | 10% | 20% |
| 321077 | 321077 |  | 10% | 33% |
| 223087 | 223087 |  | 10% | 20% |
| 210313 | 210313 |  | 10% | 40% |
| 100556 | 100556 |  | 10% | 67% |
| 18173 | 18173 |  | 10% | 13% |
| 2044 | 2044 |  | 10% | 10% |
| 1799 | 1799 |  | 10% | 10% |
| 892 | 892 |  | 10% | 23% |
| 1771 | 1771 |  | 0% | 100% |
| 88992 | 88992 |  | 0% | 100% |
| 97698 | 97698 |  | 0% | 100% |
| 413543 | 413543 |  | 0% | 100% |
| 4929 | 4929 |  | 0% | 100% |
| 97455 | 97455 |  | 0% | 100% |
| 4577 | 4577 |  | 0% | 100% |
| 98 | 98 |  | 0% | 100% |
| 390137 | 390137 |  | 0% | 100% |
| 108825 | 108825 |  | 0% | 100% |
| 88994 | 88994 |  | 0% | 100% |
| 161875 | 161875 |  | 0% | 100% |
| 97870 | 97870 |  | 0% | 100% |
| 347190 | 347190 |  | 0% | 100% |
| 4600 | 4600 |  | 0% | 100% |
| 88981 | 88981 |  | 0% | 100% |
| 401124 | 401124 |  | 0% | 100% |
| 367427 | 367427 |  | 0% | 100% |
| 110881 | 110881 |  | 0% | 100% |
| 109990 | 109990 |  | 0% | 100% |
| 97687 | 97687 |  | 0% | 97% |
| 110873 | 110873 |  | 0% | 97% |
| 391858 | 391858 |  | 0% | 97% |
| 5691 | 5691 |  | 0% | 97% |
| 110889 | 110889 |  | 0% | 97% |
| 89050 | 89050 |  | 0% | 97% |
| 44509 | 44509 |  | 0% | 97% |
| 11831 | 11831 |  | 0% | 97% |
| 4605 | 4605 |  | 0% | 97% |
| 4879 | 4879 |  | 0% | 97% |
| 390131 | 390131 |  | 0% | 97% |
| 31223 | 31223 |  | 0% | 97% |
| 110328 | 110328 |  | 0% | 97% |
| 111399 | 111399 |  | 0% | 93% |
| 119129 | 119129 |  | 0% | 93% |
| 4219 | 4219 |  | 3% | 93% |
| 210803 | 210803 |  | 0% | 93% |
| 355607 | 355607 |  | 0% | 93% |
| 357374 | 357374 |  | 0% | 93% |
| 357377 | 357377 |  | 0% | 93% |
| 109435 | 109435 |  | 0% | 90% |
| 31357 | 31357 |  | 0% | 90% |
| 48608 | 48608 |  | 0% | 90% |
| 573 | 573 |  | 0% | 90% |
| 237707 | 237707 |  | 0% | 90% |
| 5259 | 5259 |  | 0% | 90% |
| 21502 | 21502 |  | 0% | 90% |
| 2039 | 2039 |  | 0% | 87% |
| 43100 | 43100 |  | 3% | 87% |
| 210790 | 210790 |  | 0% | 87% |
| 88993 | 88993 |  | 0% | 87% |
| 4533 | 4533 |  | 0% | 87% |
| 390179 | 390179 |  | 0% | 87% |
| 4546 | 4546 |  | 0% | 87% |
| 610256 | 15370 |  | 0% | 83% |
| 98022 | 98022 |  | 0% | 83% |
| 234947 | 234947 |  | 0% | 83% |
| 112544 | 112544 |  | 0% | 83% |
| 307 | 307 |  | 0% | 80% |
| 215853 | 215853 |  | 0% | 80% |
| 364462 | 364462 |  | 0% | 80% |
| 120665 | 120665 |  | 0% | 80% |
| 107960 | 107960 |  | 0% | 77% |
| 458 | 458 |  | 0% | 77% |
| 342919 | 342919 |  | 0% | 77% |
| 89045 | 89045 |  | 0% | 73% |
| 110355 | 110355 |  | 0% | 73% |
| 5884 | 5884 |  | 0% | 73% |
| 8270 | 8270 |  | 0% | 73% |
| 356347 | 356347 |  | 0% | 73% |
| 386801 | 386801 |  | 0% | 70% |
| 6324 | 6324 |  | 0% | 70% |
| 421453 | 421453 |  | 0% | 70% |
| 112501 | 112501 |  | 0% | 70% |
| 18386 | 18386 |  | 0% | 67% |
| 320847 | 320847 |  | 0% | 67% |
| 351271 | 351271 |  | 0% | 67% |
| 112264 | 112264 |  | 0% | 67% |
| 214112 | 214112 |  | 0% | 67% |
| 104394 | 104394 |  | 0% | 67% |
| 20903 | 20903 |  | 0% | 63% |
| 390194 | 390194 |  | 0% | 63% |
| 188569 | 188569 |  | 0% | 60% |
| 20902 | 20902 |  | 0% | 57% |
| 390156 | 390156 |  | 3% | 57% |
| 135613 | 135613 |  | 0% | 57% |
| 5990 | 5990 |  | 7% | 57% |
| 391864 | 391864 |  | 0% | 57% |
| 376648 | 376648 |  | 3% | 53% |
| 54 | 54 |  | 3% | 53% |
| 397709 | 397709 |  | 0% | 53% |
| 110021 | 110021 |  | 0% | 53% |
| 413572 | 413572 |  | 0% | 53% |
| 171020 | 171020 |  | 0% | 53% |
| 1380 | 1380 |  | 0% | 50% |
| 7490 | 7490 |  | 0% | 50% |
| 391740 | 391740 |  | 0% | 50% |
| 47235 | 47235 |  | 0% | 50% |
| 224096 | 224096 |  | 0% | 50% |
| 401723 | 401723 |  | 0% | 47% |
| 21683 | 21683 |  | 0% | 47% |
| 133434 | 133434 |  | 0% | 47% |
| 231137 | 231137 |  | 0% | 47% |
| 250586 | 250586 |  | 0% | 47% |
| 100876 | 100876 |  | 0% | 43% |
| 17225 | 17225 |  | 0% | 43% |
| 356926 | 356926 |  | 0% | 43% |
| 112530 | 112530 |  | 0% | 43% |
| 133780 | 133780 |  | 0% | 43% |
| 365909 | 365909 |  | 0% | 43% |
| 360226 | 360226 |  | 3% | 43% |
| 22735 | 22735 |  | 0% | 43% |
| 104131 | 104131 |  | 0% | 40% |
| 46362 | 46362 |  | 0% | 40% |
| 228818 | 228818 |  | 7% | 40% |
| 379063 | 379063 |  | 7% | 40% |
| 370189 | 370189 |  | 0% | 40% |
| 4983 | 4983 |  | 0% | 40% |
| 365431 | 365431 |  | 0% | 40% |
| 249248 | 249248 |  | 0% | 40% |
| 231695 | 231695 |  | 0% | 40% |
| 26713 | 26713 |  | 3% | 37% |
| 161876 | 161876 |  | 0% | 37% |
| 4534 | 4534 |  | 0% | 37% |
| 6376 | 6376 |  | 3% | 37% |
| 592 | 592 |  | 0% | 37% |
| 168861 | 168861 |  | 0% | 37% |
| 95440 | 95440 |  | 0% | 37% |
| 110805 | 110805 |  | 0% | 37% |
| 31559 | 31559 |  | 0% | 37% |
| 97834 | 97834 |  | 0% | 37% |
| 347424 | 347424 |  | 0% | 37% |
| 327545 | 327545 |  | 0% | 37% |
| 120562 | 120562 |  | 0% | 33% |
| 85113 | 85113 |  | 0% | 33% |
| 389434 | 389434 |  | 0% | 33% |
| 365939 | 365939 |  | 3% | 33% |
| 341715 | 341715 |  | 0% | 33% |
| 380514 | 380514 |  | 7% | 30% |
| 171967 | 171967 |  | 0% | 30% |
| 391842 | 391842 |  | 0% | 30% |
| 34081 | 34081 |  | 0% | 30% |
| 46128 | 46128 |  | 0% | 30% |
| 127847 | 127847 |  | 0% | 30% |
| 34097 | 34097 |  | 0% | 30% |
| 46292 | 46292 |  | 0% | 30% |
| 11481 | 11481 |  | 0% | 30% |
| 391739 | 391739 |  | 0% | 30% |
| 110008 | 110008 |  | 0% | 30% |
| 31601 | 31601 |  | 0% | 30% |
| 26736 | 26736 |  | 0% | 30% |
| 408463 | 408463 |  | 0% | 27% |
| 390549 | 390549 |  | 3% | 27% |
| 349293 | 349293 |  | 0% | 27% |
| 46307 | 46307 |  | 0% | 27% |
| 188923 | 188923 |  | 3% | 27% |
| 2543 | 2543 |  | 0% | 27% |
| 85651 | 85651 |  | 0% | 27% |
| 4548 | 4548 |  | 0% | 27% |
| 384938 | 384938 |  | 3% | 23% |
| 395262 | 395262 |  | 0% | 23% |
| 376823 | 376823 |  | 0% | 23% |
| 356984 | 356984 |  | 3% | 23% |
| 384956 | 384956 |  | 3% | 23% |
| 34149 | 34149 |  | 0% | 23% |
| 133264 | 133264 |  | 0% | 23% |
| 329435 | 329435 |  | 0% | 23% |
| 370230 | 370230 |  | 0% | 23% |
| 120744 | 120744 |  | 0% | 23% |
| 390155 | 390155 |  | 3% | 23% |
| 26741 | 26741 |  | 0% | 23% |
| 253093 | 253093 |  | 0% | 23% |
| 231827 | 231827 |  | 0% | 23% |
| 372453 | 372453 |  | 0% | 20% |
| 379924 | 379924 |  | 0% | 20% |
| 52863 | 52863 |  | 7% | 20% |
| 390143 | 390143 |  | 0% | 20% |
| 168874 | 168874 |  | 0% | 20% |
| 110612 | 110612 |  | 0% | 20% |
| 384894 | 384894 |  | 0% | 20% |
| 106623 | 106623 |  | 0% | 20% |
| 33006 | 33006 |  | 0% | 20% |
| 17958 | 17958 |  | 0% | 20% |
| 118869 | 118869 |  | 0% | 20% |
| 9489 | 9489 |  | 0% | 20% |
| 377751 | 377751 |  | 0% | 20% |
| 213180 | 213180 |  | 0% | 20% |
| 108834 | 108834 |  | 0% | 20% |
| 391859 | 391859 |  | 0% | 20% |
| 64742 | 64742 |  | 0% | 20% |
| 130640 | 130640 |  | 0% | 20% |
| 31263 | 31263 |  | 7% | 17% |
| 352123 | 352123 |  | 3% | 17% |
| 380462 | 380462 |  | 3% | 17% |
| 160 | 160 |  | 3% | 17% |
| 53486 | 53486 |  | 7% | 17% |
| 224095 | 224095 |  | 7% | 17% |
| 4529 | 4529 |  | 7% | 13% |
| 384906 | 384906 |  | 7% | 13% |
| 94582 | 94582 |  | 7% | 13% |

**Table S6:** Reactome pathways found from serum proteins detected at the 10% FDR level. Entities found are the entered proteins, total entities are the reported proteins in the pathway. Pathways were cut off at p = 0.05.

| **Pathway Name** | **#Entities found** | **#Entities total** | **Entities p-value** | **Entities FDR** |
| --- | --- | --- | --- | --- |
| Post-translational protein phosphorylation | 60 | 107 | 1.11E-16 | 9.66E-15 |
| Regulation of Insulin-like Growth Factor (IGF) transport and uptake by Insulin-like Growth Factor Binding Proteins (IGFBPs) | 67 | 124 | 1.11E-16 | 9.66E-15 |
| Complement cascade | 72 | 146 | 1.11E-16 | 9.66E-15 |
| Regulation of Complement cascade | 64 | 135 | 1.11E-16 | 9.66E-15 |
| Binding and Uptake of Ligands by Scavenger Receptors | 56 | 129 | 1.11E-16 | 9.66E-15 |
| Initial triggering of complement | 47 | 111 | 1.11E-16 | 9.66E-15 |
| Creation of C4 and C2 activators | 40 | 103 | 1.11E-16 | 9.66E-15 |
| Neutrophil degranulation | 100 | 480 | 1.11E-16 | 9.66E-15 |
| Formation of Fibrin Clot (Clotting Cascade) | 28 | 39 | 1.11E-16 | 9.66E-15 |
| Platelet degranulation | 59 | 128 | 1.11E-16 | 9.66E-15 |
| Extracellular matrix organization | 76 | 301 | 1.11E-16 | 9.66E-15 |
| Response to elevated platelet cytosolic Ca2+ | 59 | 133 | 1.11E-16 | 9.66E-15 |
| Hemostasis | 121 | 726 | 1.11E-16 | 9.66E-15 |
| Innate Immune System | 187 | 1187 | 1.11E-16 | 9.66E-15 |
| Platelet activation, signaling and aggregation | 64 | 265 | 2.22E-16 | 1.80E-14 |
| Scavenging of heme from plasma | 38 | 99 | 3.33E-16 | 2.53E-14 |
| Intrinsic Pathway of Fibrin Clot Formation | 19 | 23 | 2.19E-14 | 1.55E-12 |
| Classical antibody-mediated complement activation | 34 | 95 | 7.65E-14 | 5.13E-12 |
| ECM proteoglycans | 30 | 76 | 2.00E-13 | 1.28E-11 |
| Common Pathway of Fibrin Clot Formation | 16 | 22 | 1.56E-11 | 9.54E-10 |
| CD22 mediated BCR regulation | 26 | 70 | 2.94E-11 | 1.71E-09 |
| Role of phospholipids in phagocytosis | 32 | 114 | 1.88E-10 | 9.97E-09 |
| FCGR activation | 30 | 101 | 1.88E-10 | 9.97E-09 |
| Scavenging by Class A Receptors | 14 | 19 | 2.44E-10 | 1.22E-08 |
| Cell surface interactions at the vascular wall | 48 | 246 | 1.29E-09 | 6.21E-08 |
| Integrin cell surface interactions | 26 | 85 | 1.67E-09 | 7.69E-08 |
| Degradation of the extracellular matrix | 33 | 140 | 6.87E-09 | 3.09E-07 |
| Antigen activates B Cell Receptor (BCR) leading to generation of second messengers | 26 | 95 | 1.54E-08 | 6.62E-07 |
| Diseases associated with glycosaminoglycan metabolism | 17 | 41 | 1.59E-08 | 6.69E-07 |
| Immune System | 240 | 2398 | 2.38E-08 | 9.53E-07 |
| Adherens junctions interactions | 15 | 33 | 3.41E-08 | 1.33E-06 |
| FCGR3A-mediated IL10 synthesis | 30 | 128 | 3.74E-08 | 1.42E-06 |
| Keratan sulfate degradation | 10 | 13 | 5.96E-08 | 2.21E-06 |
| Regulation of actin dynamics for phagocytic cup formation | 32 | 150 | 1.08E-07 | 3.79E-06 |
| Plasma lipoprotein assembly, remodeling, and clearance | 21 | 72 | 1.31E-07 | 4.46E-06 |
| HDL remodeling | 9 | 11 | 1.64E-07 | 5.40E-06 |
| Terminal pathway of complement | 8 | 8 | 1.79E-07 | 5.89E-06 |
| Plasma lipoprotein remodeling | 14 | 33 | 2.21E-07 | 6.85E-06 |
| Glycosaminoglycan metabolism | 28 | 124 | 2.21E-07 | 6.85E-06 |
| Fcgamma receptor (FCGR) dependent phagocytosis | 34 | 175 | 3.67E-07 | 1.10E-05 |
| Defects of contact activation system (CAS) and kallikrein/kinin system (KKS) | 10 | 16 | 3.92E-07 | 1.14E-05 |
| Diseases of hemostasis | 10 | 16 | 3.92E-07 | 1.14E-05 |
| Transport of gamma-carboxylated protein precursors from the endoplasmic reticulum to the Golgi apparatus | 8 | 9 | 4.30E-07 | 1.20E-05 |
| Diseases of glycosylation | 30 | 146 | 5.77E-07 | 1.56E-05 |
| Role of LAT2/NTAL/LAB on calcium mobilization | 24 | 102 | 7.88E-07 | 2.13E-05 |
| FCGR3A-mediated phagocytosis | 30 | 149 | 8.69E-07 | 2.17E-05 |
| Parasite infection | 30 | 149 | 8.69E-07 | 2.17E-05 |
| Leishmania phagocytosis | 30 | 149 | 8.69E-07 | 2.17E-05 |
| Removal of aminoterminal propeptides from gamma-carboxylated proteins | 8 | 10 | 9.38E-07 | 2.25E-05 |
| Gamma-carboxylation of protein precursors | 8 | 10 | 9.38E-07 | 2.25E-05 |
| Activation of C3 and C5 | 7 | 7 | 1.05E-06 | 2.42E-05 |
| Plasma lipoprotein assembly | 10 | 19 | 1.80E-06 | 4.15E-05 |
| Keratan sulfate/keratin metabolism | 13 | 34 | 1.84E-06 | 4.15E-05 |
| Gamma-carboxylation, transport, and amino-terminal cleavage of proteins | 8 | 11 | 1.89E-06 | 4.15E-05 |
| Immunoregulatory interactions between a Lymphoid and a non-Lymphoid cell | 46 | 297 | 1.90E-06 | 4.19E-05 |
| Non-integrin membrane-ECM interactions | 17 | 59 | 2.35E-06 | 4.94E-05 |
| Metabolism of carbohydrates | 46 | 300 | 2.47E-06 | 5.19E-05 |
| Defective B4GALT1 causes B4GALT1-CDG (CDG-2d) | 7 | 8 | 2.51E-06 | 5.28E-05 |
| Formation of the cornified envelope | 26 | 129 | 4.49E-06 | 8.99E-05 |
| Retinoid metabolism and transport | 14 | 44 | 6.02E-06 | 1.20E-04 |
| FCERI mediated Ca+2 mobilization | 24 | 117 | 7.78E-06 | 1.48E-04 |
| Diseases of metabolism | 39 | 249 | 8.91E-06 | 1.69E-04 |
| FCERI mediated MAPK activation | 24 | 119 | 1.02E-05 | 1.91E-04 |
| Chylomicron assembly | 7 | 10 | 1.06E-05 | 1.91E-04 |
| Chylomicron remodeling | 7 | 10 | 1.06E-05 | 1.91E-04 |
| CS/DS degradation | 8 | 14 | 1.08E-05 | 1.94E-04 |
| Metabolism of fat-soluble vitamins | 14 | 48 | 1.57E-05 | 2.66E-04 |
| Molecules associated with elastic fibres | 12 | 38 | 2.93E-05 | 4.96E-04 |
| Lectin pathway of complement activation | 6 | 8 | 3.10E-05 | 4.96E-04 |
| Defective CHST6 causes MCDC1 | 6 | 8 | 3.10E-05 | 4.96E-04 |
| Defective ST3GAL3 causes MCT12 and EIEE15 | 6 | 8 | 3.10E-05 | 4.96E-04 |
| Gluconeogenesis | 11 | 34 | 5.03E-05 | 8.05E-04 |
| Cell-cell junction organization | 15 | 65 | 1.09E-04 | 1.74E-03 |
| Signaling by the B Cell Receptor (BCR) | 28 | 176 | 1.23E-04 | 1.92E-03 |
| Defective B3GALT6 causes EDSP2 and SEMDJL1 | 8 | 20 | 1.28E-04 | 1.92E-03 |
| Defective B4GALT7 causes EDS, progeroid type | 8 | 20 | 1.28E-04 | 1.92E-03 |
| Defective B3GAT3 causes JDSSDHD | 8 | 20 | 1.28E-04 | 1.92E-03 |
| Elastic fibre formation | 12 | 45 | 1.42E-04 | 2.13E-03 |
| Detoxification of Reactive Oxygen Species | 11 | 39 | 1.65E-04 | 2.48E-03 |
| Cell junction organization | 18 | 92 | 1.81E-04 | 2.72E-03 |
| LDL remodeling | 4 | 4 | 2.29E-04 | 3.20E-03 |
| Keratan sulfate biosynthesis | 9 | 28 | 2.50E-04 | 3.49E-03 |
| Collagen degradation | 14 | 64 | 3.10E-04 | 4.27E-03 |
| Amyloid fiber formation | 16 | 80 | 3.19E-04 | 4.27E-03 |
| Defective CHSY1 causes TPBS | 5 | 8 | 3.29E-04 | 4.27E-03 |
| Defective CHST14 causes EDS, musculocontractural type | 5 | 8 | 3.29E-04 | 4.27E-03 |
| Defective CHST3 causes SEDCJD | 5 | 8 | 3.29E-04 | 4.27E-03 |
| Erythrocytes take up oxygen and release carbon dioxide | 5 | 8 | 3.29E-04 | 4.27E-03 |
| Chondroitin sulfate/dermatan sulfate metabolism | 12 | 50 | 3.64E-04 | 4.73E-03 |
| Vesicle-mediated transport | 81 | 761 | 3.72E-04 | 4.84E-03 |
| Laminin interactions | 9 | 30 | 4.09E-04 | 5.32E-03 |
| Dissolution of Fibrin Clot | 6 | 13 | 4.22E-04 | 5.48E-03 |
| Alternative complement activation | 4 | 5 | 5.28E-04 | 6.33E-03 |
| Ficolins bind to repetitive carbohydrate structures on the target cell surface | 4 | 5 | 5.28E-04 | 6.33E-03 |
| Regulation of TLR by endogenous ligand | 7 | 19 | 5.43E-04 | 6.52E-03 |
| Defective factor IX causes hemophilia B | 5 | 9 | 5.59E-04 | 6.70E-03 |
| Assembly of collagen fibrils and other multimeric structures | 13 | 61 | 6.35E-04 | 7.62E-03 |
| FCERI mediated NF-kB activation | 25 | 167 | 6.60E-04 | 7.92E-03 |
| A tetrasaccharide linker sequence is required for GAG synthesis | 8 | 26 | 7.19E-04 | 8.63E-03 |
| Plasma lipoprotein clearance | 9 | 33 | 7.99E-04 | 8.79E-03 |
| Activation of Matrix Metalloproteinases | 9 | 33 | 7.99E-04 | 8.79E-03 |
| Platelet Aggregation (Plug Formation) | 10 | 40 | 8.08E-04 | 8.89E-03 |
| Heparan sulfate/heparin (HS-GAG) metabolism | 12 | 55 | 8.29E-04 | 9.12E-03 |
| Scavenging by Class B Receptors | 4 | 6 | 1.03E-03 | 1.14E-02 |
| Defective F9 activation | 4 | 6 | 1.03E-03 | 1.14E-02 |
| Collagen formation | 16 | 90 | 1.09E-03 | 1.20E-02 |
| Chaperone Mediated Autophagy | 7 | 22 | 1.26E-03 | 1.39E-02 |
| Dermatan sulfate biosynthesis | 5 | 11 | 1.35E-03 | 1.44E-02 |
| Apoptotic cleavage of cell adhesion proteins | 5 | 11 | 1.35E-03 | 1.44E-02 |
| Defective SERPING1 causes hereditary angioedema | 3 | 3 | 1.44E-03 | 1.44E-02 |
| Defective factor XII causes hereditary angioedema | 3 | 3 | 1.44E-03 | 1.44E-02 |
| Collagen biosynthesis and modifying enzymes | 13 | 67 | 1.46E-03 | 1.46E-02 |
| Cell-Cell communication | 20 | 130 | 1.60E-03 | 1.60E-02 |
| L1CAM interactions | 19 | 121 | 1.65E-03 | 1.65E-02 |
| Post-translational modification: synthesis of GPI-anchored proteins | 16 | 94 | 1.69E-03 | 1.69E-02 |
| MET activates PTK2 signaling | 8 | 30 | 1.76E-03 | 1.76E-02 |
| Scavenging by Class F Receptors | 4 | 7 | 1.81E-03 | 1.78E-02 |
| Nectin/Necl trans heterodimerization | 4 | 7 | 1.81E-03 | 1.78E-02 |
| Defective factor VIII causes hemophilia A | 4 | 7 | 1.81E-03 | 1.78E-02 |
| GP1b-IX-V activation signalling | 5 | 12 | 1.97E-03 | 1.78E-02 |
| Erythrocytes take up carbon dioxide and release oxygen | 5 | 12 | 1.97E-03 | 1.78E-02 |
| O2/CO2 exchange in erythrocytes | 5 | 12 | 1.97E-03 | 1.78E-02 |
| Metabolism of Angiotensinogen to Angiotensins | 6 | 18 | 2.20E-03 | 1.98E-02 |
| Chondroitin sulfate biosynthesis | 6 | 20 | 3.67E-03 | 3.31E-02 |
| Receptor-type tyrosine-protein phosphatases | 6 | 20 | 3.67E-03 | 3.31E-02 |
| NCAM1 interactions | 9 | 42 | 3.99E-03 | 3.59E-02 |
| Gamma carboxylation, hypusine formation and arylsulfatase activation | 9 | 42 | 3.99E-03 | 3.59E-02 |
| CHL1 interactions | 4 | 9 | 4.42E-03 | 3.98E-02 |
| EPH-ephrin mediated repulsion of cells | 10 | 51 | 4.58E-03 | 4.04E-02 |
| p130Cas linkage to MAPK signaling for integrins | 5 | 15 | 5.06E-03 | 4.04E-02 |
| GRB2:SOS provides linkage to MAPK signaling for Integrins | 5 | 15 | 5.06E-03 | 4.04E-02 |
| Platelet Adhesion to exposed collagen | 5 | 15 | 5.06E-03 | 4.04E-02 |
| Leishmania parasite growth and survival | 31 | 259 | 5.12E-03 | 4.09E-02 |
| Anti-inflammatory response favouring Leishmania parasite infection | 31 | 259 | 5.12E-03 | 4.09E-02 |
| Collagen chain trimerization | 9 | 44 | 5.35E-03 | 4.28E-02 |
| VLDL assembly | 3 | 5 | 5.98E-03 | 4.78E-02 |
| Extrinsic Pathway of Fibrin Clot Formation | 3 | 5 | 5.98E-03 | 4.78E-02 |
| Reactions specific to the complex N-glycan synthesis pathway | 4 | 10 | 6.37E-03 | 5.09E-02 |

**Table S7:** Reactome pathways found from CSF proteins detected at the 10% FDR level. Entities found are the entered proteins, total entities are the reported proteins in the pathway. Pathways were cut off at p = 0.05.

| **Pathway Name** | **#Entities found** | **#Entities total** | **Entities p-value** | **Entities FDR** |
| --- | --- | --- | --- | --- |
| Post-translational protein phosphorylation | 6.60E+01 | 107 | 1.11E-16 | 1.21E-14 |
| Regulation of Insulin-like Growth Factor (IGF) transport and uptake by Insulin-like Growth Factor Binding Proteins (IGFBPs) | 7.30E+01 | 124 | 1.11E-16 | 1.21E-14 |
| Complement cascade | 72 | 146 | 1.11E-16 | 1.21E-14 |
| Regulation of Complement cascade | 65 | 135 | 1.11E-16 | 1.21E-14 |
| Binding and Uptake of Ligands by Scavenger Receptors | 57 | 129 | 1.11E-16 | 1.21E-14 |
| Initial triggering of complement | 46 | 111 | 1.11E-16 | 1.21E-14 |
| Neutrophil degranulation | 109 | 480 | 1.11E-16 | 1.21E-14 |
| Formation of Fibrin Clot (Clotting Cascade) | 28 | 39 | 1.11E-16 | 1.21E-14 |
| Platelet degranulation | 60 | 128 | 1.11E-16 | 1.21E-14 |
| Extracellular matrix organization | 84 | 301 | 1.11E-16 | 1.21E-14 |
| Response to elevated platelet cytosolic Ca2+ | 60 | 133 | 1.11E-16 | 1.21E-14 |
| Innate Immune System | 197 | 1187 | 1.11E-16 | 1.21E-14 |
| Hemostasis | 128 | 726 | 2.33E-15 | 2.35E-13 |
| Creation of C4 and C2 activators | 39 | 103 | 1.90E-14 | 1.78E-12 |
| Scavenging of heme from plasma | 38 | 99 | 2.72E-14 | 2.37E-12 |
| Platelet activation, signaling and aggregation | 65 | 265 | 4.11E-14 | 3.37E-12 |
| Intrinsic Pathway of Fibrin Clot Formation | 19 | 23 | 2.73E-13 | 2.10E-11 |
| ECM proteoglycans | 31 | 76 | 1.41E-12 | 1.03E-10 |
| Classical antibody-mediated complement activation | 34 | 95 | 4.04E-12 | 2.79E-10 |
| Integrin cell surface interactions | 30 | 85 | 1.02E-10 | 6.61E-09 |
| Common Pathway of Fibrin Clot Formation | 16 | 22 | 1.27E-10 | 7.89E-09 |
| CD22 mediated BCR regulation | 26 | 70 | 6.23E-10 | 3.68E-08 |
| Scavenging by Class A Receptors | 14 | 19 | 1.53E-09 | 8.72E-08 |
| Degradation of the extracellular matrix | 37 | 140 | 1.95E-09 | 1.05E-07 |
| Cell surface interactions at the vascular wall | 52 | 246 | 2.45E-09 | 1.27E-07 |
| FCGR activation | 30 | 101 | 5.16E-09 | 2.58E-07 |
| Role of phospholipids in phagocytosis | 32 | 114 | 6.01E-09 | 2.88E-07 |
| Diseases associated with glycosaminoglycan metabolism | 18 | 41 | 2.28E-08 | 1.07E-06 |
| Glycosaminoglycan metabolism | 31 | 124 | 1.30E-07 | 5.86E-06 |
| Immune System | 267 | 2398 | 1.57E-07 | 6.74E-06 |
| Adherens junctions interactions | 15 | 33 | 2.14E-07 | 8.69E-06 |
| Metabolism of carbohydrates | 54 | 300 | 2.17E-07 | 8.69E-06 |
| Keratan sulfate degradation | 10 | 13 | 2.23E-07 | 8.69E-06 |
| Antigen activates B Cell Receptor (BCR) leading to generation of second messengers | 26 | 95 | 2.50E-07 | 9.51E-06 |
| Plasma lipoprotein assembly, remodeling, and clearance | 22 | 72 | 3.47E-07 | 1.28E-05 |
| Diseases of glycosylation | 33 | 146 | 4.83E-07 | 1.74E-05 |
| Terminal pathway of complement | 8 | 8 | 5.26E-07 | 1.84E-05 |
| HDL remodeling | 9 | 11 | 5.40E-07 | 1.84E-05 |
| FCGR3A-mediated IL10 synthesis | 30 | 128 | 7.68E-07 | 2.53E-05 |
| Plasma lipoprotein assembly | 11 | 19 | 8.94E-07 | 2.86E-05 |
| Plasma lipoprotein remodeling | 14 | 33 | 1.20E-06 | 3.84E-05 |
| Transport of gamma-carboxylated protein precursors from the endoplasmic reticulum to the Golgi apparatus | 8 | 9 | 1.25E-06 | 3.89E-05 |
| Diseases of hemostasis | 10 | 16 | 1.42E-06 | 4.12E-05 |
| Defects of contact activation system (CAS) and kallikrein/kinin system (KKS) | 10 | 16 | 1.42E-06 | 4.12E-05 |
| Gluconeogenesis | 14 | 34 | 1.69E-06 | 4.89E-05 |
| Regulation of actin dynamics for phagocytic cup formation | 32 | 150 | 2.36E-06 | 6.62E-05 |
| Activation of C3 and C5 | 7 | 7 | 2.70E-06 | 7.04E-05 |
| Chylomicron assembly | 8 | 10 | 2.71E-06 | 7.04E-05 |
| Removal of aminoterminal propeptides from gamma-carboxylated proteins | 8 | 10 | 2.71E-06 | 7.04E-05 |
| Gamma-carboxylation of protein precursors | 8 | 10 | 2.71E-06 | 7.04E-05 |
| CS/DS degradation | 9 | 14 | 3.79E-06 | 9.48E-05 |
| Non-integrin membrane-ECM interactions | 18 | 59 | 4.00E-06 | 1.00E-04 |
| Gamma-carboxylation, transport, and amino-terminal cleavage of proteins | 8 | 11 | 5.40E-06 | 1.30E-04 |
| Defective B4GALT1 causes B4GALT1-CDG (CDG-2d) | 7 | 8 | 6.41E-06 | 1.54E-04 |
| Formation of the cornified envelope | 28 | 129 | 7.26E-06 | 1.67E-04 |
| Fcgamma receptor (FCGR) dependent phagocytosis | 34 | 175 | 8.23E-06 | 1.89E-04 |
| Keratan sulfate/keratin metabolism | 13 | 34 | 8.56E-06 | 1.96E-04 |
| Role of LAT2/NTAL/LAB on calcium mobilization | 24 | 102 | 8.89E-06 | 1.96E-04 |
| Parasite infection | 30 | 149 | 1.43E-05 | 3.00E-04 |
| Leishmania phagocytosis | 30 | 149 | 1.43E-05 | 3.00E-04 |
| FCGR3A-mediated phagocytosis | 30 | 149 | 1.43E-05 | 3.00E-04 |
| Immunoregulatory interactions between a Lymphoid and a non-Lymphoid cell | 48 | 297 | 1.68E-05 | 3.53E-04 |
| Diseases of metabolism | 42 | 249 | 2.16E-05 | 4.32E-04 |
| Chylomicron remodeling | 7 | 10 | 2.65E-05 | 5.29E-04 |
| Retinoid metabolism and transport | 14 | 44 | 2.93E-05 | 5.56E-04 |
| Detoxification of Reactive Oxygen Species | 13 | 39 | 3.50E-05 | 6.64E-04 |
| Defective B3GALT6 causes EDSP2 and SEMDJL1 | 9 | 20 | 6.05E-05 | 1.09E-03 |
| Defective B4GALT7 causes EDS, progeroid type | 9 | 20 | 6.05E-05 | 1.09E-03 |
| Defective B3GAT3 causes JDSSDHD | 9 | 20 | 6.05E-05 | 1.09E-03 |
| Defective CHSY1 causes TPBS | 6 | 8 | 6.85E-05 | 1.16E-03 |
| Defective CHST3 causes SEDCJD | 6 | 8 | 6.85E-05 | 1.16E-03 |
| Defective CHST6 causes MCDC1 | 6 | 8 | 6.85E-05 | 1.16E-03 |
| Defective ST3GAL3 causes MCT12 and EIEE15 | 6 | 8 | 6.85E-05 | 1.16E-03 |
| Defective CHST14 causes EDS, musculocontractural type | 6 | 8 | 6.85E-05 | 1.16E-03 |
| Metabolism of fat-soluble vitamins | 14 | 48 | 7.31E-05 | 1.24E-03 |
| FCERI mediated Ca+2 mobilization | 24 | 117 | 7.52E-05 | 1.28E-03 |
| A tetrasaccharide linker sequence is required for GAG synthesis | 10 | 26 | 8.71E-05 | 1.39E-03 |
| FCERI mediated MAPK activation | 24 | 119 | 9.68E-05 | 1.55E-03 |
| Chondroitin sulfate/dermatan sulfate metabolism | 14 | 50 | 1.11E-04 | 1.78E-03 |
| Molecules associated with elastic fibres | 12 | 38 | 1.14E-04 | 1.82E-03 |
| Amyloid fiber formation | 18 | 80 | 1.93E-04 | 2.90E-03 |
| Collagen formation | 19 | 90 | 2.85E-04 | 4.28E-03 |
| Heparan sulfate/heparin (HS-GAG) metabolism | 14 | 55 | 2.90E-04 | 4.36E-03 |
| Receptor-type tyrosine-protein phosphatases | 8 | 20 | 3.36E-04 | 5.04E-03 |
| Cell junction organization | 19 | 92 | 3.72E-04 | 5.25E-03 |
| Dermatan sulfate biosynthesis | 6 | 11 | 3.75E-04 | 5.25E-03 |
| LDL remodeling | 4 | 4 | 3.93E-04 | 5.51E-03 |
| Collagen degradation | 15 | 64 | 4.22E-04 | 5.91E-03 |
| Cell-cell junction organization | 15 | 65 | 4.94E-04 | 6.91E-03 |
| Elastic fibre formation | 12 | 45 | 5.14E-04 | 7.19E-03 |
| GP1b-IX-V activation signalling | 6 | 12 | 5.90E-04 | 8.18E-03 |
| Chaperone Mediated Autophagy | 8 | 22 | 6.23E-04 | 8.18E-03 |
| Lectin pathway of complement activation | 5 | 8 | 6.29E-04 | 8.18E-03 |
| Erythrocytes take up oxygen and release carbon dioxide | 5 | 8 | 6.29E-04 | 8.18E-03 |
| Collagen biosynthesis and modifying enzymes | 15 | 67 | 6.69E-04 | 8.70E-03 |
| Keratan sulfate biosynthesis | 9 | 28 | 6.98E-04 | 9.07E-03 |
| Assembly of collagen fibrils and other multimeric structures | 14 | 61 | 7.89E-04 | 1.03E-02 |
| Dissolution of Fibrin Clot | 6 | 13 | 8.89E-04 | 1.08E-02 |
| Alternative complement activation | 4 | 5 | 9.00E-04 | 1.08E-02 |
| VLDL assembly | 4 | 5 | 9.00E-04 | 1.08E-02 |
| Ficolins bind to repetitive carbohydrate structures on the target cell surface | 4 | 5 | 9.00E-04 | 1.08E-02 |
| Defective factor IX causes hemophilia B | 5 | 9 | 1.06E-03 | 1.27E-02 |
| Laminin interactions | 9 | 30 | 1.12E-03 | 1.35E-02 |
| Signaling by the B Cell Receptor (BCR) | 28 | 176 | 1.15E-03 | 1.38E-02 |
| Post-translational modification: synthesis of GPI-anchored proteins | 18 | 94 | 1.21E-03 | 1.46E-02 |
| Regulation of TLR by endogenous ligand | 7 | 19 | 1.25E-03 | 1.50E-02 |
| Vesicle-mediated transport | 88 | 761 | 1.43E-03 | 1.71E-02 |
| L1CAM interactions | 21 | 121 | 1.66E-03 | 1.82E-02 |
| Chondroitin sulfate biosynthesis | 7 | 20 | 1.66E-03 | 1.83E-02 |
| Reactions specific to the complex N-glycan synthesis pathway | 5 | 10 | 1.68E-03 | 1.84E-02 |
| Scavenging by Class B Receptors | 4 | 6 | 1.75E-03 | 1.92E-02 |
| Defective F9 activation | 4 | 6 | 1.75E-03 | 1.92E-02 |
| Plasma lipoprotein clearance | 9 | 33 | 2.13E-03 | 2.34E-02 |
| Activation of Matrix Metalloproteinases | 9 | 33 | 2.13E-03 | 2.34E-02 |
| Defective SERPING1 causes hereditary angioedema | 3 | 3 | 2.16E-03 | 2.38E-02 |
| Defective factor XII causes hereditary angioedema | 3 | 3 | 2.16E-03 | 2.38E-02 |
| Platelet Aggregation (Plug Formation) | 10 | 40 | 2.32E-03 | 2.52E-02 |
| Apoptotic cleavage of cell adhesion proteins | 5 | 11 | 2.52E-03 | 2.52E-02 |
| Scavenging by Class F Receptors | 4 | 7 | 3.03E-03 | 3.03E-02 |
| Nectin/Necl trans heterodimerization | 4 | 7 | 3.03E-03 | 3.03E-02 |
| Defective factor VIII causes hemophilia A | 4 | 7 | 3.03E-03 | 3.03E-02 |
| NCAM1 interactions | 10 | 42 | 3.26E-03 | 3.26E-02 |
| Erythrocytes take up carbon dioxide and release oxygen | 5 | 12 | 3.65E-03 | 3.65E-02 |
| O2/CO2 exchange in erythrocytes | 5 | 12 | 3.65E-03 | 3.65E-02 |
| Cell-Cell communication | 21 | 130 | 3.77E-03 | 3.77E-02 |
| MET activates PTK2 signaling | 8 | 30 | 4.20E-03 | 4.04E-02 |
| Glucose metabolism | 17 | 98 | 4.38E-03 | 4.04E-02 |
| FCERI mediated NF-kB activation | 25 | 167 | 4.41E-03 | 4.04E-02 |
| Metabolism of Angiotensinogen to Angiotensins | 6 | 18 | 4.43E-03 | 4.04E-02 |
| Collagen chain trimerization | 10 | 44 | 4.49E-03 | 4.04E-02 |
| Scavenging by Class H Receptors | 3 | 4 | 4.81E-03 | 4.33E-02 |
| Trafficking and processing of endosomal TLR | 5 | 13 | 5.08E-03 | 4.58E-02 |
| Glycolysis | 14 | 78 | 6.94E-03 | 6.24E-02 |
| CHL1 interactions | 4 | 9 | 7.28E-03 | 6.55E-02 |
| Axonal growth inhibition (RHOA activation) | 4 | 9 | 7.28E-03 | 6.55E-02 |
| Cargo concentration in the ER | 8 | 33 | 7.28E-03 | 6.55E-02 |
| Syndecan interactions | 7 | 27 | 8.31E-03 | 7.28E-02 |
| Extrinsic Pathway of Fibrin Clot Formation | 3 | 5 | 8.84E-03 | 7.28E-02 |
| N-glycan trimming and elongation in the cis-Golgi | 3 | 5 | 8.84E-03 | 7.28E-02 |
| p130Cas linkage to MAPK signaling for integrins | 5 | 15 | 9.09E-03 | 7.28E-02 |
| GRB2:SOS provides linkage to MAPK signaling for Integrins | 5 | 15 | 9.09E-03 | 7.28E-02 |
| Platelet Adhesion to exposed collagen | 5 | 15 | 9.09E-03 | 7.28E-02 |
| Gamma carboxylation, hypusine formation and arylsulfatase activation | 9 | 42 | 9.77E-03 | 7.82E-02 |
| p75NTR regulates axonogenesis | 4 | 10 | 1.04E-02 | 8.32E-02 |
| Protein-protein interactions at synapses | 15 | 91 | 1.10E-02 | 8.79E-02 |
| EPH-ephrin mediated repulsion of cells | 10 | 51 | 1.18E-02 | 9.48E-02 |
| Signaling by FGFR1 amplification mutants | 2 | 2 | 1.24E-02 | 9.95E-02 |
| Signaling by FGFR2 amplification mutants | 2 | 2 | 1.24E-02 | 9.95E-02 |
| Transfer of LPS from LBP carrier to CD14 | 2 | 2 | 1.24E-02 | 9.95E-02 |
| Defective POMGNT1 causes MDDGA3, MDDGB3 and MDDGC3 | 2 | 2 | 1.24E-02 | 9.95E-02 |
| Gene and protein expression by JAK-STAT signaling after Interleukin-12 stimulation | 8 | 37 | 1.37E-02 | 1.10E-01 |
| Interleukin-4 and Interleukin-13 signaling | 17 | 111 | 1.38E-02 | 1.10E-01 |
| Downregulation of ERBB4 signaling | 4 | 11 | 1.43E-02 | 1.14E-01 |
| Mucopolysaccharidoses | 4 | 11 | 1.43E-02 | 1.14E-01 |
| VLDL clearance | 3 | 6 | 1.44E-02 | 1.15E-01 |
| Metal sequestration by antimicrobial proteins | 3 | 6 | 1.44E-02 | 1.15E-01 |
| Maturation of spike protein | 3 | 6 | 1.44E-02 | 1.15E-01 |
| Hyaluronan metabolism | 5 | 17 | 1.49E-02 | 1.17E-01 |
| NR1H3 & NR1H2 regulate gene expression linked to cholesterol transport and efflux | 8 | 38 | 1.58E-02 | 1.17E-01 |
| Glycosphingolipid metabolism | 9 | 46 | 1.67E-02 | 1.17E-01 |
| Interleukin-12 signaling | 9 | 46 | 1.67E-02 | 1.17E-01 |
| Keratinization | 28 | 217 | 1.74E-02 | 1.22E-01 |
| Signaling downstream of RAS mutants | 9 | 47 | 1.88E-02 | 1.32E-01 |
| Signaling by moderate kinase activity BRAF mutants | 9 | 47 | 1.88E-02 | 1.32E-01 |
| Paradoxical activation of RAF signaling by kinase inactive BRAF | 9 | 47 | 1.88E-02 | 1.32E-01 |
| Signaling by RAS mutants | 9 | 47 | 1.88E-02 | 1.32E-01 |
| Hyaluronan uptake and degradation | 4 | 12 | 1.90E-02 | 1.33E-01 |
| NCAM signaling for neurite out-growth | 11 | 64 | 2.06E-02 | 1.44E-01 |
| Interleukin-12 family signaling | 10 | 56 | 2.11E-02 | 1.48E-01 |
| Neurofascin interactions | 3 | 7 | 2.15E-02 | 1.50E-01 |
| NrCAM interactions | 3 | 7 | 2.15E-02 | 1.50E-01 |
| Signaling by FGFR in disease | 12 | 73 | 2.17E-02 | 1.52E-01 |
| LDL clearance | 5 | 19 | 2.27E-02 | 1.59E-01 |
| N-glycan antennae elongation in the medial/trans-Golgi | 6 | 26 | 2.33E-02 | 1.59E-01 |
| MET promotes cell motility | 8 | 41 | 2.35E-02 | 1.59E-01 |
| Diseases of carbohydrate metabolism | 7 | 34 | 2.57E-02 | 1.59E-01 |
| Progressive trimming of alpha-1,2-linked mannose residues from Man9/8/7GlcNAc2 to produce Man5GlcNAc2 | 2 | 3 | 2.65E-02 | 1.59E-01 |
| Manipulation of host energy metabolism | 2 | 3 | 2.65E-02 | 1.59E-01 |
| Defective F9 variant does not activate FX | 2 | 3 | 2.65E-02 | 1.59E-01 |
| Defective factor IX causes thrombophilia | 2 | 3 | 2.65E-02 | 1.59E-01 |
| Defective F8 cleavage by thrombin | 2 | 3 | 2.65E-02 | 1.59E-01 |
| Defective cofactor function of FVIIIa variant | 2 | 3 | 2.65E-02 | 1.59E-01 |
| Defective NEU1 causes sialidosis | 2 | 3 | 2.65E-02 | 1.59E-01 |
| Signaling by RAF1 mutants | 8 | 42 | 2.66E-02 | 1.59E-01 |
| Leishmania parasite growth and survival | 31 | 259 | 3.15E-02 | 1.89E-01 |
| Anti-inflammatory response favouring Leishmania parasite infection | 31 | 259 | 3.15E-02 | 1.89E-01 |
| Signaling by PDGF | 10 | 60 | 3.16E-02 | 1.89E-01 |
| Signaling by high-kinase activity BRAF mutants | 7 | 37 | 3.77E-02 | 2.26E-01 |
| HS-GAG degradation | 5 | 22 | 3.88E-02 | 2.33E-01 |
| Diseases associated with O-glycosylation of proteins | 11 | 71 | 3.89E-02 | 2.34E-01 |
| Signaling by MET | 12 | 80 | 3.93E-02 | 2.36E-01 |
| Apoptotic cleavage of cellular proteins | 7 | 38 | 4.25E-02 | 2.55E-01 |
| Visual phototransduction | 14 | 100 | 4.46E-02 | 2.67E-01 |
| Neurophilin interactions with VEGF and VEGFR | 2 | 4 | 4.46E-02 | 2.68E-01 |
| Signaling by FGFR2 IIIa TM | 5 | 23 | 4.54E-02 | 2.72E-01 |
| NR1H2 and NR1H3-mediated signaling | 8 | 48 | 5.09E-02 | 3.05E-01 |
| Regulation of commissural axon pathfinding by SLIT and ROBO | 3 | 10 | 5.23E-02 | 3.14E-01 |

**Table S8:** Reactome pathways found from serum metabolites detected at the 10% FDR level. Entities found are the entered proteins, total entities are the reported proteins in the pathway. Pathways were cut off at p = 0.05.

| **Pathway Name** | **#Entities found** | **#Entities total** | **Entities p-value** | **Entities FDR** |
| --- | --- | --- | --- | --- |
| tRNA Aminoacylation | 17 | 26 | 2.18E-11 | 6.42E-09 |
| Mitochondrial tRNA aminoacylation | 17 | 26 | 2.18E-11 | 6.42E-09 |
| Cytosolic tRNA aminoacylation | 17 | 26 | 2.18E-11 | 6.42E-09 |
| SLC-mediated transmembrane transport | 38 | 163 | 2.82E-10 | 6.20E-08 |
| SLC transporter disorders | 26 | 80 | 4.09E-10 | 7.21E-08 |
| Amino acid transport across the plasma membrane | 17 | 34 | 1.25E-09 | 1.84E-07 |
| Translation | 17 | 35 | 1.92E-09 | 2.43E-07 |
| Na+/Cl- dependent neurotransmitter transporters | 16 | 31 | 2.55E-09 | 2.80E-07 |
| Disorders of transmembrane transporters | 27 | 97 | 4.96E-09 | 4.87E-07 |
| Transport of small molecules | 42 | 217 | 6.84E-09 | 6.02E-07 |
| Transport of inorganic cations/anions and amino acids/oligopeptides | 20 | 57 | 1.56E-08 | 1.25E-06 |
| Transport of bile salts and organic acids, metal ions and amine compounds | 23 | 76 | 1.79E-08 | 1.30E-06 |
| Defective SLC6A19 causes Hartnup disorder (HND) | 10 | 13 | 7.83E-08 | 4.93E-06 |
| Defective SLC6A19 causes Hartnup disorder (HND) | 10 | 13 | 7.83E-08 | 4.93E-06 |
| Variant SLC6A14 may confer susceptibility towards obesity | 10 | 15 | 2.88E-07 | 1.67E-05 |
| Tryptophan catabolism | 11 | 34 | 6.64E-05 | 3.65E-03 |
| Glutamate and glutamine metabolism | 9 | 25 | 1.42E-04 | 7.22E-03 |
| Alanine metabolism | 4 | 4 | 2.72E-04 | 1.34E-02 |
| Urea cycle | 7 | 22 | 1.59E-03 | 7.32E-02 |
| Disease | 40 | 336 | 1.93E-03 | 8.49E-02 |
| Phenylalanine and tyrosine metabolism | 8 | 32 | 3.33E-03 | 1.40E-01 |
| Proton-coupled neutral amino acid transporters | 3 | 4 | 3.70E-03 | 1.48E-01 |
| Carboxyterminal post-translational modifications of tubulin | 4 | 9 | 5.21E-03 | 1.88E-01 |
| Regulation of cholesterol biosynthesis by SREBP (SREBF) | 4 | 9 | 5.21E-03 | 1.88E-01 |
| Transport of nucleosides and free purine and pyrimidine bases across the plasma membrane | 6 | 21 | 5.71E-03 | 2.00E-01 |
| RORA activates gene expression | 3 | 5 | 6.85E-03 | 2.19E-01 |
| BMAL1:CLOCK,NPAS2 activates circadian gene expression | 3 | 5 | 6.85E-03 | 2.19E-01 |
| Transport of vitamins, nucleosides, and related molecules | 11 | 64 | 1.02E-02 | 2.69E-01 |
| G alpha (q) signalling events | 11 | 64 | 1.02E-02 | 2.69E-01 |
| Synaptic adhesion-like molecules | 2 | 2 | 1.04E-02 | 2.69E-01 |
| Protein-protein interactions at synapses | 2 | 2 | 1.04E-02 | 2.69E-01 |
| Negative regulation of NMDA receptor-mediated neuronal transmission | 3 | 6 | 1.12E-02 | 2.69E-01 |
| Neurotransmitter uptake and metabolism In glial cells | 3 | 6 | 1.12E-02 | 2.69E-01 |
| Astrocytic Glutamate-Glutamine Uptake And Metabolism | 3 | 6 | 1.12E-02 | 2.69E-01 |
| Activation of gene expression by SREBF (SREBP) | 3 | 6 | 1.12E-02 | 2.69E-01 |
| Intestinal saccharidase deficiencies | 3 | 6 | 1.12E-02 | 2.69E-01 |
| Neurotransmitter release cycle | 9 | 48 | 1.17E-02 | 2.69E-01 |
| Aspartate and asparagine metabolism | 6 | 25 | 1.27E-02 | 2.93E-01 |
| Developmental Biology | 7 | 33 | 1.37E-02 | 3.02E-01 |
| Digestion | 8 | 41 | 1.38E-02 | 3.03E-01 |
| Defective SLC29A3 causes histiocytosis-lymphadenopathy plus syndrome (HLAS) | 3 | 7 | 1.68E-02 | 3.54E-01 |
| Glutathione synthesis and recycling | 4 | 13 | 1.80E-02 | 3.68E-01 |
| Metabolism of proteins | 27 | 236 | 1.98E-02 | 3.68E-01 |
| Digestion and absorption | 8 | 44 | 2.01E-02 | 3.68E-01 |
| Citric acid cycle (TCA cycle) | 6 | 28 | 2.10E-02 | 3.68E-01 |
| Defective SLC36A2 causes iminoglycinuria (IG) and hyperglycinuria (HG) | 2 | 3 | 2.24E-02 | 3.68E-01 |
| Defective GCLC causes Hemolytic anemia due to gamma-glutamylcysteine synthetase deficiency (HAGGSD) | 2 | 3 | 2.24E-02 | 3.68E-01 |
| Phenylalanine metabolism | 5 | 21 | 2.30E-02 | 3.68E-01 |
| Branched-chain amino acid catabolism | 7 | 37 | 2.39E-02 | 3.68E-01 |
| Conjugation of carboxylic acids | 4 | 15 | 2.85E-02 | 3.68E-01 |
| Amino Acid conjugation | 4 | 15 | 2.85E-02 | 3.68E-01 |
| Tyrosine catabolism | 4 | 15 | 2.85E-02 | 3.68E-01 |
| Creatine metabolism | 4 | 15 | 2.85E-02 | 3.68E-01 |
| Defective SLC27A4 causes ichthyosis prematurity syndrome (IPS) | 3 | 9 | 3.21E-02 | 3.68E-01 |
| EPHB-mediated forward signaling | 3 | 9 | 3.21E-02 | 3.68E-01 |
| Transcriptional regulation of white adipocyte differentiation | 3 | 9 | 3.21E-02 | 3.68E-01 |
| EPH-Ephrin signaling | 3 | 9 | 3.21E-02 | 3.68E-01 |
| Digestion of dietary carbohydrate | 4 | 16 | 3.48E-02 | 3.68E-01 |
| Serine biosynthesis | 4 | 16 | 3.48E-02 | 3.68E-01 |
| Transmission across Chemical Synapses | 10 | 68 | 3.60E-02 | 3.68E-01 |
| Neuronal System | 10 | 68 | 3.60E-02 | 3.68E-01 |
| Gluconeogenesis | 6 | 32 | 3.67E-02 | 3.68E-01 |
| Defective SLC3A1 causes cystinuria (CSNU) | 2 | 4 | 3.79E-02 | 3.68E-01 |
| Defective SLC7A9 causes cystinuria (CSNU) | 2 | 4 | 3.79E-02 | 3.68E-01 |
| Transport of fatty acids | 3 | 10 | 4.17E-02 | 3.68E-01 |
| Axon guidance | 4 | 17 | 4.19E-02 | 3.68E-01 |
| Nervous system development | 4 | 17 | 4.19E-02 | 3.68E-01 |
| Glucose metabolism | 7 | 42 | 4.28E-02 | 3.68E-01 |
| Serotonin Neurotransmitter Release Cycle | 2 | 5 | 5.64E-02 | 3.68E-01 |
| Hh mutants that don't undergo autocatalytic processing are degraded by ERAD | 2 | 5 | 5.64E-02 | 3.68E-01 |
| Hh mutants abrogate ligand secretion | 2 | 5 | 5.64E-02 | 3.68E-01 |

**Table S9:** Reactome pathways found from CSF metabolites detected at the 10% FDR level. Entities found are the entered proteins, total entities are the reported proteins in the pathway. Pathways were cut off at p = 0.05.

| **Pathway name** | **#Entities found** | **#Entities total** | **Entities p-value** | **Entities FDR** |
| --- | --- | --- | --- | --- |
| tRNA Aminoacylation | 16 | 26 | 1.68E-11 | 4.67E-09 |
| Mitochondrial tRNA aminoacylation | 16 | 26 | 1.68E-11 | 4.67E-09 |
| Cytosolic tRNA aminoacylation | 16 | 26 | 1.68E-11 | 4.67E-09 |
| Translation | 16 | 35 | 1.21E-09 | 2.52E-07 |
| Amino acid transport across the plasma membrane | 15 | 34 | 6.64E-09 | 1.08E-06 |
| SLC-mediated transmembrane transport | 32 | 163 | 8.23E-09 | 1.08E-06 |
| SLC transporter disorders | 22 | 80 | 9.09E-09 | 1.08E-06 |
| Defective SLC6A19 causes Hartnup disorder (HND) | 10 | 13 | 1.62E-08 | 1.49E-06 |
| Defective SLC6A19 causes Hartnup disorder (HND) | 10 | 13 | 1.62E-08 | 1.49E-06 |
| Transport of bile salts and organic acids, metal ions and amine compounds | 21 | 76 | 1.92E-08 | 1.59E-06 |
| Transport of inorganic cations/anions and amino acids/oligopeptides | 18 | 57 | 3.12E-08 | 2.34E-06 |
| Disorders of transmembrane transporters | 23 | 97 | 5.99E-08 | 4.14E-06 |
| Na+/Cl- dependent neurotransmitter transporters | 13 | 31 | 1.28E-07 | 8.22E-06 |
| Transport of small molecules | 35 | 217 | 1.88E-07 | 1.11E-05 |
| Variant SLC6A14 may confer susceptibility towards obesity | 8 | 15 | 6.81E-06 | 3.74E-04 |
| Glutamate and glutamine metabolism | 9 | 25 | 3.95E-05 | 2.06E-03 |
| Tryptophan catabolism | 9 | 34 | 3.89E-04 | 1.90E-02 |
| Urea cycle | 7 | 22 | 6.07E-04 | 2.79E-02 |
| Phenylalanine and tyrosine metabolism | 8 | 32 | 1.18E-03 | 5.08E-02 |
| Alanine metabolism | 3 | 4 | 2.33E-03 | 9.07E-02 |
| Proton-coupled neutral amino acid transporters | 3 | 4 | 2.33E-03 | 9.07E-02 |
| Carboxyterminal post-translational modifications of tubulin | 4 | 9 | 2.90E-03 | 1.07E-01 |
| Aspartate and asparagine metabolism | 6 | 25 | 5.91E-03 | 2.13E-01 |
| Negative regulation of NMDA receptor-mediated neuronal transmission | 3 | 6 | 7.15E-03 | 2.13E-01 |
| Neurotransmitter uptake and metabolism In glial cells | 3 | 6 | 7.15E-03 | 2.13E-01 |
| Astrocytic Glutamate-Glutamine Uptake And Metabolism | 3 | 6 | 7.15E-03 | 2.13E-01 |
| Proton-coupled monocarboxylate transport | 3 | 6 | 7.15E-03 | 2.13E-01 |
| Synaptic adhesion-like molecules | 2 | 2 | 7.62E-03 | 2.13E-01 |
| Protein-protein interactions at synapses | 2 | 2 | 7.62E-03 | 2.13E-01 |
| Metabolism of proteins | 25 | 236 | 8.32E-03 | 2.25E-01 |
| Glutathione synthesis and recycling | 4 | 13 | 1.04E-02 | 2.70E-01 |
| Metabolism of amino acids and derivatives | 28 | 280 | 1.10E-02 | 2.85E-01 |
| Phenylalanine metabolism | 5 | 21 | 1.21E-02 | 3.02E-01 |
| Defective SLC36A2 causes iminoglycinuria (IG) and hyperglycinuria (HG) | 2 | 3 | 1.64E-02 | 3.48E-01 |
| Defective GCLC causes Hemolytic anemia due to gamma-glutamylcysteine synthetase deficiency (HAGGSD) | 2 | 3 | 1.64E-02 | 3.48E-01 |
| Tyrosine catabolism | 4 | 15 | 1.67E-02 | 3.48E-01 |
| Creatine metabolism | 4 | 15 | 1.67E-02 | 3.48E-01 |
| Digestion | 7 | 41 | 1.75E-02 | 3.48E-01 |
| Synthesis of Ketone Bodies | 4 | 16 | 2.06E-02 | 3.48E-01 |
| Metabolism of carbohydrates | 17 | 154 | 2.07E-02 | 3.48E-01 |
| EPHB-mediated forward signaling | 3 | 9 | 2.10E-02 | 3.48E-01 |
| EPH-Ephrin signaling | 3 | 9 | 2.10E-02 | 3.48E-01 |
| Digestion and absorption | 7 | 44 | 2.46E-02 | 3.48E-01 |
| Axon guidance | 4 | 17 | 2.50E-02 | 3.48E-01 |
| Nervous system development | 4 | 17 | 2.50E-02 | 3.48E-01 |
| Defective SLC7A9 causes cystinuria (CSNU) | 2 | 4 | 2.80E-02 | 3.48E-01 |
| Defective SLC3A1 causes cystinuria (CSNU) | 2 | 4 | 2.80E-02 | 3.48E-01 |
| Ketone body metabolism | 4 | 18 | 2.99E-02 | 3.48E-01 |
| Purine ribonucleoside monophosphate biosynthesis | 6 | 37 | 3.36E-02 | 3.48E-01 |
| Branched-chain amino acid catabolism | 6 | 37 | 3.36E-02 | 3.48E-01 |
| Citric acid cycle (TCA cycle) | 5 | 28 | 3.59E-02 | 3.48E-01 |
| Disease | 30 | 336 | 3.60E-02 | 3.48E-01 |
| Hh mutants that don't undergo autocatalytic processing are degraded by ERAD | 2 | 5 | 4.20E-02 | 3.48E-01 |
| Hh mutants abrogate ligand secretion | 2 | 5 | 4.20E-02 | 3.48E-01 |
| Defective SLC16A1 causes symptomatic deficiency in lactate transport (SDLT) | 2 | 5 | 4.20E-02 | 3.48E-01 |
| Pyrimidine biosynthesis | 4 | 21 | 4.80E-02 | 3.48E-01 |


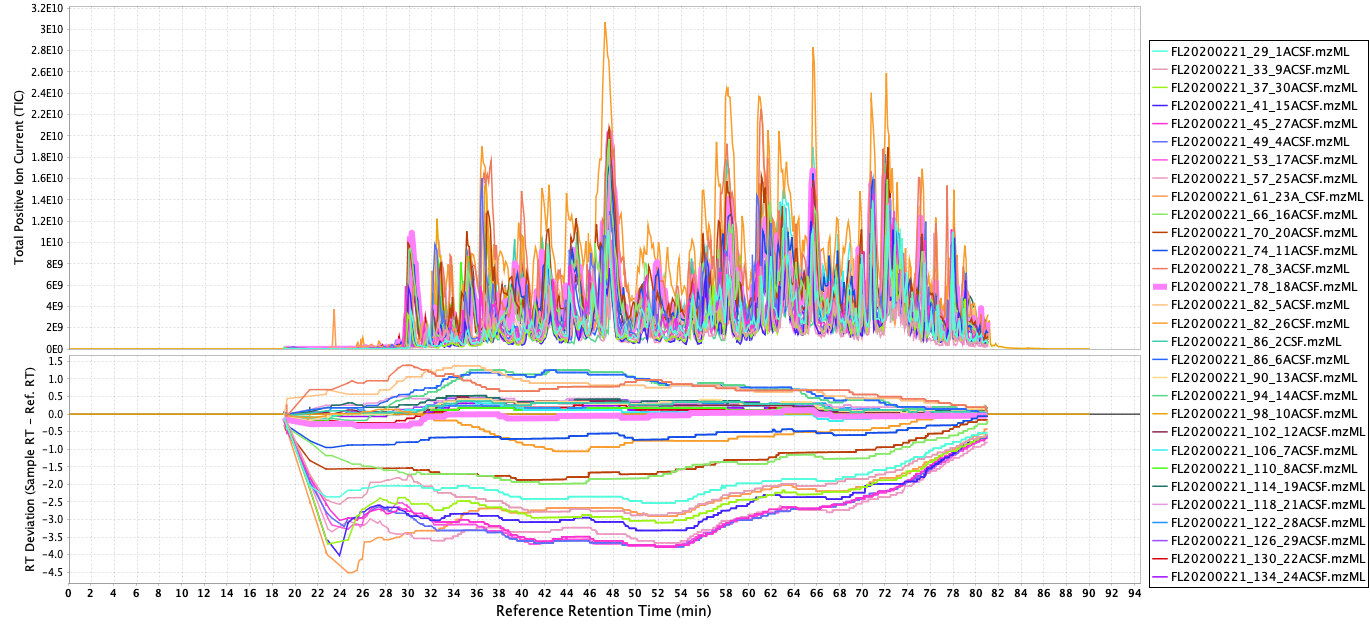


**Figure S1:** Total ion chromatograms from proteomic analysis of the 30 CSF samples.


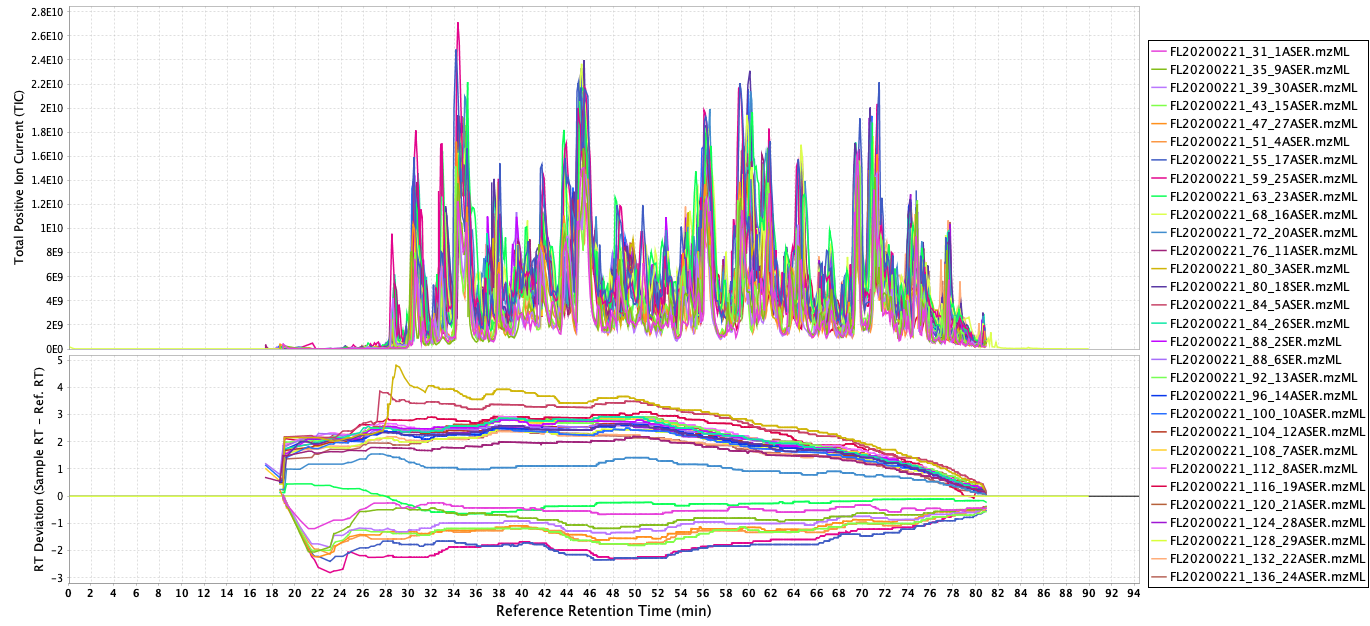


**Figure S2:** Total ion chromatograms from proteomic analysis of the 30 serum samples.

**Table S10:** PrecisionMed sample banking Inclusion/Exclusion Criteria. To qualify subjects must answer yes to all inclusion criteria and no to all exclusion criteria. The subject must also pass the M.I.N.I Plus 5.0.0 mental examination.

| Inclusion Criteria | Exclusion Criteria |
| --- | --- |
| - Negative urine screen for nicotine/negative urine pregnancy test in females. | - Evidence of depression, any affective disorder (e.g. bipolar or schizophrenia), diabetes, sever hypertension, renal or hepatic impairment, dementia, movement disorder, drug intoxication, thyroid disease, chronic infections, or pregnancy. |
| - Subject signs approved written informed consent prior to any study procedures. | - Night-Shift worker. |
| - Subjects must agree to venipuncture +/- 50 mL on 9 occasions in 60 hours, spinal tap for < 25 mL of CSF every 2-4 weeks. Additional blood will be drawn for chemistry and hematology as well as pharmacokinetics. | - Subject has donated blood or plasma within 1 month prior to Visit 1. |
| - Female and male subjects age at entry into collection 20-80 years inclusive. | - Subject has started on any new medications within 2 weeks prior to Visit 1. |
| - Subjects must have a BMI of 18.5-24.9 inclusive. | - Uncontrolled hypertension, insulin dependence or uncontrolled diabetes mellitus. |
| - Subjects must be non-smokers for at least 1 year and must not be using nicotine supplements. | - History of substance abuse within 6 months of screening. |
| - Subject is of Caucasian descent. | - Subject shows evidence of chronic disease. |
| - Subject has not participated in any previous PrecisionMed banking studies. | - Subject has any significant cardiac, malignant, neurological, bleeding or other episode within 2 years. |
| - Subject is on normal sleep/wake cycle for at least 2 weeks prior to enrollment. | - Subject has a history of significant insomnia or sleep disorder. |
| - Subject has no family history of Alzheimer’s disease. | - History of major psychiatric disorder described in DSM-IV within the last 2 years. |
| - Subject has no history of neuropsychiatric disease. |  |
| - Subject has not taken aspirin containing products nor anti-inflammatory products within 1 week of Visit. |  |

**Figure S3:** Protein concentration in each sample determined by BCA assay.
